# Flexible perovskite solar cells: advancements in materials, fabrication techniques, and future prospects

**DOI:** 10.1186/s40580-025-00524-y

**Published:** 2025-11-28

**Authors:** Muhammad H. Nawaz, The-Hung Mai, Sang I. I. Seok, Feng-Chuan Chuang, Phuong V. Pham, Nam-Gyu Park

**Affiliations:** 1 Department of Physics, National Sun Yat-Sen University, Kaohsiung, 80424 Republic of China; 2 School of Energy and Chemical Engineering, Ulsan National Institute of Science and Technology (UNIST), Ulsan, 689-798 South Korea; 3 Department of Physics, National Tsing Hua University, Hsinchu, 30013 Republic of China; 4 Physics Division, National Center for Theoretical Sciences, Hsinchu, 30013 Republic of China; 5 School of Chemical Engineering and Center for Antibonding Regulated Crystals, Sungkyunkwan University, Suwon, 16419 South Korea; 6 SKKU Institute of Energy Science and Technology (SIEST), Sungkyunkwan University, Suwon, 16419 South Korea

**Keywords:** Flexible solar cells, Perovskites, Fabrication, Carbon-based materials, R2R fabrication

## Abstract

Flexible solar cells (FSCs) are a revolutionary photovoltaic innovation that possesses superior power conversion efficiencies greater than 26.7%, cost-effective production techniques, and intrinsic integration with large-scale manufacturing processes. Among various FSC technologies, flexible perovskite solar cells (FPSCs) are one of the top candidates for commercialization due to their suitability for roll-to-roll (R2R) printing techniques, making it simple to operate on a mass production scale. This review compiles an extensive summary of the advances made in FPSCs over the past few years, particularly focusing on FPSCs, examining their recent advances and performance metrics of flexible photovoltaic systems, silicon-based, dye-sensitized, organic, quantum dot, and hybrid technologies. Detailed overview of the most important components of FPSCs i.e. flexible substrates, perovskite absorber layers, charge transport materials, processing techniques, and encapsulation strategies are provided. Each material is discussed in terms of impact on device performance, efficiency, and longevity with the aim of overcoming the challenge which prevents their commercialization. Eventually, the discussion covers the future prospects of FPSCs, strategies for boosting their lab-scale performance and their potential impact on the development of flexible energy-harvesting technologies.

**Graphical abstract:**

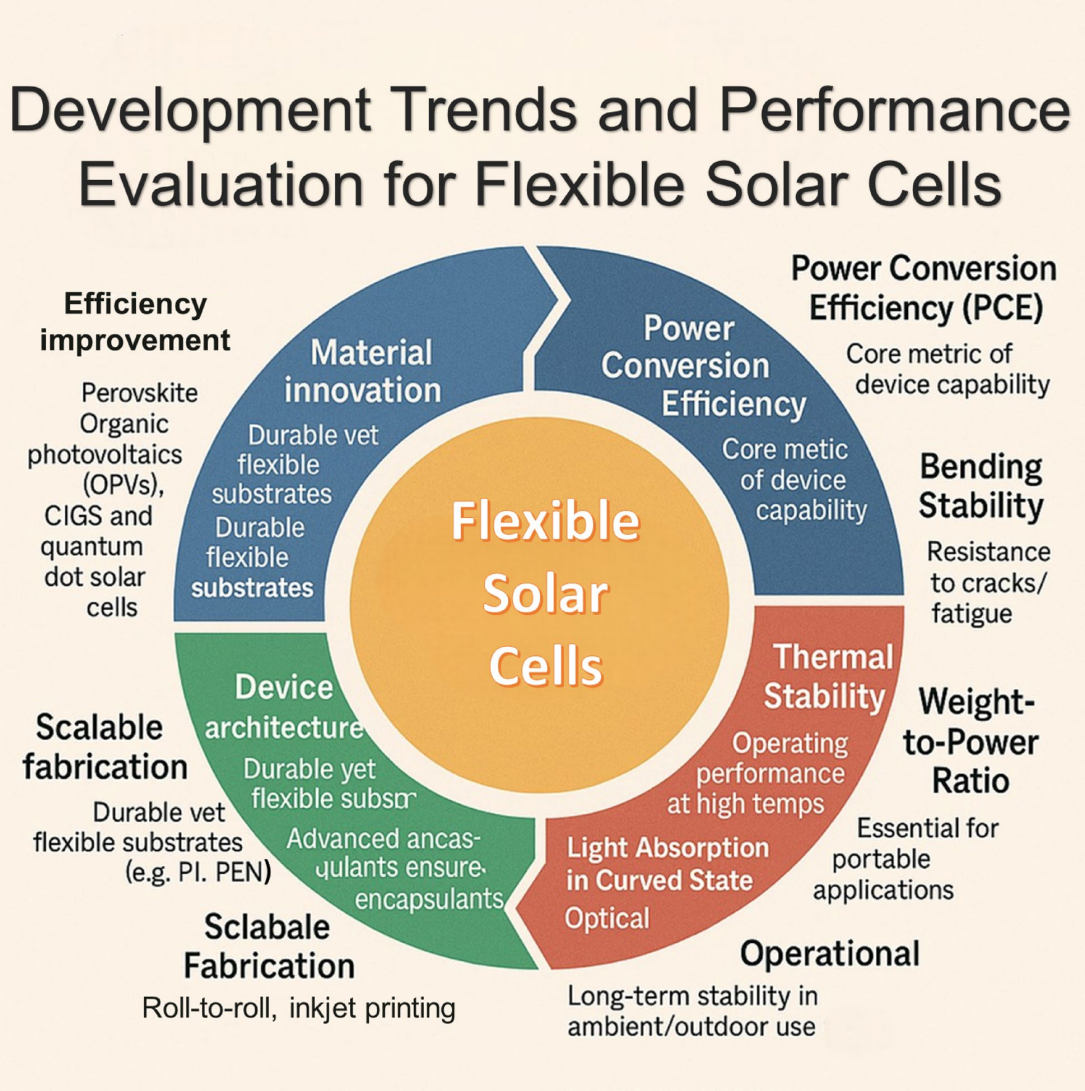

## Introduction

The twenty-first century has brought flexible solar cells (FSCs) into the scientific spotlight because of their outstanding optoelectronic properties, low manufacturing costs, high-power conversion efficiencies (PCEs) and wide spectral light absorption capabilities. Tsutomu Miyasaka along with his team members introduced the first dye-sensitized solar cells (DSCs) in 2009 with the FTO/TiO_2_/CH_3_NH_3_Pb (I, Br)_3_/Li (I, Br)/Pt/FTO configuration which started at 3.8% PCE and reached 6.5% by 2011. The perovskite materials showed chemical instability when used with liquid electrolytes, which created a significant obstacle for the initial development of PSCs. Park et al. addressed the primary challenge by substituting the liquid electrolyte with an organic solid-state hole-transport layer. The essential breakthrough enhanced both the device’s structural durability and its operational life span. By reaching 10% efficiency, the team’s solid-state perovskite solar cell pushed the technology into a new era science [1]. Snaith’s research group independently validated the potential of perovskite solar cells through the use of organic hole conductors that incorporated into meso-superstructured scaffolds establishing architectural feasibility of perovskite designing [2]. Seok’s team simultaneously achieved a groundbreaking accomplishment through their first report of heterojunction solar cells by utilizing polymeric hole conductors with perovskite [3]. 

**Fig. 1:**
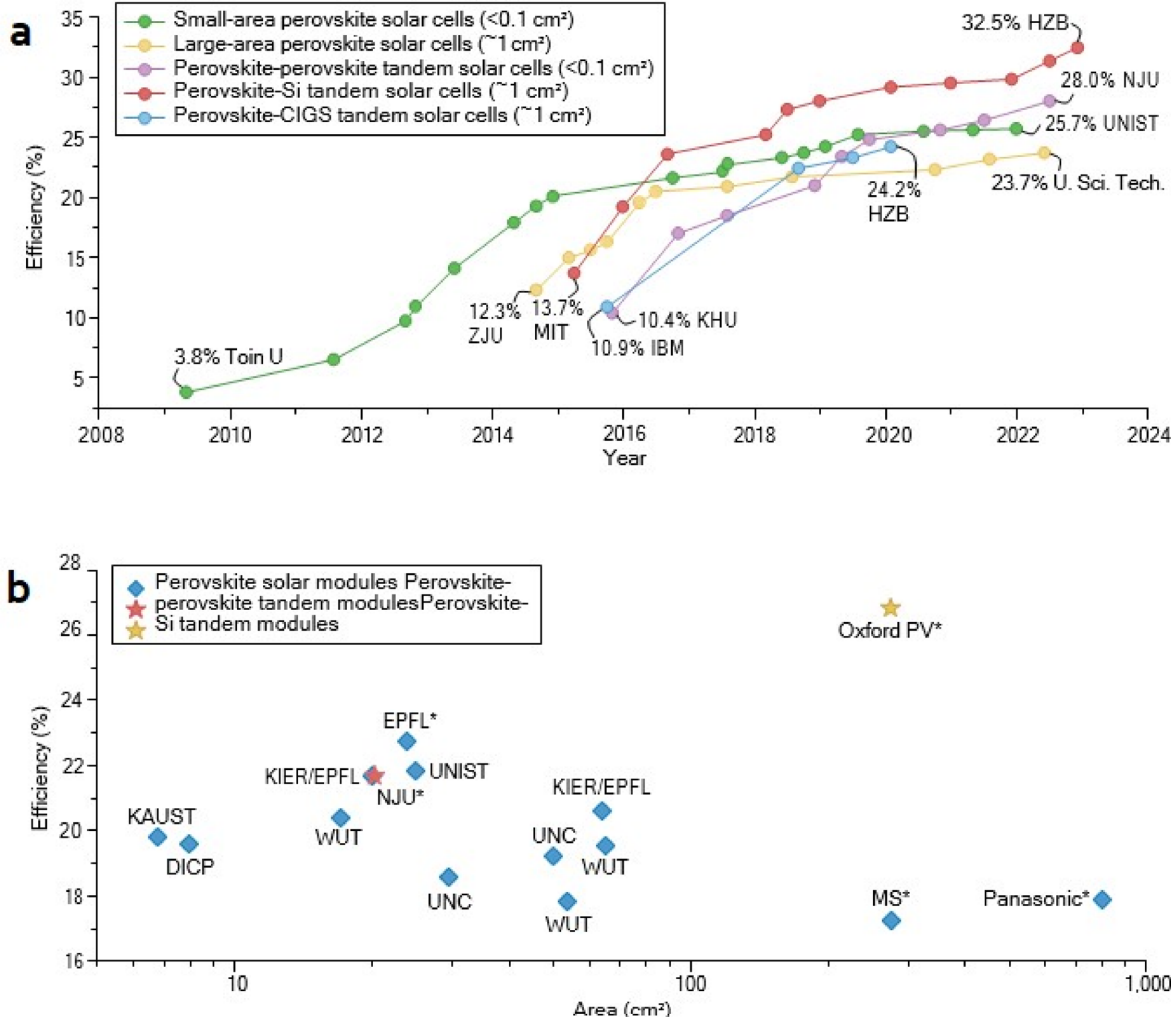
**a** The PCE of perovskite solar cells (PSCs) shows its evolution through different dimensions and compositions with all occurrences surpassing PCE. The highest PCE recorded in each category is also emphasized, along with the corresponding research institute and certification status. **b** Perovskite’s cost-effectiveness and long-term stability hold promise for next-generation PVs, with current successes tied to FAPbI_3_-rich perovskites. Reproduced with permission from ref [9]. Copyright 2023 Springer Nature

Figure 1a provides a general timeline of the development of PSC efficiency from 2008 to 2024, illustrating the remarkable advancements in rapidly expanding research field of solar cells. Data is divided into different types of PSCs, including small-area, large-area, perovskite-perovskite tandem, perovskite-Si tandem, and perovskite-CIGS tandem solar cells. Each type is represented by distinctive color-coded curves with highlighted significant milestones for each category [4]. Starting 2008, Toin University (Toin U) was able to attain its first 3.8% efficiency in small-area perovskite solar cells. Zhejiang University (ZJU) was at 12.3% in 2010, then Massachusetts Institute of Technology (MIT) at 13.7% in 2012. Kyung Hee University (KHU) in 2016 was 10.4% efficient, and IBM managed to reach 10.9% the same year. The record move to tandem cells was when Helmholtz Zentrum Berlin (HZB) announced a record 32.5% efficiency for perovskite-Si tandem cells in 2022. Nanjing University (NJU) also announced 28% in 2022, and Ulsan National Institute of Science and Technology (UNIST) announced 25.7%. HZB achieved 24.2% with the perovskite-CIGS tandem cells, while the University of Science and Technology (U. Sci. Tech.) achieved a record 23.7% [5, 6]. 

Figure 1b displays a general comparison of the efficiency and area of perovskite solar modules, perovskite tandem modules, and perovskite-Si tandem modules reported by various institutions. Efficiency, ranging from 16 to 26%, is plotted against module area (ranging from 10 to 1000 cm^2^). Blue diamond icon represents perovskite solar modules, red stars perovskite tandem modules, yellow star perovskite-Si tandem modules. KAUST, DICP, KIER/EPFL, WUT, EPFL, UNIST, NJU (reporting a record 23% efficiency for a 30 cm^2^ module), UNC, WUT, MS, and Panasonic are some of the prominent institutions among the data contributors. Oxford PV stands out among them with its perovskite-Si tandem modules with nearly 26% efficiency [7, 8]. 

**Fig. 2:**
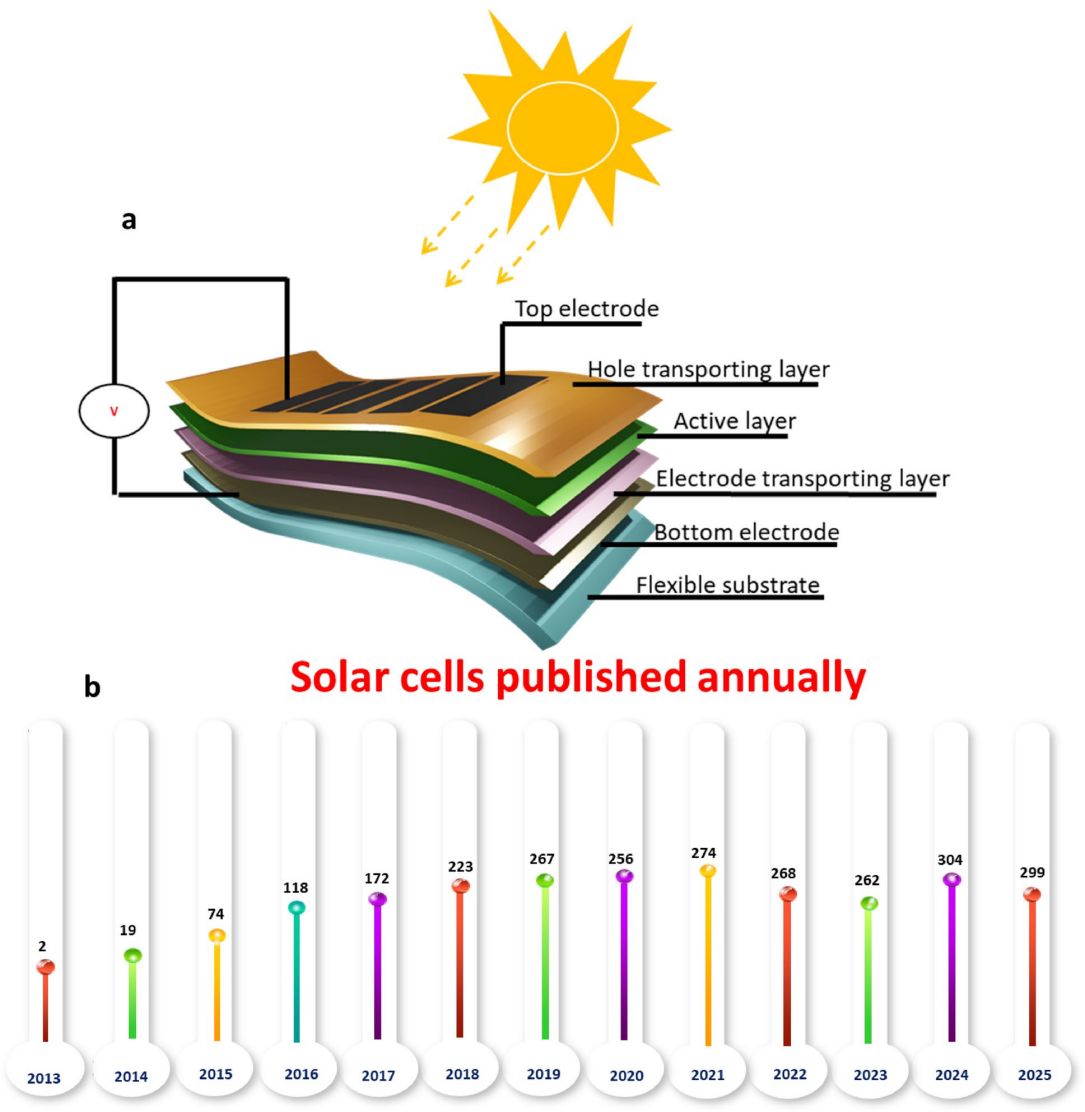
**a** Flexible solar cell is depicted schematically. **b** Annual articles published on flexible perovskite solar cells from 2010 to 2025. *Source*: Web of Science

Figure 2a depicting the functional component in FPSCs structure architecture. *Flexible Substrate*: The flexible substrate forms the bottom of the solar cell, being mechanically flexible but resistant to chemical and thermal stress. Polyethylene terephthalate (PET), polyimide (PI), and thermoplastic polyurethane (TPU) are common substrates due to their excellent resistance and processability. Substrates not only enable R2R fabrication processes but also enhance adhesion to subsequent layers by surface modifications, achieving maximum overall device performance [10]. *Bottom Electrode*: The purpose of the bottom electrode is charges transport and collection for ensuring the free movement of current in the device. The contact needs to provide low resistance and must be flexible to maintain device performance. It is designed to withstand mechanical deformation, consequently the electrical properties are maintained under stress or bending, which is critical to the flexibility of the solar cell. *Photoactive Layer*: The photoactive layer in the center of the solar cell is to absorb light and generate charge carriers. Hybrid organic-inorganic perovskites, polymer mixtures, or quantum dots are typically employed, because they have high absorption efficiency, can be modified to adjust the band gap, and are compatible with solution-based deposition processing. Superior photon absorption and charge separation are additionally improved through advanced nanostructuring and gradient-layer design to significantly enhance overall power conversion efficiency of the device. *Electron Transport Layer*: Placed adjacent to the photoactive layer, the electron transport layer (ETL) facilitates selective extraction and transport of electrons. Metal oxide materials tend to be used in ETL due to their high electron mobility and virtuous stability. Carrier mobility is improved and recombination losses reduced with surface passivation as well as chemical doping, thereby increasing the overall efficiency and device lifetime. *Hole Transport Layer*: Hole transport layer (HTL) is responsible for transporting holes efficiently and preventing electron backflow. The performance of HTL is critical in producing the best voltage and long-term operation stability. Methods such as molecular orientation control and interfacial engineering are often employed to maximize the efficiency of HTL and minimize energy losses. *Transparent Top Electrode*: The top transparent electrode serves a dual purpose, it consents light to pass to the photoactive layer and completes the electrical circuit. Transparent conductors are commonly used for their transparency and conductance. Flexible versions of these electrodes are being increasingly incorporated by techniques such as inkjet printing, retaining their functionality when subjected to mechanical stress so that the device remains operational in flexible applications [11–13]. These factors are critical components in the operational performance and longevity of flexibility solar cells. By optimizing the material attributes of and processing in the layers, and the way those layers interact with each other, devices are being designed for flexible energy harvesting solutions that are more scalable, more sustainable and higher performing [14]. 

According to the statistics in Fig. 2b there has been a remarkable increase in the number of solar cell-related publications published on the ‘Web of Science’ website between 2010 and 2025. It should be noted that the exponential growth of articles began in 2013, coinciding with the advancement of solar cell technology. This data indicates that solar cell materials have consistently remained a prominent and well-researched topic among scientists.

**Fig. 3:**
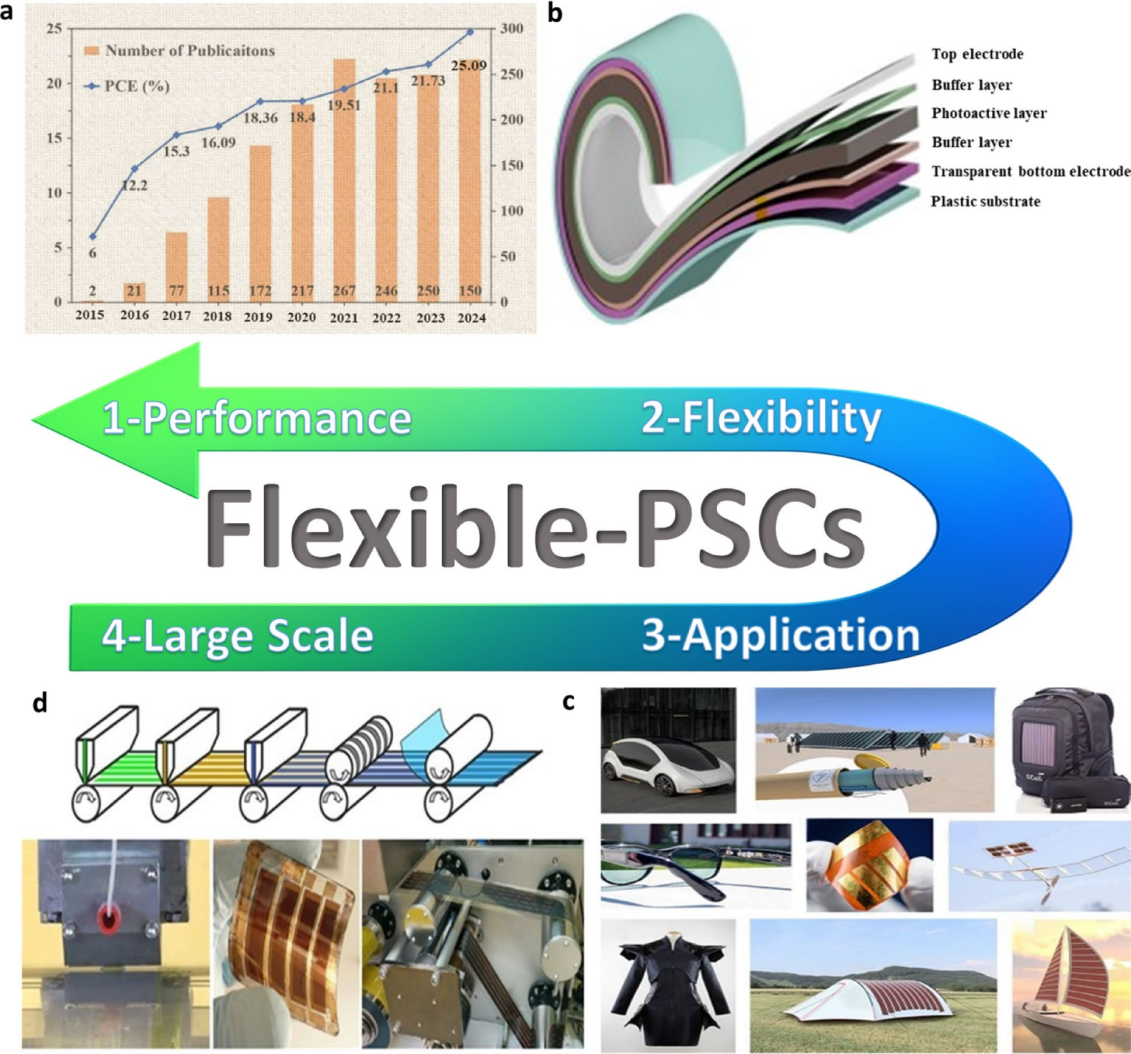
FPSCs development elements such as **a** Performance: Progress in the field of FPSCs over recent decades: Reproduced with permission from ref [25]. Copyright 2023 John Wiley & Sons. **b** Flexibility: Common architecture of FPSCs. Reproduced with permission from ref [26]. Copyright 2021 Springer Nature. **c** Applications: Diagram of an electric vehicle powered by FPSCs, packaged in tubes prepared for installation, solar-powered backpack, electric sunglasses integrated with solar cells, image of a typical laboratory-fabricated FPSC, unmanned aerial vehicle, wearable clothing incorporating solar cells, tent equipped with embedded flexible perovskite solar cells, and a watercraft powered by solar energy: Reproduced with permission from ref [27]. Copyright 2020 Elsevier. **d** Large scale production: Perovskite manufacturing processes on a large scale: Reproduced with permission from ref [28]. Copyright 2016 American Institute of Physics

Flexible solar cells have witnessed a tremendous increase in efficiency during the last decade, heralding significant advances in material science and device engineering. Figure 3a exhibiting their power PCE started from a mere 6.00% in 2015, with only 74 research articles supporting it. As efficiency climbed to 10.00% in 2016, publications more than tripled to 118. The rising trend continued: 13.60% efficiency with 172 publications in 2017, and 17.30% with 223 publications in 2018. In 2019, PCE was 19.80%, with 267 papers, followed by a sharp increase to 23.00% and 256 publications in 2020. The peak was achieved in 2021, with 24.50% efficiency and an explosion of interest marked by 274 publications. A steady progress continued with 24.79% and 268 papers in 2022, 25.00% with 262 papers in 2023, 25.09% with 304 publications in 2024, and record efficiency 26.7% with 299 publications in 2025. This incredible trajectory is a witness to a robust synergy between technological development and growing academic interest, pushing flexible solar cells towards commercialization and sustainable energy supremacy [15–17]. 

Figure 3b highlights key components that render a solar cell flexible. The plastic substrate provides a light, pliable base, without compromising structural integrity. The transparent bottom electrode allows light in while facilitating efficient conduction of charges. The buffer layer provides a protective interface that improves the transport of charges and reduces bending stress, thus ensuring the flexibility of the cell. The photoactive layer converts sunlight into electricity and is designed to be efficient and thin to make the cell flexible and performant. Finally, the upper electrodes are conductive and flexible to collect electricity without blocking the light, which is necessary to maintain the energy conversion capability of the cell. Together, these parts empower the solar cell to retain its flexibility while ensuring efficient energy production [18–20]. 

Figure 3c shows the various opportunities of FPSCs in industries, reflecting their potential in developing renewable energy sources. In electric vehicles, FPSCs incorporated to tap solar energy, providing an additional source of power for recharging and overall increased energy efficiency of the vehicle. FPSCs, tube-packaged for rapid installation, offer an easy and convenient method of deploying solar panels in any environment, from rooftops to fields. Solar-powered backpacks and electric sunglasses featuring solar cells offer on-the-go power generation for personal devices so that one can power their electronics devices with ease [21]. In aerial applications using air, U-turned flying machines leverage the light and flexible nature of FPSCs to deliver clean power for prolonged flight time. Wearable clothing with integrated FPSCs gives an unparalleled innovation, with clothing collecting power without affecting comfort and flexibility. Moreover, energy from the sun in outdoor settings is realizable as demonstrated by tents embedded with FPSCs, providing off-grid power for fieldwork or camping. Conclusively, a solar boat is used in water to reduce the use of fossil fuels. These decisive advantages of FPSCs in such environments parade their lightness, flexibility, and efficiency, making them suitable for incorporation into a variety of products environments, yielding sustainable and renewable energy supplies for household systems [22, 23]. 

Figure 3d shows important processes for the large-scale industrial manufacturing of FPSCs, that is key prerequisite for the commercialization of solar energy technologies. R2R processing method approaches for the efficient high-volume manufacturing of flexible solar cells. As these processes continue to advance, they can reduce production costs, increase access to renewable energy, and contribute to meeting global energy demands in a sustainable manner [24]. This review aims to delve into more detail about the development trend and prospects of FPSCs at large industrial scale, their challenges and integration in wearable electronics (i.e., T-shirt, cap, jacket, shoes), and their application in low-light environments (i.e., dark forest areas, submarine underwater, ocean gate).

## Overview and basic principles of flexible solar cell

This framework gives the major concepts and developments in flexible solar cell technology. It explains the evolution of flexible perovskite solar cells and identifies the major materials and components behind their performance. The focus is on the use of flexible substrates, efficient charge carrier layers, and transparent electrodes in optimizing the functionality of the solar cells.

### Evolution and state of the art flexible perovskite solar cells

**Fig. 4:**
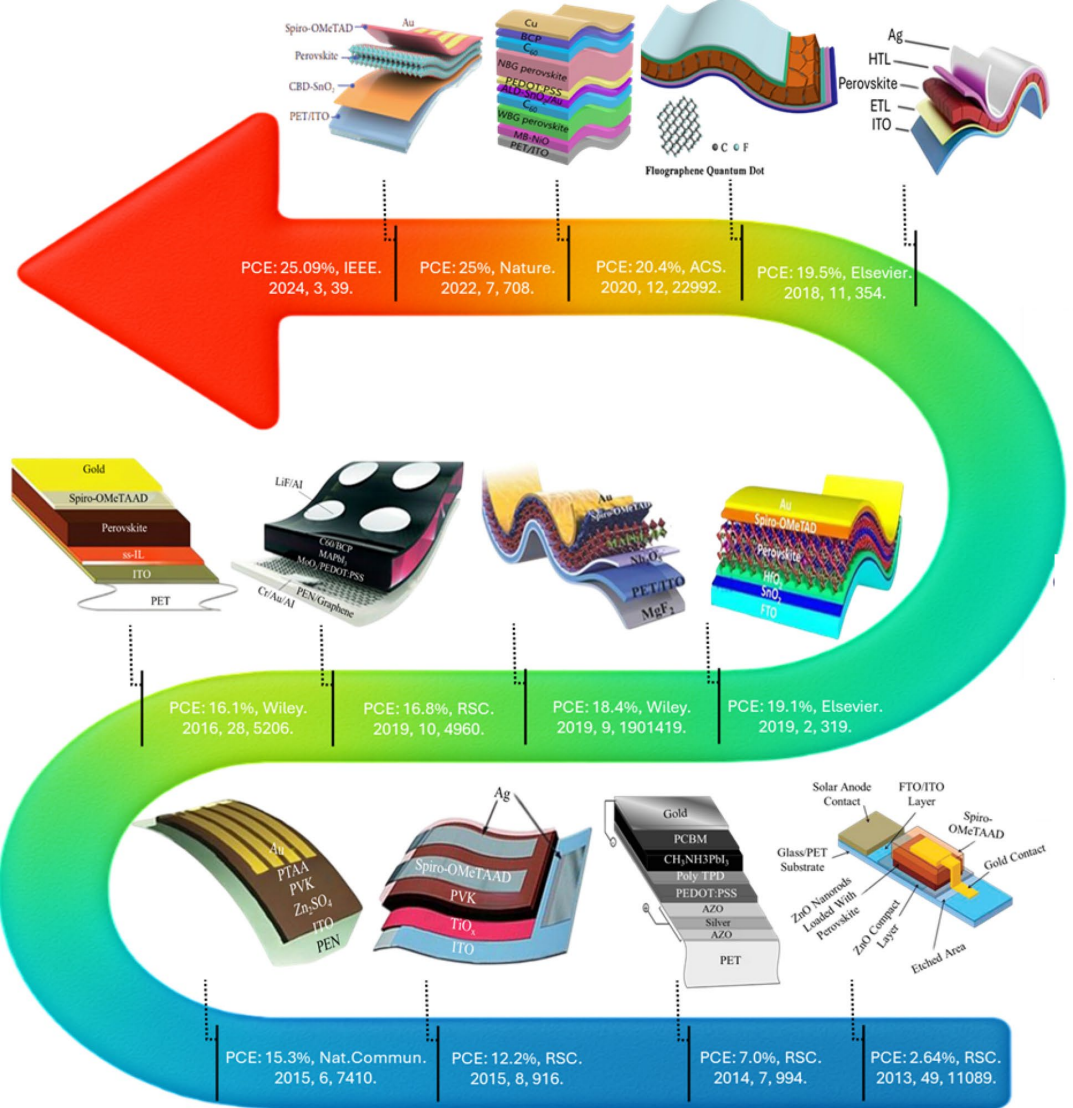
FPSCs of efficiency from 2013 to 2023: first FPSCs 2.62%, AZO-Ag-AZO electrode 7%, TiO_2_ prepared 12.2%, Zn_2_SO_4_ prepared 15.3%, Ionic liquid ETM 16.1%, graphene electrode 16.8%, dimethyl sulfide 18.4%, quintuple cation perovskite 19.1%, price control ETM 19.5%. Reproduced with permission from ref [27]. Copyright 2020 Elsevier. Quantum dot solar cells have achieved an efficiency of 20.40%. Reproduced with permission from ref [29]. Copyright 2020 American Chemical Society. NiO nanocrystalline film 25%. Reproduced with permission from ref [33]. Copyright 2022 Springer Nature. ITO/PET HTL base solar cell 25.09%. Reproduced with permission from ref [34]. Copyright 2024 IEEE

FPSCs were first developed by Gratzel in 2013 with the initial PCE of 2.6% on polyethylene terephthalate (PET) substrates. After that, significant work devoted to optimizing flexible conducting substrates, enhancing charge transport layers, and device architecture engineering to enhance performance. Considerable progress was achieved globally to advance FPSC technology. Snaith’s team set the foundation by employing poly(3,4 ethylenedioxythiophene): polystyrene sulfonate (PEDOT: PSS) as a transparent electrode to achieve a PCE of 7% in 2014 [29]. Yang’s team again developed low-temperature fabrication technologies that are PET/ITO substrate compatible, improving the efficiency to 9.2%. Kim further improved electron transport by adding amorphous titanium oxide using atomic layer deposition, which resulted in a PCE of 12.2%. Seok enhanced this again with Zn_2_SnO_4_ nanoparticles as the electron transport layer (ETL) and achieved an efficiency of 15.3% retaining over 95% of the original performance after 300 bending cycles [30]. Congruently, flexible graphene electrode work by Choi was able to bring up the record PCE to 16.8%, and implementation of mesoporous SnO_2_ by Seo for various enhanced electron extraction processes finally takes it to 20.7%. Miyasaka introduced artemisinin passivation for enhanced device stability with a PCE of 21.15%. Jen’s group extended the absorption window by employing a narrow bandgap organic heterojunction to achieve an efficiency of 21.73% and later further improved charge extraction by utilizing a pentyl ammonium acetate interlayer, pushing the efficiency up to 23.35%. Several studies in China were also revolutionary [31]. Zhao improves the quality of the perovskite thin film and incorporates lead thiocyanate to fabricate the PET/ITO-based devices. These devices produce a 17.96% PCE. Liu, by optimizing perovskite crystallization with the use of dimethyl sulfide, obtained large-grain films with 18.4% efficiency [32]. Yang optimized SnO_2_ ETL morphology and electrical properties to obtain a 19.5% record. In 2022 Mai achieved 20.16% using 2D/3D perovskite heterojunctions, while Shi constructed sophisticated heterojunctions using 3-chlorobenzylammonium iodide and achieved a PCE of 21.4%. Li reported NiO based interfaces in 2022 to achieve further enhanced performance with 25.0%. Ren recently recorded the highest PCE of 25.09% using SnSO_4_ in the perovskite precursor and depositing it with finely tuned control for SnO_2_ growth [33]. This record is the highest efficiency to date for flexible perovskite solar cells as observed in Fig. 4. As the efficacy of flexible solar cells increases, they significantly enhance their viability for wearable electronics, portable power sources, and building-integrated photovoltaics. Greater efficiency equates to more power from light, flexible devices, promoting their commercialization and impacting sustainable energy tenders [34]. 

### Core materials for flexible solar cell design

**Fig. 5:**
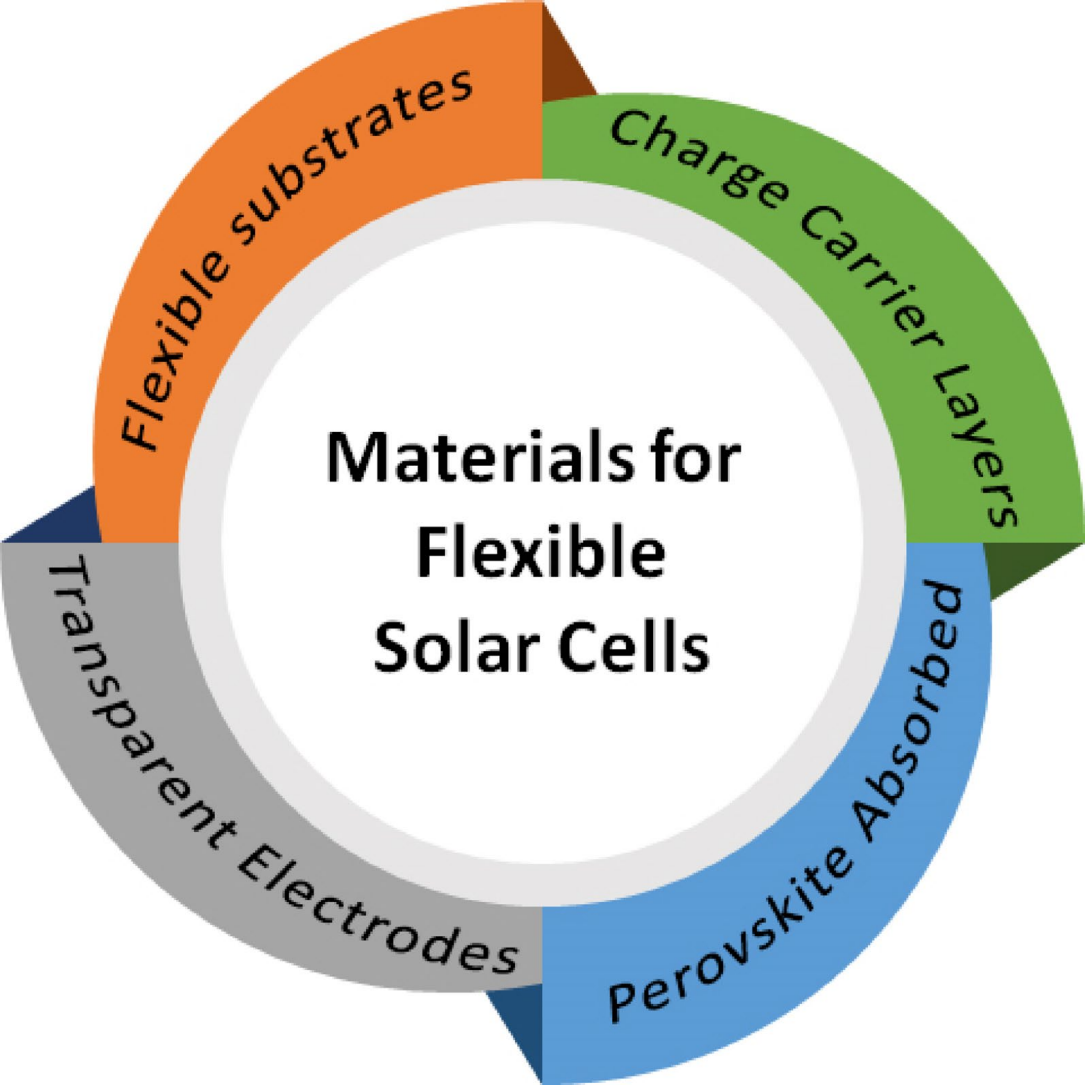
Materials for highly efficient flexible solar cells

The substances utilized in flexible solar cells determine their efficiency and performance. The most consequential of these are flexible substrates, charge carrier layers, perovskite absorbers, and transparent electrodes, all of which play a role to provide the functionality to the cell show in Fig. 5. The integration of these substances enhanced the flexibility and energy conversion efficiency of the solar cells [35]. 

#### Flexible substrates

**Fig. 6:**
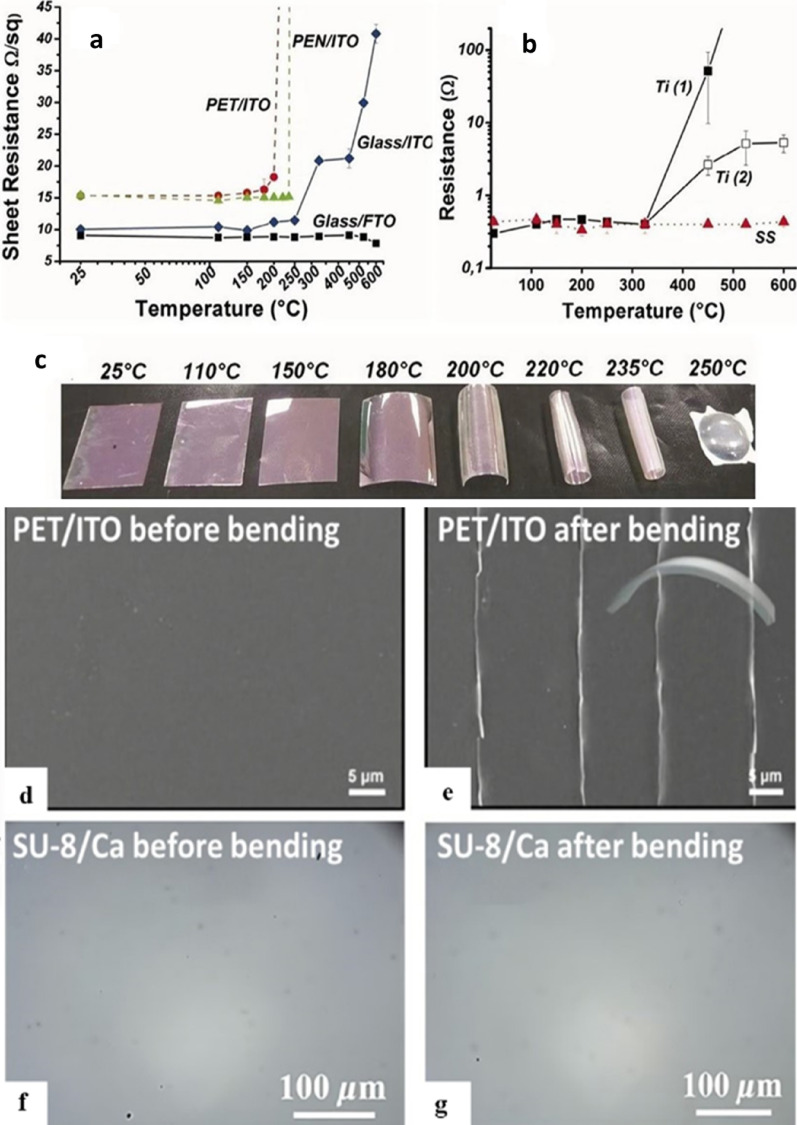
Substrates for 30 min under different temperatures **a** plastic substrates (PET/ITO or PEN/ITO), **b** metallic substrates (Ti) and stainless steel (SS)). **c** Deformation of PEN/ITO after thermal treatment. Reproduced with permission from ref [39]. Copyright 2011 Wiley & Sons. A PET/ITO substrate before and after bending **d**,** e** Before and **f** after **g** bending of an SU-8/Ca substrate: Reproduced with permission from ref [27]. Copyright 2020 Elsevier

The type of substrate mostly determines the flexibility of solar cells. Typically classified as either metallic or plastic substrates are substrates. Materials like titanium, copper foils, and stainless steel are considered metallic; plastic substrates are usually made from polymers like PET or PEN. Although thermal stability is still a constraint, plastic substrates have several benefits including being inexpensive, having great optical clarity, great flexibility, and chemical resistance [36]. Figure 6a sheet resistance (Ω/sq) of various substrates (PET/ITO, PEN/ITO, Glass/ITO, Glass/FTO) as a function of temperature. PET/ITO and PEN/ITO have a very high sheet resistance with rising temperature, particularly beyond 200 °C, but Glass/ITO and Glass/FTO remain stable in terms of resistance profile. Figure 6b is the resistance (Ω) of titanium (Ti) and stainless steel (SS) versus temperature. Ti (both Ti( 1) and Ti(2)) exhibits a very steep rise in resistance after approximately 400 °C, and that of Ti(2) rises more steeply than that of Ti(1). SS has relatively uniform resistance values across the temperature range. Figure 6c shows that plastic substrates begin to deform between 150 and 250 °C, accompanied by an increase in sheet resistance [27]. 

Figure 6d parades the PET/ITO architecture before bending when the ITO layer is smooth and intact. However, as shown in Fig. 6e upon bending cracks in the ITO layer. These cracks, being a result of mechanical stress upon bending, lead to a significant reduction in the performance of the ITO-based FPSCs. This is primarily brought about by defects like loss of flexibility and current leakage, which are enhanced by surface imperfections in the ITO. The lower surface roughness of ITO films on PET substrates compared to glass yields some advantage but still leads to mechanical failure when flexed [28]. To work in practical usage, FPSCs need to withstand numerous physical deformations such as bending, twisting, or stretching without losing much in efficiency. Figure 6f provides the SU-8/Ca structure before bending, where minimal visible surface deformation is observed. Contrary to this Fig. 6g carnivals the SU-8/Ca structure after bending and displays no evident fracture. This suggests that the SU-8/Ca-based electrodes are more stress-resistant mechanically, which means their greater utility over ITO-based electrodes in flexible photovoltaic devices [37]. 

Hu et al. demonstrated that FPSCs maintain 85% of the initial efficiency after 5000 bending cycles of 3 mm radius of curvature. Moreover, devices have also been designed to maintain approximately 90% of their inherent PCE for 1000 cycles when the device has a 4 mm radius of curvature, perfect for wearable electronics, foldable electronics, and curved display applications. However, despite these advances, ITO-based FPSCs remain superior in performance for devices without ITO, due to improved open-circuit voltage (V_oc_), with the ITO-based devices announcing 1.13 V compared to 1.05 V for devices without ITO [38]. 

Due to mechanical vulnerability of ITO under cyclic bending, alternative electrode materials are vigorously pursued. Zhong et al. presented stable, ultrahigh conductive carbon nanosheet films as a substitute for typical ITO electrodes. Carbon-based electrodes offer higher mechanical flexibility, better conductivity, and more stable environmental conditions, which overcome mechanical failure issues plaguing ITO-based devices [39]. Recent studies have been focusing more on the synthesis of hybrid organic-inorganic electrode materials to fabricate high-performance FPSCs where alternative tactics are used: First, metal grids on a flexible substrate offer structural stability at the possible expense of decreased efficiency from resistive losses and light scattering. Second, hybrids-perovskites are being developed to combine the high efficiency of perovskites with increased flexibility, consenting compatibility with flexible structures without significant performance loss. Third, polymer composites provide mechanical flexibility and strength in FPSCs to facilitate easy incorporation into high PCE light designs. Fourth, new encapsulation procedures seal off FPSCs from environmental factors, promoting long-term stability and performance in flexible devices [27]. In the case of tandem cell designing, perovskite layers are combined with other materials such as silicon, copper CIGS, CdTe, GaAs, ZnO, SnO, TiO_2_, InP, Ag, and Pt, offer the potential for increased efficiency by harvesting a broader spectrum of light. These methods collectively offer the optimal compromise between flexibility maximization and high efficiency to facilitate widespread implementation of FPSCs development [38]. 

#### Charge carrier layers

**Fig. 7:**
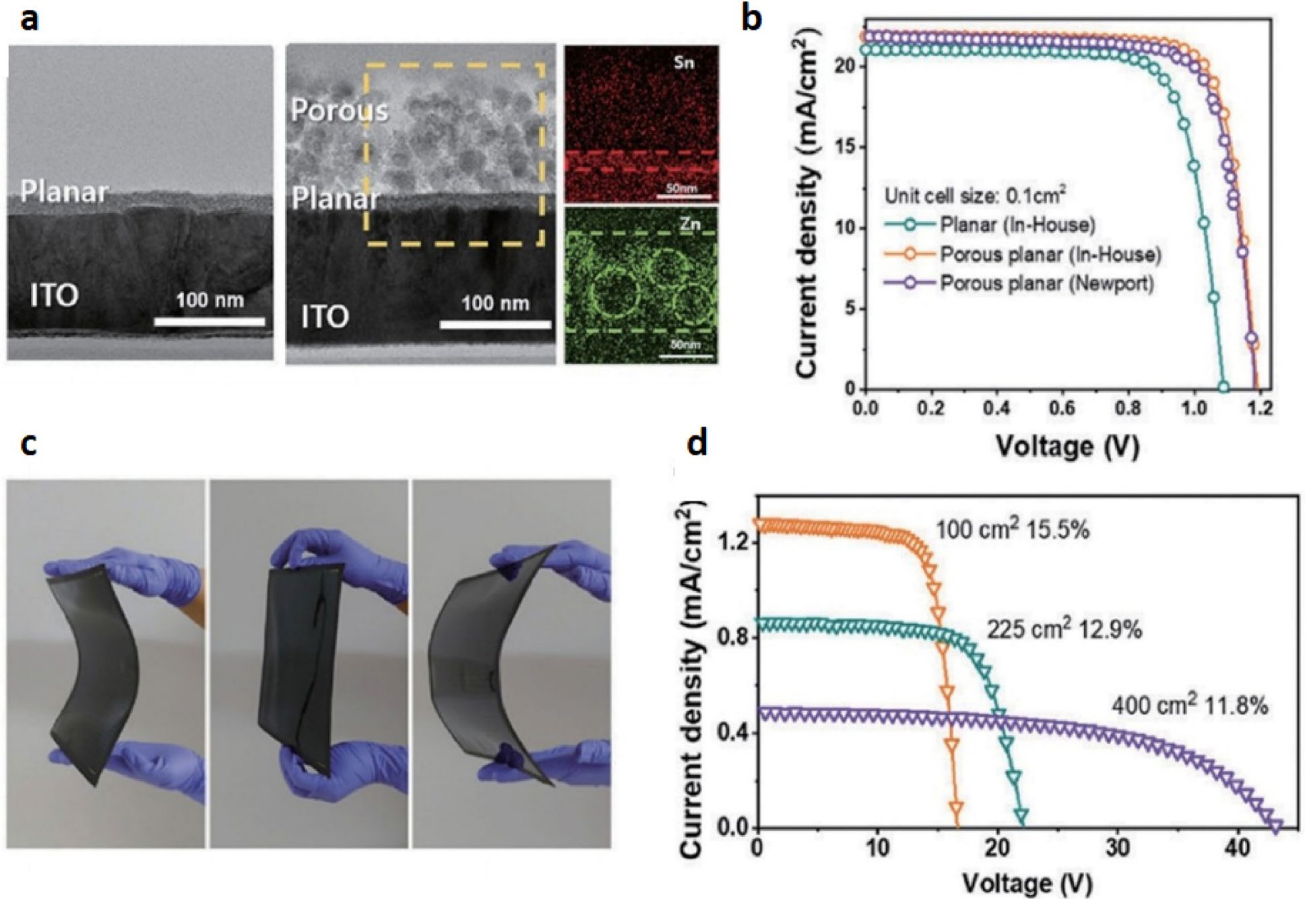
**a** TEM image shows planar, porous layer on an ITO substrate, along with EDS mapping. **b** The J–V curves of planar (green) and porous planar (orange) flexible unit cells, along with Newport certification data (purple), provide valuable insights into their performance. **c** Image of a 400 cm^2^ porous planar flexible module. **d** The J–V curves of the most optimal flexible sub-module are depicted for aperture areas of 100, 225, and 400 cm^2^. Reproduced with permission from reference [42]. Copyright 2020 The Royal Society of Chemistry

The charge transport layer (CTL) of a functioning FPSC is of very significant importance when it comes to charging carrier transporting efficiently from the perovskite absorbing layer to the proper electrodes. Figure 7 the effective demonstration of porous ITO layer flexible PSC, where Fig. 7a corresponding to cross-sectional scanning electron micrograph of the planar and porous planar ITO structure shows the morphological contrasts, which are crucial in the charge extraction process. Figure 7b presents the current–voltage (I–V) plots, showing how CTL material choice influences the overall device performance [40]. The selection and fabrication of CTL materials are influenced by several significant parameters, including the band structure, morphology of the film, and mobility of charge carriers, which all directly impact the stability and performance of FPSCs. The thermal limitation tolerance of the flexible substrate imposes limitations on the processing temperature of the subsequent layers, i.e., CTLs. In n-i-p FPSCs, inorganic ETLs such as ZnO, SnO_2_, and TiO_2_ are often utilized due to their ability to be integrated in low-temperature processing, as shown in Fig. 7c [41]. Among the various fabrication techniques described for ETLs, one of the most promising techniques is deposition of ETLs from pre-formed high-crystallinity nanoparticles or nanocrystals. However, most of the nanoparticles must be sintered at high temperatures, which is undesirable in flexible substrates. Figure 7d indicates how SnO_2_ nanoparticle dispersion has emerged as one of the most successful ETLs with potential for R2R processing and easy transfer onto flexible substrates [42]. 

Apart from these inorganic materials, other low-temperature ETLs such as C60, metal-organic frameworks (MOFs), and ionic liquids are also being extensively investigated. For p-i-n structure FPSCs, hole transport layers (HTLs) such as PEDOT: PSS, PTAA (poly[bis(4-phenyl)(2,4,6-trimethylphenyl)amine]), and NiOx are commonly used [43]. PEDOT: PSS and PTAA can be processed efficiently at low temperatures. NiOx can, however, be deposited successfully on flexible substrates by utilizing pre-synthesized nanoparticles or nanocrystals. Due to inherent deformation of the flexible substrates during processing, as-deposited CTLs are prone to have flaws such as holes, which would require further modification to ensure the structural integrity and functionality of the device [44]. 

#### High quality perovskite absorber

**Fig. 8:**
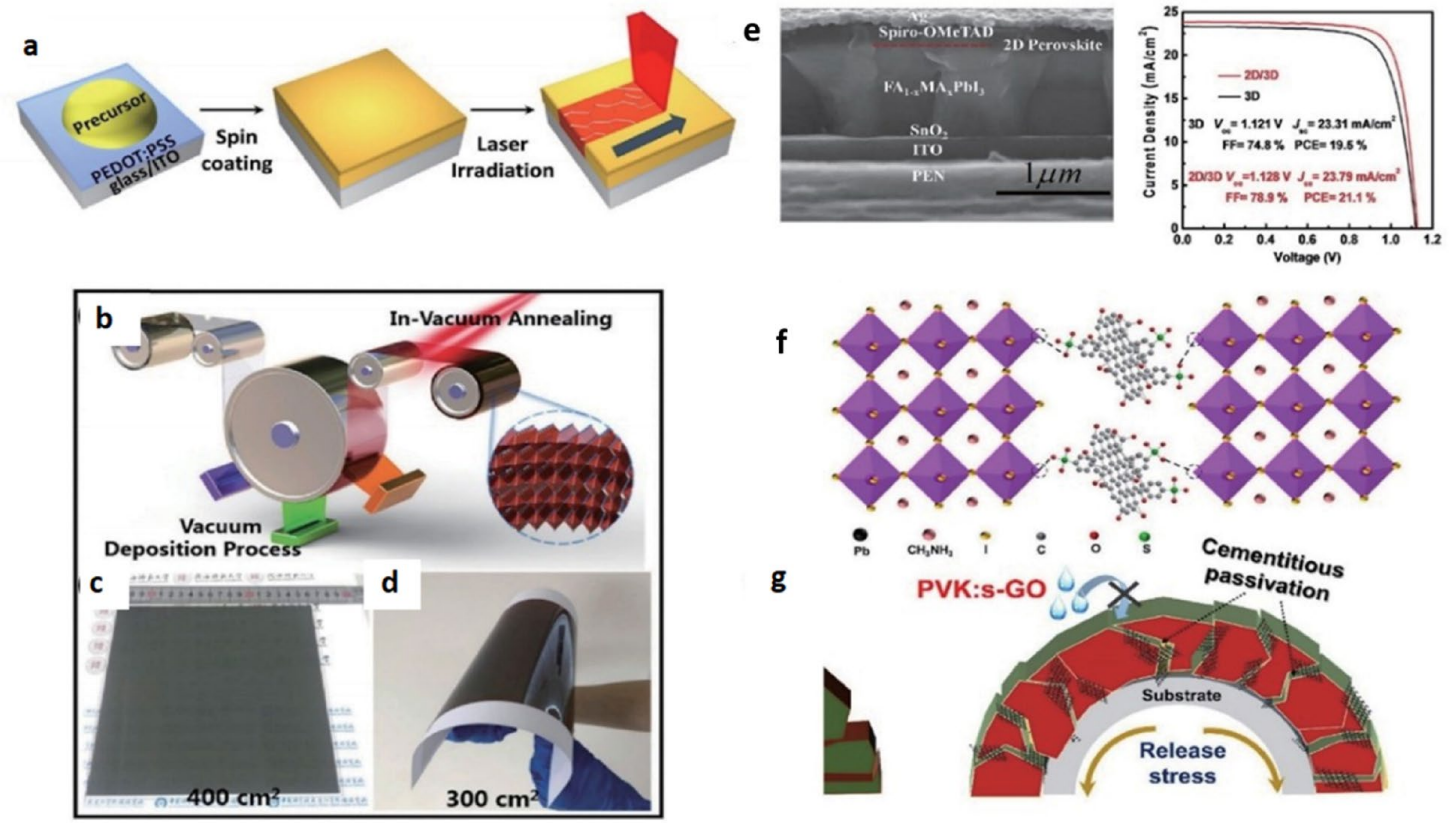
**a** Laser annealing procedure for creating perovskite films. Reproduced with permission from ref [46]. Copyright 2016 American Chemical Society. **b** Large-area perovskite films created using multisource vacuum deposition. Images of perovskite films FA on **c** glass and **d** PET substrates: Reproduced with permission from ref [50]. Copyright 2021 The Royal Society of Chemistry. **e** The J–V curves of 3D and 2D/3D FPSCs, along with cross-section SEM images, showcase the structure of an FPSC consisting of PEN/ITO/SnO_2_/3D/2D perovskite/Spiro-OMeTAD/Ag. Reproduced with permission from ref [47]. Copyright 2021 The Royal Society of Chemistry. **f** S-Graphene oxide (GO) and perovskite interaction. **g** Improved flexural endurance and water resistance attained by cementing and passivating grain boundaries. Reproduced with permission from ref [48]. Copyright 2021 Elsevier

The process techniques and materials used for perovskite films on flexible substrates are rather distinct from rigid substrates due to the distinct nature of the materials. Laser annealing is a very efficient method for room-temperature crystallization of perovskite films at a high rate. As indicated in Fig. 8a Jeon et al. initiated the laser-induced heating from a 1064 nm wavelength to heat activate perovskite layers through light absorption at the ITO and PEDOT: PSS interfaces [45]. As indicated in another study as shown in Fig. 8b–d. You et al. utilized a visible-wavelength laser to cause photothermal excitation inside the perovskite layer. This resulted in better crystal growth, i.e., increased grain size and decreased defective density. Besides, vacuum-based deposition techniques have also been utilized to deposit perovskite films onto flexible substrates successfully. Feng et al. reported a protocol using vacuum deposition and annealing at 60 °C for the preparation of rigid FA-based FPSCs [46]. As shown in Fig. 8e. dimensional composition engineering is a promising solution to achieve high-efficiency FPSCs. Yang et al. have recently achieved high FPSC efficiency through the addition of artemisinin as a perovskite grain surface passivation agent [47]. To further strengthen mechanical stability, scientists have also conducted studies aimed at improving perovskite grain boundaries by adding functional additives. Figure 8f and g for instance, illustrate grain boundaries improved using a cement-like reinforcement strategy. Hu et al. were able to dope the sulfonated graphene oxide into the grain boundaries, which resulted in enhanced mechanical flexibility and outstanding photovoltaic performance. All processes from laser annealing to grain boundary passivation contribute to the overall efficiency and lifespan of perovskite-based photovoltaics [48, 49]. 

#### Transparent electrodes

**Fig. 9:**
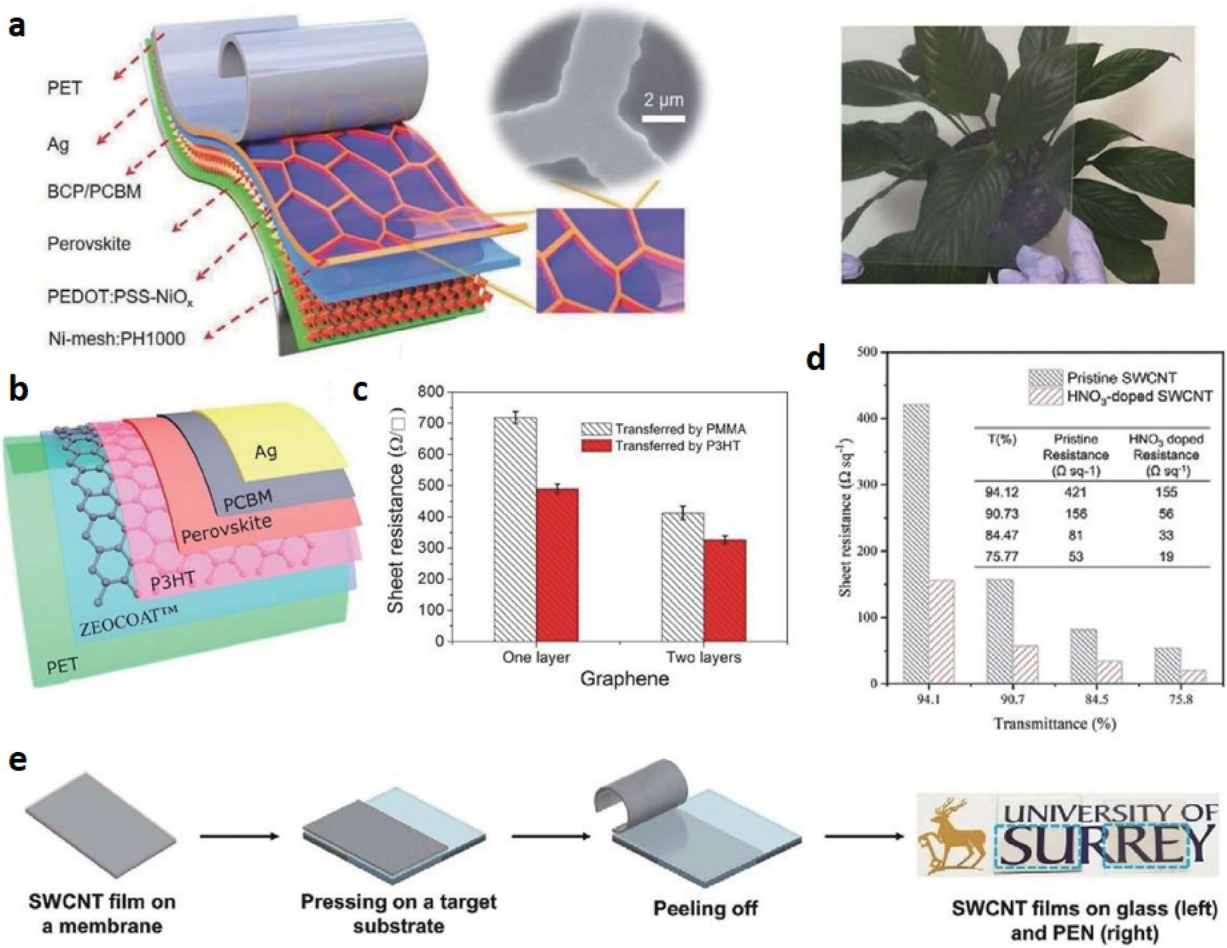
**a** The experimental setup consisted of a perovskite device, a hybrid electrode composed of Ni-mesh and PH1000, and a PET/Ni-mesh substrate, the top right image in the study showcases the SEM image of the Ni-mesh. The optical picture of the PET/Ni-mesh substrate is shown on the right. Reproduced with permission from reference [51]. Copyright 2020 John Wiley & Sons, Inc. **b** The flexible PSC was composed of PET, graphene, perovskite, phenylC70-butyric acid methyl ester (PCBM), and silver. **c** The sheet resistances of CVD graphene’s first and second layers were transferred utilizing P3HT, or poly (methyl methacrylate) Reproduced with permission from reference [52]. Copyright 2016 Elsevier. **d** The SWCNT films’ sheet resistances before and after being treated with HNO_3_ and films with various optical transmittance values. **e** On glass and PEN substrates, a SWCNT film and transferred SWCNT were dry transferred. Reproduced with permission from reference [53]. Copyright 2021 John Wiley & Sons

Indium tin oxide (ITO) is the most common transparent electrode due to its low electrical resistance and high optical transparency. However, it has the significant drawback of being very expensive to produce, with noble metals contributing significantly to the cost. Hence, other transparent conductive oxide electrodes such as aluminum-doped zinc oxide (AZO) have been studied for use in FPSCs. Despite the advantages, the conductive oxide electrodes are brittle and limit their application in harsh environments, for instance, in stretchable and deformable devices. Metal nanowire mesh solution-processable electrodes have also demonstrated significant potential as transparent electrodes due to their high conductivity, transparency, and flexibility [51]. Further, their solution process ability enables their R2R mass production, making them suitable for production on a large scale. Silver (Ag), copper (Cu), and nickel (Ni) nanowires and meshes have been used massively as electrodes in FPSCs. Recent work by Li et al. demonstrates an FPSC with nickel mesh as an electrode with low sheet resistance and high optical transmittance (Fig. 9a). The bending flexible device performed still maintained a good percentage of its initial performance achieve in 2000 bending cycles. However, due to the natural roughness and reactivity of metal nanowires with perovskite materials, surface modification is usually required. To address this challenge, substances such as ITO, AZO, GO, graphene, and PEDOT: PSS have been coated onto the metal nanowire surface to optimize performance [52]. 

Carbon-based materials, including graphene and carbon nanotubes, have also been researched as transparent electrodes for FPSCs owing to their high optical transmittance and conductivity. Graphene was first applied by Liu et al. as an FPSC electrode material, demonstrating significant promise for flexible solar cell applications (Fig. 9b, c). Jeon et al. compared the performance of FPSCs using graphene and single-walled carbon nanotubes (SWNTs). The increased morphology and transparency of graphene resulted in improved device performance, while the SWNT-based FPSCs exhibited enhanced mechanical flexibility, attributed to the random orientation of the SWNTs. Zhang et al. recently employed SWNTs as electrodes by utilizing simple dry transfer technology to fabricate FPSCs with superior performance (Fig. 9d, e). PEDOT: PSS is also an extremely ubiquitous conductive polymer material used in flexible and stretchable electronics. It is possible to achieve high conductivity in as-cast PEDOT: PSS films with values of about 4000 S/cm through various doping and coating methods. This makes PEDOT: PSS a promising alternative to ITO electrodes in solar cells. Conductive PEDOT: PSS was used by Hu et al. to make FPSCs with significant performance improvements. Although PEDOT: PSS is a very good replacement for ITO, its acidity can lead to perovskite material corrosion, which could compromise the stability of the devices in the long term [53]. 

### Schematics for equivalent circuits and perovskite solar cell

**Fig. 10:**
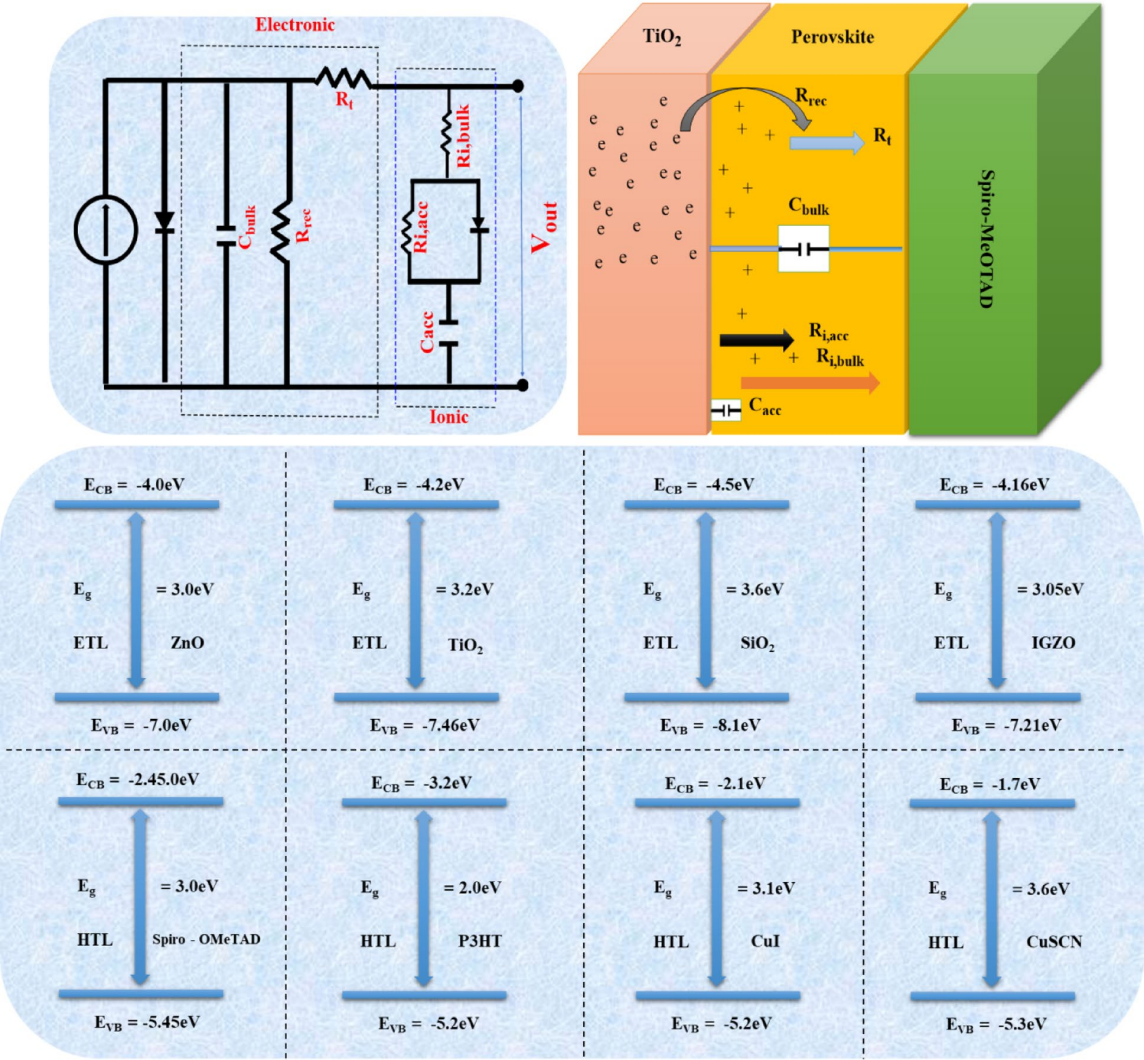
**a** Equivalent circuit and **b** perovskite solar cell. **c** Energy level diagram shows multiple layers containing HTLs and ETLs

Ebadi et al. proposed a PSC equivalent circuit schematic, incorporating elements like R_rec_ for electron recombination resistance and C_bulk_ for perovskite bulk capacitance. The circuit accounts for various modes of charge storage and the electronic contributions of photo-generated carriers. R_ibulk_ represents ionic transport resistance, while C_acc_ and R_i,_acc pertain to capacitance and interface resistivity, respectively. The circuit’s diode component reflects asymmetrical charge dynamics, influencing charge collection efficiency and bulk scattering effects in solar cells, as shown in Fig. 10a and b [54]. 

Poisson equation,

$$\begin{aligned}\frac{\partial^{2}\uppsi}{\partial^{2}\text{x}}&=-\frac{\partial \text{E}}{\partial \text{X}}=-\frac{\uprho}{\upvarepsilon_{\text{s}}}\\ &=-\frac{\text{q}}{\upvarepsilon_{\text{s}}}[\hbox{p}-\hbox{n}+\hbox{N}_{\text{D}}(\hbox{x})\pm\hbox{N}_{\text{def}}(\hbox{x})]\end{aligned}$$

The equation establishes a relationship between the space charge density and the electric field (E) of the p–n junction. Where Ψ = electrostatic potential. q = elementary charge (1.6 × 10^−19^ C). ε_s_ = static material permittivity. p represents the density of holes, while n represents the density of electrons. The ionized donor and acceptor densities are N_D_ and N_A_, respectively.

The carrier continuity equations at steady state describe the behavior of electrons and holes,

$$\begin{aligned}&\frac{\partial\text{J}\,_{\text{n}}}{\partial\,\text{x}}+\hbox{G}-\hbox{U}(\hbox{x},\,\hbox{p})=0\\ &-\frac{\partial\text{J}\,_{\text{n}}}{\partial\,\text{x}}+\hbox{G}-\hbox{U}(\hbox{x},\,\hbox{p})=0.\end{aligned}$$

In the below equations, J_n_ = current density of electron carriers and J_p_ = current density of hole carriers, while Un and Up denote the net recombination rates. G represents the generation rate of electron-hole pairs.

Drift diffusion equations,

$$\begin{aligned}\hbox{Jn}&=\hbox{qp}\upmu_{\text{n}}\hbox{E}+\hbox{qD}_{\text{n}}\frac{\partial\text{n}}{\partial\text{X}}\\ \hbox{Jp}&=\hbox{qp}\upmu_{\text{p}}\hbox{E}+\hbox{qD}_{\text{n}}\frac{\partial\text{n}}{\partial\text{X}}\end{aligned}$$

µ_n_ and µ_p_ represent the mobility of electrons and holes. The symbol q represents the elementary charge, while Dn and Dp denote the diffusion coefficients of electrons and holes [55]. 

#### Roles of carrier’s transporter in solar cells

In PSC structures, the active layer is carefully placed between two extremely thin functional layers for effective charge transport, namely, HTL and the electron transport layer ETL. Charge separation and extraction are greatly influenced by energy level alignment, i.e., electron affinity and ionization potential. Materials with shallow ionization potentials and low electron affinities are typically used to provide hole transport, while higher electron affinities and deeper ionization potentials make good electron conduction channels, as shown in Fig. 10c [56]. 

#### Transport-hole layer

**Fig. 11:**
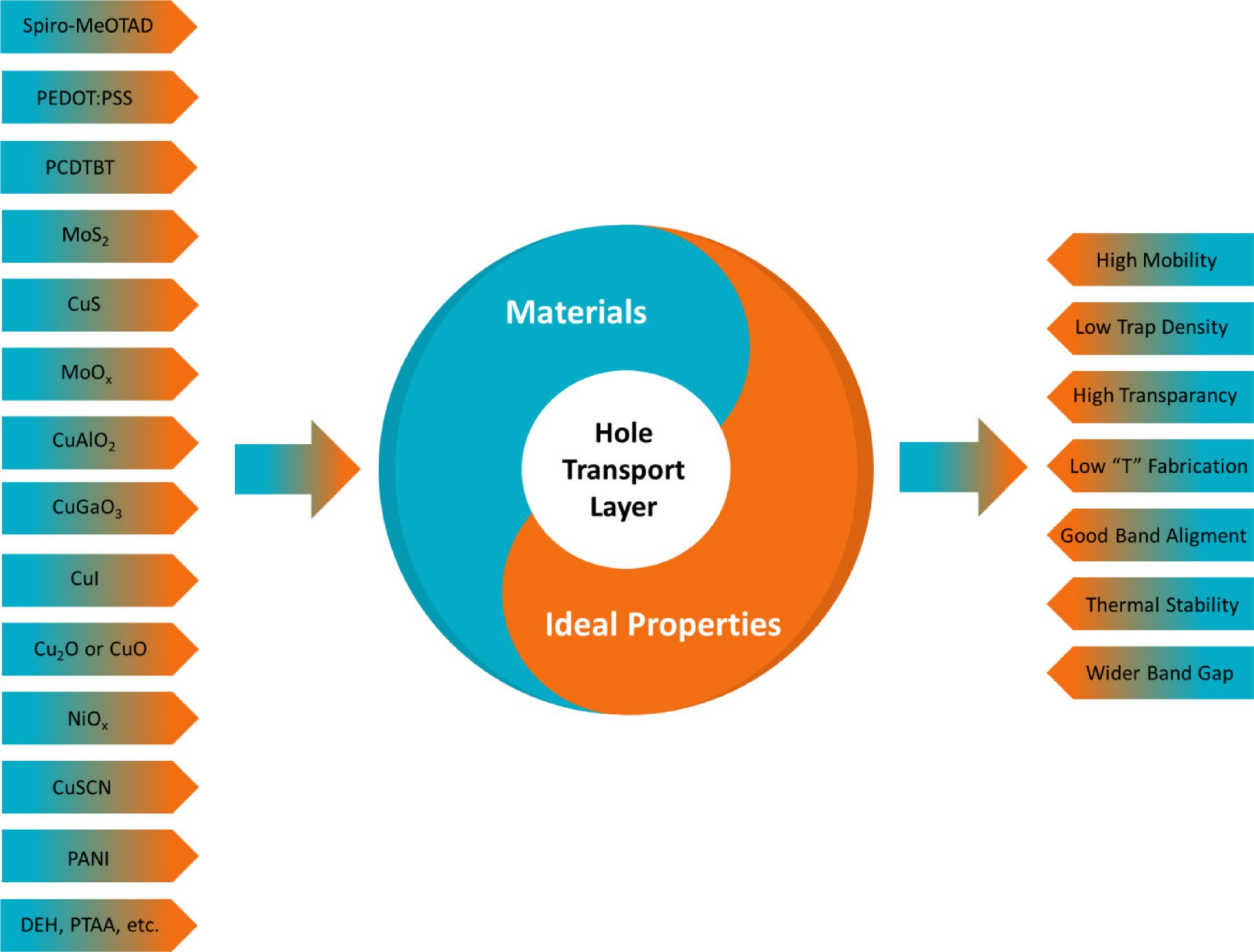
Hole transport material and its ideal properties

In FPSCs, hole transport materials with inhibited electron mobility are purposely selected to consent efficient hole transport. HTL plays a significant role by effectively extracting holes from the perovskite absorber and guiding them to the back electrode. The layer not only transports holes but also acts as a selective barrier to prevent electron flow. Figure 11 illustrated prevalent HTL materials include Spiro-MeOTAD, PEDOT: PSS, PCDTBT, MoS_2_, CuS, MoO_x_, CuAlO_2_, CuGaO_3_, CuI, Cu_2_O or CuO, NiOx, CuSCN, PANI, DEH, and PTAA, have special advantages in terms of enhancing the competence of FPSC. Desirable characteristics of the materials are high mobility for efficient transport of charges, low trap density to reduce the recombination losses, high transparency for maximum absorption of light can occur, and low-temperature fabrication ability which is crucial for flexible substrates. In addition, effective band alignment permits effective charge extraction, thermal stability for reliability over a variety of conditions, and increased band gaps reduce recombination loss, that upgrade the overall aptitude and lifetime of the devices [57–59]. 

#### Transport-electron layer

**Fig. 12:**
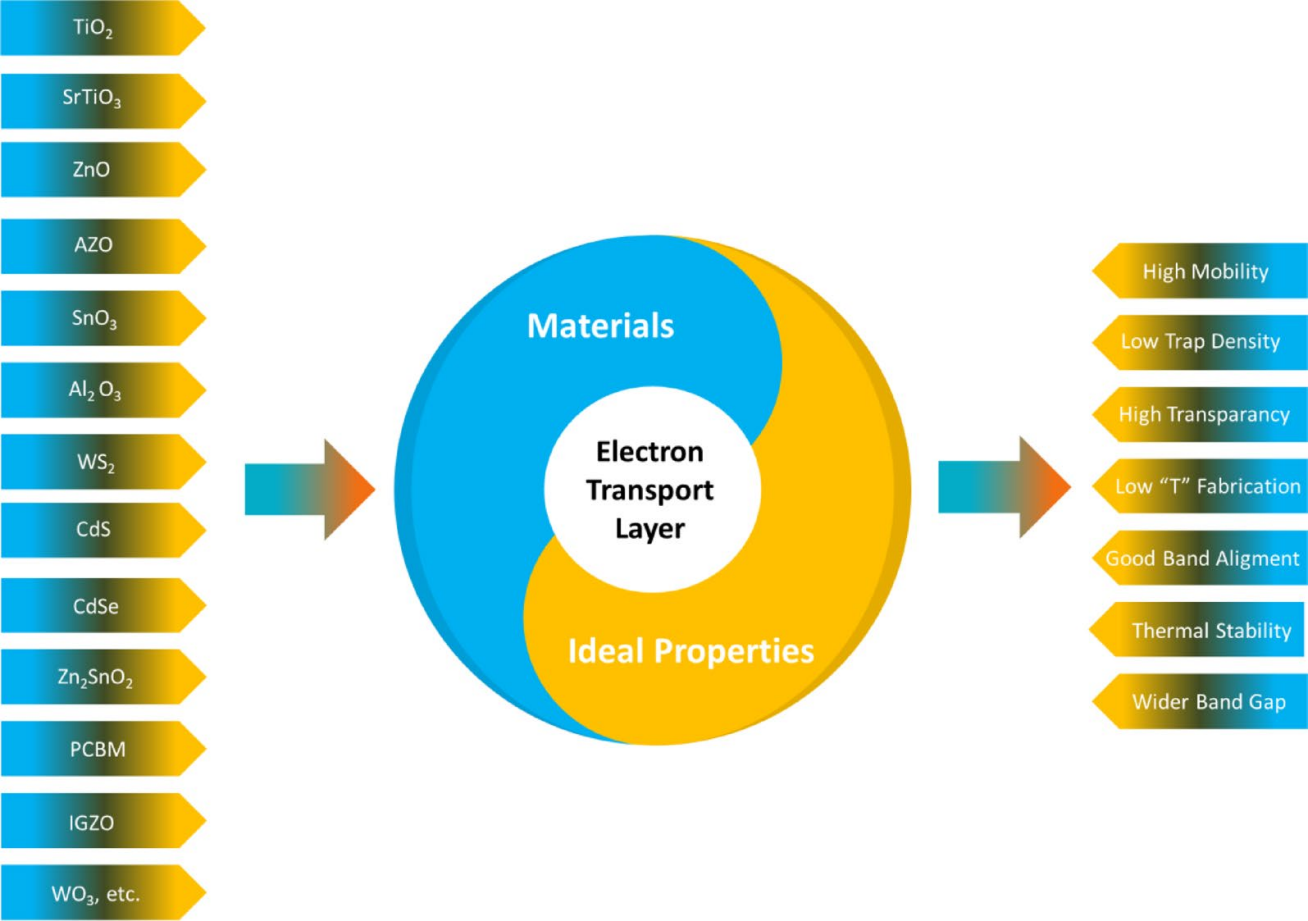
Electron transport material and its ideal properties

In FPSCs, an electron transport layer ETL is employed to enable the efficient passage of electrons from the perovskite absorber to the front electrode. It also acts as a selective barrier that blocks holes, thus inhibiting charge recombination at the interface (Fig. 12) describes some of the ETL materials that are common in photovoltaic devices, including TiO_2_, SrTiO_3_, ZnO, AZO, SnO_3_, Al_2_O_3_, WS_2_, CdS, CdSe, Zn_2_SnO_3_, PCBM, IGZO, and WO_3−x_. These materials have assets related to high mobility, low trap density, high transparency, low-temperature processing, good band alignment, thermal stability, and improved band gap. The properties result in better performance and stability of the electronic and optoelectronic devices, which is especially factual with FSC devices [60–62]. 

#### The electrodes for solar cell device structure

**Fig. 13:**
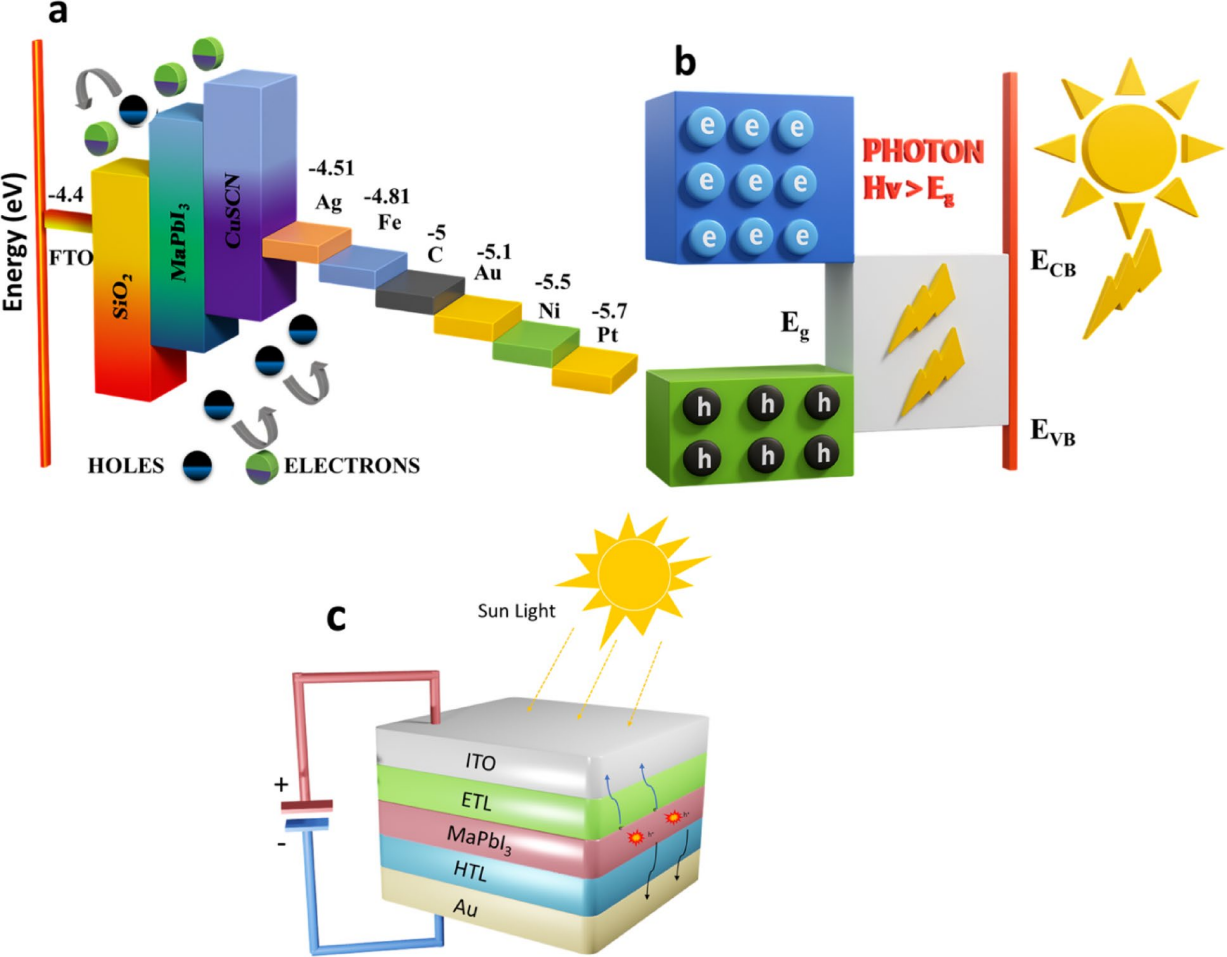
**a** Detailed values of the various anodes and cathodes. **b** Photons with high energy (E_V_ >E_g_) are absorbed. **c** Formation of excitons and carrier transport modes because of photon absorption

The anode is an important electrode that assists the photovoltaic (PV) elements in holes and electrons generation plus collection. Anode is an upper electrode almost transparent, having the lowest reflectance, highly conductive, proper work function, and easy to pattern. When the device is on, the anode electrically injects holes while simultaneously drawing out the electrons from inside the device [63]. On the contrary, the cathode is responsible for collecting holes generated in the absorber layer. Depending on the design of the FPSC, the cathode may be transparent or opaque. Figure 13a illustrates the comparison between different anode and cathode materials along with their work functions and working mechanism of the PSC. Basically, solar cells serve as the heart of any PV system, converting electrical energy from sunlight via photovoltaic effect in semiconductor layers. Each layer needs to be optimized to make sure it is extremely efficient in PCE for top performance. When solar radiation falls on a cell, the solar cell starts absorbing photons with energy more than or equal to the bandgap of the semiconductor (E_g_), initiating charge generation [64]. 

As shown in Fig. 13b high-energy photons absorbed by perovskite materials cause electrons to be excited to the conduction band, leaving behind holes in the valence band where current generation is triggered. In these materials, excitons (electron-hole pairs bound) can either be intact or dissociate into free carriers, both resulting in device operation. Because of the internal electric field, the electron-hole pairs that are created become separated, with the electrons going in the n-type and the holes in the p-type direction. Separation reduces recombination losses as the carriers travel through the external circuit and deliver power to an external load. The HTL serves to collect holes efficiently, while ETL, in combination with a metal electrode and FTO, facilitates electron extraction [65]. The p–n junction’s built-in electrostatic field aids in charge separation and resists the energy barrier. Photons incident with energy greater than the bandgap (E_g_) can excite electrons across the bandgap, initiating carrier migration. The bandgap (E_g_) is defined as the energy difference between the conduction band minimum (E_c_) and valence band maximum (Ev):

$$\hbox{E}_{\text{g}}=\hbox{E}_{\text{c}}-\hbox{E}_{\text{v}}$$

After excitation, electrons move through the conduction band, and holes move in the opposite direction through the valence band. The charge carriers reach the depletion region without recombining, and the accumulated charges create an internal electric field that opposes the initial potential barrier, as illustrated in Fig. 13c [66]. 

#### Exciton generation rate versus photon absorption

In FPSCs, a high fraction of incoming light is absorbed before it reaches carrier transport layers, reducing the number of photons that can be processed efficiently. For compensation, a CTL-free design would enhance absorption of light and exciton generation, ultimately leading to a more efficient device. The limitation in FPSCs is one where there is improper carrier generation in the active layer, which impacts overall performance adversely [67]. We can solve the problem of limited carrier generation for FPSCs by methods including compositional optimization of the perovskite layer, light management strategies, and bandgap engineering. We could also consider interface engineering to improve charge extraction and passivation of defects that could reduce recombination losses, hence improving carrier generation. Possible further development includes implementing 2D/3D hybrid structures and optimizing the thickness of an active layer in perovskite solar cells. Overall, these strategies could provide better carrier generation and improved efficiency for the devices [68]. 

#### Enhanced carrier recombination

Carrier recombination from the mutual annihilation of holes and electrons in perovskite materials is among the key challenges in achieving high efficiency in FPSCs. The mechanism prevents efficient charge carrier extraction at the electrodes. Furthermore, very thin perovskite films may not be able to absorb sufficient light and will lead to low photon generation and current density. On the other hand, excessively thick perovskite layers can increase recombination losses and limit device performance [31]. These issues can be addressed by tailoring the thickness of the perovskite layer, as well as by using defect passivation processes to enable greater external quantum efficiency. Two charge transport layers can also be employed to facilitate improved charge separation and transport, leading to overall better device performance. The key solutions to counteract increased carrier recombination include maximum perovskite layer thickness, applying defect passivation measures, and employing double charge transport layers to maximize charge transport and separation [69]. 

#### Lower performance

One of the major problems during the synthesis of FPSCs is that they perform poorly, due to the use of relatively large-bandgap and unwell aligned energy levels in the layers. These factors contribute to low current density and low electrical output, hindering device efficiency. For resolving this issue, bandgap tuning of the material in PSCs is important, which will enhance better energy level matching among the perovskite layer and charge transport layers. The decrease in light absorption losses and increase in overall material quality with the assistance of better fabrication processes is very crucial to increase the current density as well as the electric output. These measures improve the performance of FPSCs and make it more resourceful [70, 71]. 

#### Stability of device

FPSCs stability is highly dependent on environmental factors of heat and humidity, even with encapsulation. Additionally, achieving a balance between long-term stability and the thin, light, and flexible characteristics of the devices remains challenging. To enhance the operation stability and overall performance of PSCs in the long term, one of the possible steps is replacing lead (Pb) with more chemically stable materials like tin (Sn) and germanium (Ge) in the perovskite material. This replacement would increase the resistance of the device to environmental degradation and hence extend its life and overall stability while maintaining the desired flexibility and efficiency [72, 73]. 

## Different configurations of perovskite solar cells

Recently there have been advances in FPSC, such as mesoporous (n-i-p), and planar (n-i-p and p-i-n) structures. In n-i-p architectures the transparent conductive electrode is above ETL which allows light to penetrate through the cell. On the other hand, the p-i-n architecture represents an inverted structure where HTL is before the perovskite layer. Overall, the development of various architectural designs facilitates changes in PSC improving efficiencies and subsequently evolving technologies [74, 75]. 

### Perovskite solar cell (n-i-p) with regular structure

**Fig. 14:**
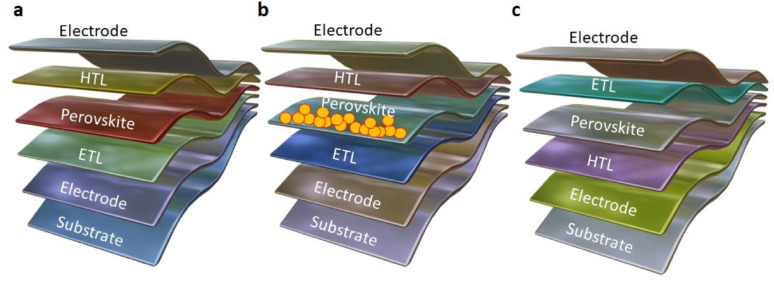
Illustrated cell architectures of perovskite devices include: **a** Regular n-i-p planar structure, **b** Mesoporous n-i-p framework, and **c** Inverted planar p-i-n configuration

Planar perovskite solar cells (PPSCs) receive substantial interest because they provide low-temperature manufacturing methods and cost-effective production with simple assembly processes. The planar n-i-p design differs from mesoporous structures because it contains an unbroken ETL which defines its structural organization. This design contains two vital junctions between the ETL and perovskite and perovskite and HTL which enable quick electron-hole pair separation exhibited on Fig. 14a. The coordinated interaction between the HTL and ETL supports effective exciton dissociation, contributing to improved device performance [76, 77]. 

### Solar cell with mesoporous perovskite (n-i-p)

Mesoporous perovskite solar cells (MPSCs) are the new promises due to their low price, easy fabrication method, and high PCE. MPSCs typically contain a fluorine-doped tin oxide (FTO) layer, which prevents hole transport but allows electron extraction prior to the compact layer. One of the defining features of MPSCs is the presence of nanostructured pores within the ETL, which renders them distinct among the photovoltaic community, as evident in Fig. 14b. Adding a mesoporous scaffold is designed to enhance efficiency and working stability of the device. Scalability and performance under real conditions are still a concern. Various metal oxides such as TiO_2_, Al_2_O_3_, ZnO, SnO_2_, and ZrO_2_ are typically employed to increase light absorption and improve the efficiency of these solar cells [78, 79]. 

### Perovskite solar cell with inverted planarity (p-i-n)

Inverted FPSCs with p-i-in architecture have proven to be exciting, efficient and versatile photovoltaic devices due to the sequential advantages such as easy to fabricate, high operating stability, and compact hysteresis. Inverted FPSCs also have a higher level of intrinsic stability due to the unique charge transport layer configuration. Inverted devices have a p-i-n configuration, and in inverse order of the conventional n-i-p structure; that are referred to as an inverted planar structure. A thin HTL is added to an inverted device to improve hole extraction and ultimately device stability as shown in Fig. 14c. The functioning of each layer in planar FPSCs is critical for achieving higher efficiency of the device. Once the performance of the inverted solar cell has been measured, every measure is being taken to modify the structure, introduce new perovskite compositions and apply sophisticated passivation processes to achieve better inverted perovskite solar cells [80, 81]. 

## Classification of flexible substrates

Substrates employed in the fabrication of FPSCs are required to possess some essential properties, including excellent thermal stability, mechanical flexibility, high electrical conductivity, and good optical transparency. Various types of flexible substrates, including plastic, metal, and ceramic materials, have been employed in the construction of FPSCs. The following section shall compare, in more detail, the advantages, limitations, and potential applications of each substrate, providing an overall view of how they are working to advance FPSC technology [82]. 

### Substrate of metal

**Fig. 15:**
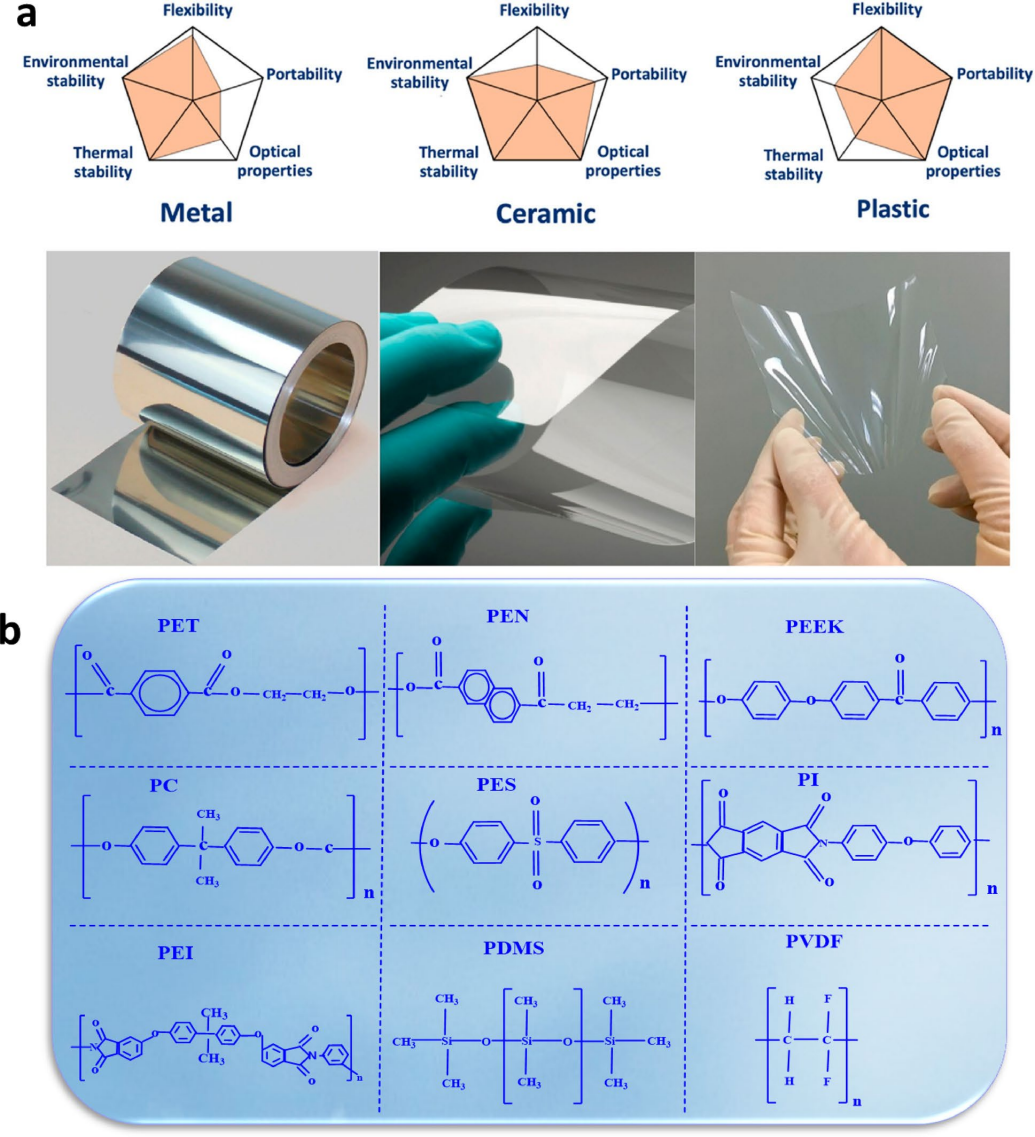
**a** Presented is a qualitative comparison of five fundamental attributes that define substrate materials made from metal, ceramic, and plastic. At the bottom, images showcase stainless steel to the left, Corning’s flexible willow glass in the center, and colorless polyimide film to the right. Reproduced with permission from ref [87]. Copyright 2021 Elsevier. **b** Accompanying this is a breakdown of the molecular compositions of these substrates

Figure 15a show metal foils with a typical thickness of around 125 μm are typically employed as flexible substrates for FPSCs due to their high flexibility, ductility, and mechanical strength. Among them, stainless steel is particularly favorable because it is cheap, possesses high thermal resistance, and chemical stability. The first FPSCs, developed in the 1980s, used hydrogenated amorphous silicon (a-Si: H) and cadmium sulfide (CdS) as active layers. Stainless steel has recently been utilized as a substrate for copper indium gallium selenide (CIGS) solar modules. Other metals such as titanium foils and aluminum alloys have also been considered as substrates. Although titanium foil has demonstrated 13.07% PCE, its relatively high cost doesn’t permit mass production [83]. 

### Substrate of ceramic

Glass is perhaps the most ubiquitous ceramic substrate for solar cell technology due to its exceptional thermal stability and resistance to chemical degradation and water absorption. Recently, due to advancements in glass-manufacturing processes, excess glass variants, such as Corning Willow Glass, have hit the market. Glass substrates previously fragile were also useful starting in 2013, from which time researchers have used flexible or otherwise bendable glass substrates in photovoltaic designs. In 2017, a significant achievement manifested with a flexible perovskite solar cell made and tested on glass that had a maximum PCE of 18.1%. As in Fig. 15a other ceramic materials, like ribbons of zirconia, were also focused on for some designs and used with glass [84, 85]. 

### Substrate of plastic

Plastic substrates like polyethylene naphthalate (PEN) in Fig. 15a are commonly favored for FPSCs due to their lightweight and cost-effectiveness. Despite achieving a high PCE of up to 19.1% on PEN substrates, vulnerability to oxygen and moisture poses challenges. Barrier layers like silicon oxide and aluminum oxide can alleviate this issue. However, plastic substrates have restricted heat stability, making them unsuitable for high-temperature deposition processes required by some solar cell types. They surpass at fabricating solar cells at low process temperatures, making them well-suited for organic/polymer solar cells and perovskite solar cells [86]. 

### Plastic substrates classification

Various plastic substrates are often used in the fabrication of flexible solar cells. These include polyethylene terephthalate (PET), polyethylene naphthalate (PEN), polycarbonate (PC), polyether sulfones (PES), polyimide (PI), polyether ether ketone (PEEK), polyamide-imide (PAI), polyetherimide (PEI), polydimethylsiloxane (PDMS) and polyvinylidene fluoride (PVDF) as demonstrated in Fig. 15b. Further details on each of these materials are provided in the following sections [88, 89]. 

#### Substrates of PET and PEN

Semi-crystalline thermoplastic polyesters, polyethylene terephthalate (PET) and polyethylene naphthalate (PEN), have proven to be valuable flexible materials for commercial use as substrates in PV. While PET is formed by the esterification of ethylene glycol, and terephthalic acid, PEN is prepared from the reaction of ethylene glycol, and naphthalene dicarboxylic acid to form ethylene naphthalate, etc. These polymers provide one of the most robust industrial histories, like metal and glass, and can easily fit into existing PV manufacturing facilities. Part of the reason for their popularity is due to their properties which include highly ductile (up to 70% elongation at break) as well as high optical clarity (previously mentioned average about >85% visible range transmittance) and mechanical robustness to bending or buckling stresses because of low Young’s modulus [90]. PET and PEN both possess inherent hydrophobicity and high tolerance to solvents and chemicals. Moreover, their enhanced dielectric properties enable electronic use. Water uptake is extremely low- approximately 0.14%- although their water-blocking grades differ with PET of 100 μm thickness demonstrating a water vapor transmission rate of 9 g/m^2^/day, whereas PEN considerably lower rating is at 2 g/m^2^/day. Both PET and PEN exhibit good oxygen and moisture barriers but cannot meet the demanding environmental barrier conditions of flexible solar cells and require barrier coatings. Both PET and PEN thermal properties showed a glass transition temperature (T_g_) of 78 °C and 120 °C respectively, while both started to deform around temperatures above 200 °C. Although PEN is typically smoother and performs better in some areas, it is generally more expensive. No material is resistant to degradation after prolonged UV exposure. To enhance the strength and usability properties of the substrates in PV applications, low-cost and readily applicable barrier layer technologies continue to be developed [91]. 

#### PEEK substrate

Transparent polyketone materials, or polyether ether ketones (PEEK) are in the family of high-performance thermoplastics that are known for their moisture resistant, wear-resistant, and strength. PEEK is one of the most advanced semi-crystalline polymers available. PEEK has a reasonable amount of flexibility with a Young’s modulus of approximately 3.6 GPa and tensile strength in the 140–170 MPa range. In addition to flexibility and strength much of PEEK usefulness can be attributed to its extremely high thermal resistance, being stable mechanically as high as 350 °C due to its unique chemical backbone. Still further, PEEK exhibits exceptional biocompatibility with mechanical and structural properties, making PEEK a great candidate for solar energy systems and other electronic applications [79, 92]. 

#### PC and PES substrates

Polycarbonates (PC) and polyether sulfones (PES) are amorphous, non-crystalline thermoplastics that have carbonate and diphenyl sulfone segments, respectively. Like many other polyimide and polyester polymers, these polymers are transparent, strong, rigid but lightweight materials that also possess greater impact resistance than the other plastic substrates. Their high optical clarity and very high bond energy (approx. 300 J/m^2^) and glass transition temperature (T_g_ ~ 150 °C) make them especially useful for applications like optoelectronics. As such, PC is highly utilized with mechanical utility and chemical stability features. PC has good surface and edge dimpling flexibility (flexural modulus 2.4GPa), and an excellent impact tolerance of ~ 960 J/m. As a point of reference, an untreated PC has relatively moderate adhesion intolerances, and the poor surface adhesion and wettability of PC films are also well-known. Several surface modification processes were used to address these shortcomings to the surface of PC films including air and water plasma treatment, chemical functionalization, UV/ozone treatment, and plasma enhanced CVD [93]. PES, on the other hand, has dimensional stability across a significant thermal range and has very little shrink range during molding allowing for easy fabrication. PES has a high glass transition temperature of approximately 220 °C, greater than 90% optical transparency, and compatibility with chemical agents found in semiconductor processing. Finally, PES is stable in both acidic and basic environments and retains structural integrity across a wide variety of concentrations and thermal conditions. One disadvantage is that PES substrates have normally a greater surface roughness than standard glass materials [94]. 

#### PI substrate

Imide monomers form the basis of polyimide (PI) polymers, synthesized from dicarboxylic acids and ammonia. PIs consist of two nitrogen molecules linked to organic and acyl groups, with tailorable characteristics. Kapton, a thermally resilient PI material, is used in semiconductor applications but may pose challenges for flexible electronics. The solubility of PIs in polar solvents can influence layer-by-layer processes, requiring strategies for addressing transparency and solvent tolerance trade-offs [95]. 

#### PEI substrates

Polyetherimide or PEI is a high-performance thermoplastic, which is an amorphous member of the broader family of polyimide (PI) polymers. Its molecular structure contains ether linkages, thus positioning it in close structural analogy to traditional PI polymers. Because of its high content of aromatic structures, PEI is highly resistant to radiation. This multifaceted polymer substrate is defined by its high-quality mechanical and thermal properties including a tensile modulus of about 2.9 GPa, high dielectric properties, very low hydrolysis rate of around 0.25%, and high thermal stability. It also possesses a very high glass transition temperature of about 217 °C and good biocompatibility, and thus it is a worthy candidate to replace materials like PEEK. Although PEI is partly similar to PI in terms of transparency, it is typically distinguished based on its yellowish color [96]. 

#### PDMS substrate

Polydimethylsiloxane (PDMS) is considered the simplest type of siloxane polymer, with only methyl groups attached to silicon atoms. Thus, the polymer consists of a repeating backbone [–Si (CH_3_)_2_–O–]_n_ in the chain. PDMS, as a silicone polymer, is typically clear and elastomeric; PDMS is known for its great flexibility and very low permeability to both moisture and oxygen. These materials possess excellent optical transparency, high reversible elasticity, resistance to chemicals, and the capability to form thin films that can be handled independently. Therefore, stretchable electronics applications can greatly benefit from using it as a substrate. However, PDMS has a relatively low (50 °C) glass transition temperature and a surface energy that makes it challenging to wet and causes problems with stiction with other thin films. Also, PDMS perform suboptimally at temperatures greater than 150 °C [97]. 

#### PAR substrate

The thermoplastic aromatic polyesters known as polyacrylate (PAR) have exceptional electrical, flexural, heat-resistant, UV-stable, and toughness qualities. The properties of PAR polymers can be customized by adjusting the composition, incorporating high-performance thermoplastics through alloying, and introducing reinforcement materials. Due to their remarkable versatility, engineering thermoplastics are now widely utilized in both established and emerging markets, making PAR one of the most adaptable high-temperature available polymers. Light yellow to amber-colored PARs are clear and have an optical transmittance of 87%. The glass transition temperature for PARs is 185 °C, but their chemical resistance is relatively limited [98]. 

#### PVDF substrate

**Fig. 16:**
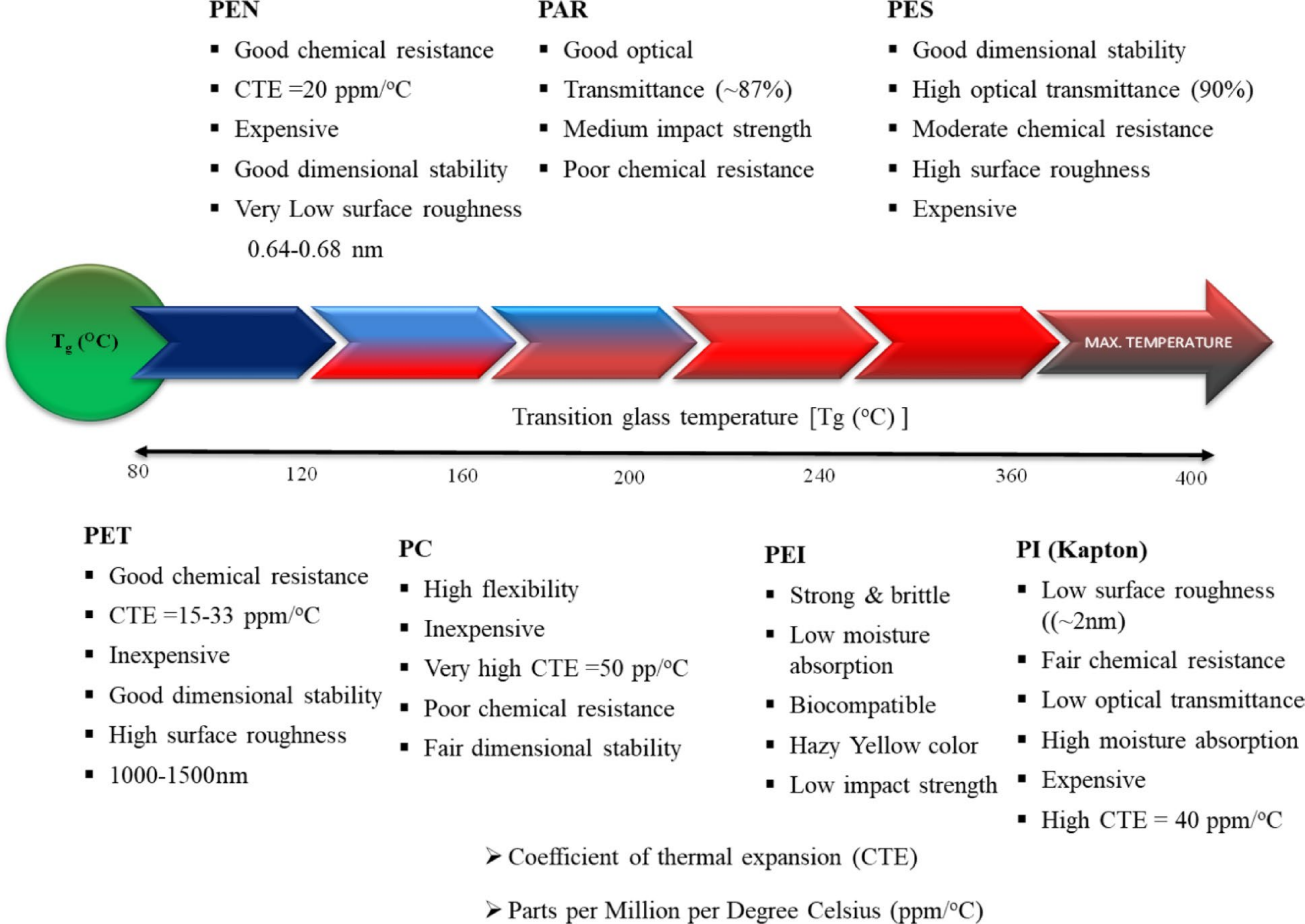
Different plastic substrates for flexible solar devices are compared

PVDF is a semi-crystalline thermoplastic fluoropolymer that was developed to provide a combination of ease of fabrication and strong mechanical properties. PVDF is one of the uncommon groups of engineering polymers; recently, it has attracted great interest in the scientific community, mainly because of its extraordinary piezoelectric behavior, which makes it the best piezoelectric polymer that is commercially available [99]. In addition to its exciting piezoelectric response, PVDF offers good abrasion resistance, excellent thermal stability (glass transition temperature of 38.3 °C), dielectric performance, good resistance to ultraviolet radiation and ionizing energy, and it absorbs little water (usually below 5%). Due to the low glass transition temperature, devices made with PVDF require fabrication at lower temperatures. Solution-based approaches are used to make solar cells on PVDF and they allow for efficient deposition processes with low processing temperatures. The temperature, PVDF-like flexible polymers will be integral to solar cell and piezoelectric technologies [87]. A temperature tolerance of polymers is shown on Fig. 16 and a comparison of some plastic substrates is presented in Table 1.

**Table 1:** Property comparisons of several plastic-based polymer substrates

Substrate	Elongation (%)	Young’s modulus (GPa)	Tensile strength (MPa)	Glass transition temperature (°C)	Optical transmittance (%)	Bandgap (eV)	Dielectric constant	Refs
PET	70	3	120–180	75	85	3.2	3-3.2	[87]
PEN	60	3.3	200	120	80–90	3.4	2.7–3.2	[100]
PEEK	20–40	3.6	140–170	350	20–50	3.5	3.2–3.5	[101]
PC	70	2.5	60–90	150	85–90	3.3	2.9–3.4	[90]
PES	20–50	2.8	70–100	220	< 90	3.1	3.2–3.5	[91].
PI	5–7	3.1	100–200	300	60–70	3–3.4	3.3–3.5	[93]
PEI	3–5	2.9	80–120	217	85–90	3.2	3.2–3.5	[94]
PDMS	430–640	0.87	3–15	50	90–95	3.2	2.5	[95]
PAR	5–20	2.3	50–150	180–185	85–87	3.5	3.4–3.8	[96]
PVDF	20–25	2.9	50–57	38.5	89–90	5.6	7–8	[99]

### Characteristics of classical substrate for FSCs

**Fig. 17:**
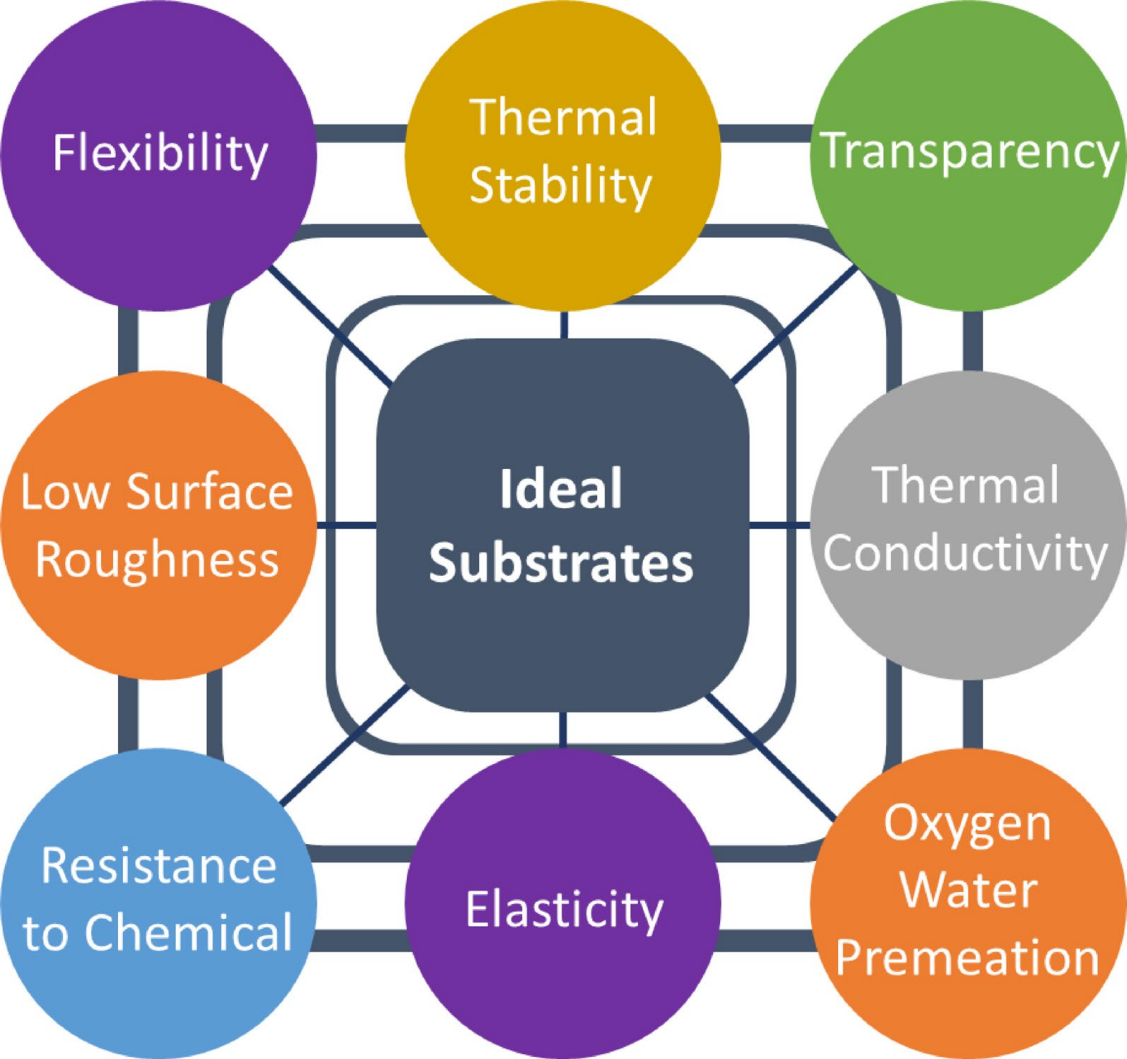
The ideal properties of substrate for flexible solar cells

The adaptability and functionality of the substrate is the foundation of any flexible electronic device. The physical and chemical properties of a substrate, especially the mechanical properties and chemical durability, significantly influence the interaction of the loaded layers with the substrate and how they connect and perform. The functionality and reliable performance of FSC systems is a direct correlation to whether the substrate is reasonably appropriate in Fig. 17. shows the simple correlation. The PCE of FPSCs depends mainly on the substrate material, as shown by differences in Table 2. For proper operation of a flexible photovoltaic device, the substrate must satisfy several elementary requirements given below [13, 69]. 

#### High flexibility

The substrate material must possess high flexibility as its foremost requirement, i.e., its flexural rigidity is to be minimized for a specified configuration and axis of bending. Practically, for specified dimensions, the substrate needs to have a low Young’s modulus so that it can bend readily and conform to mechanical stresses. In the absence of this, the substrate material should be capable of being processed into ultrathin forms that nevertheless provide the necessary flexibility [102]. 

#### High optical transmittance

The optical properties of the substrate should be carefully evaluated during the design or construction of solar cells. Reflectivity and transmissivity are two quantitative characteristics associated with optical qualities. For flexible photovoltaic applications, an ideal substrate should exhibit either high optical transparency, particularly exceeding 90% within the visible light spectrum, or enhanced reflectivity [79]. 

#### High elasticity

To accommodate the substantial strains experienced in the upper and lower layers due to bending, it is essential for the substrate material to possess a high elastic strain threshold. As a result, the minimum achievable bending radius will be sufficient for a variety of applications. The substrate must exhibit a robust restoring force when subjected to stress, ensuring its ability to revert to its original configuration even after the stress is removed [103]. 

#### High thermal tolerance

In any flexible device, several materials are integrated into substrates. Elevated temperatures are often required for certain bonding materials. To construct high-performance devices, a substrate must exhibit excellent thermal stability [83]. 

#### Strong mechanical durability

Flexible substrates must withstand substantial mechanical stress and strain when layers are added to them. Despite this, a flexible substrate should be capable of undergoing mechanical deformation and dissipating internal tension without compromising its original functional properties. Therefore, the dimensional integrity of a substrate is crucial to maintaining the performance of solar cells [104]. 

#### Lower coefficient of thermal expansion

Two parameters, the coefficient of thermal expansion (CTE) and the thermal transition temperature, play a major role in determining the thermal properties of substrates. The substrates will either expand or contract as the temperature varies due to the considerable thermal mismatch between adjacent layers. The lattice would experience additional stress, resulting in mechanical strain and potential microcracking throughout the fabrication process. The ideal substrate should possess a low CTE, closely matched to that of other materials in the integration, to prevent stress accumulation caused by repetitive thermal cycling over the course of its lifetime [105, 106].

#### Chemical solvent resistance

Substrates are exposed to a variety of chemicals during the fabrication of solar cells, including reactive gases and solvents. To avoid the release of contaminants, the substrate must remain chemically stable and inert throughout the process [107]. 

#### Surface roughness

Due to its textured surface, the substrate couldn’t be coated with a conformal protective layer to shield the device from electrical short circuits. Surface roughness must be minimized to ensure the fabrication of a fully functional solar cell. Excessive roughness levels necessitate planarization techniques such as polishing and coating, which ultimately elevate processing costs [108].

#### Excellent water and oxygen barriers

Most electrical equipment is vulnerable to oxygen and moisture, which can severely impair performance. To ensure reliable, long-term functionality, the substrate should serve as an efficient barrier layer, preventing the penetration of oxygen and water vapor [109].

#### Higher conductivity

Conduction plays a crucial role in optimizing the performance of solar cell devices. Typically, a transparent conducting electrode (TCE) is fabricated by depositing a charge-collecting layer of transparent conducting oxides onto a substrate. The surface resistance of the TCE is directly linked to the fill factor, photocurrent density, and overall photovoltaic performance of the solar cell [110]. 

**Table 2:** Lists the many substrates frequently utilized in FPSCs

Material	Strengths	Drawbacks	Future directions	Refs
Polyimide (PI)	Withstands high thermal load (up to 350 °C), low weight (1.4 g/cm^3^)	Expensive ($50/lb); moderate moisture and oxygen permeability	Develop affordable and effective encapsulation or barrier layers	[102]
Polycarbonate (PC)	Economical ($2/lb); high flexibility (70% elongation); light (1.2 g/cm^3^)	Poor UV resistance, limited barrier against gases; large thermal expansion	Design cost-effective surface passivation/barrier strategies	[103]
Polyethylene naphthalate (PEN)	Elongation up to 60%, 200 MPa tensile strength, light (1.4 g/cm^3^)	Susceptible to UV degradation, moderate barrier performance	Introducing UV-protective and barrier coatings	[104]
Polyethylene terephthalate (PET)	Low-cost ($1/lb); 70% ductility; lightweight	High thermal expansion (~ 60 μm.m^−1^.K^−1^); UV-sensitive	Develop improved low-cost encapsulation coatings	[107]
Zirconia	Corrosion and gas barrier; stable up to 1000 °C; minimal expansion	High cost ($12/lb); low flexibility and rough surface	Engineer thinner, smoother films for enhanced mechanical compliance	[108]
Lime-soda glass	Cheap ($0.60/lb); strong thermal and chemical resistance	Brittle, rough (~ 25 μm), heavy (5.7 g/cm^3^)	Minimize thickness and roughness to improve flexibility	[109]
CP titanium (Grade 1)	Extreme heat resistance (up to 600 °C); 241 MPa strength; low expansion	Poor ductility (0.05%); low tensile strength (35 MPa); expensive	Explore polishing and doping for surface enhancement	[110].
Aluminum 1050	Affordable ($1.56/lb); 2.7 g/cm^3^ density; 40% elongation at break	Surface roughness (~ 40 nm); high thermal expansion; low processing window	Surface smoothing; lower-cost dielectric layering techniques	[111]
Stainless steel 430	Low cost ($1.02/lb); oxidation-resistant; 483 MPa strength	High roughness (~ 70 nm); heavy (7.7 g/cm^3^); moderate flexibility	Enhance polishing techniques and advanced dielectric deposition methods	[112]

## Materials for active semiconductors

Various types of active materials have been extensively investigated for their suitability in FPSCs, including hydrogenated amorphous silicon (a-Si: H), copper indium gallium selenide (CIGS), small organic molecules, polymers, and perovskites [110]. We will explore the properties, limitations, and potential applications of these materials for FPSCs in the following sections.

### Amorphous silicon hydrogenated

**Fig. 18:**
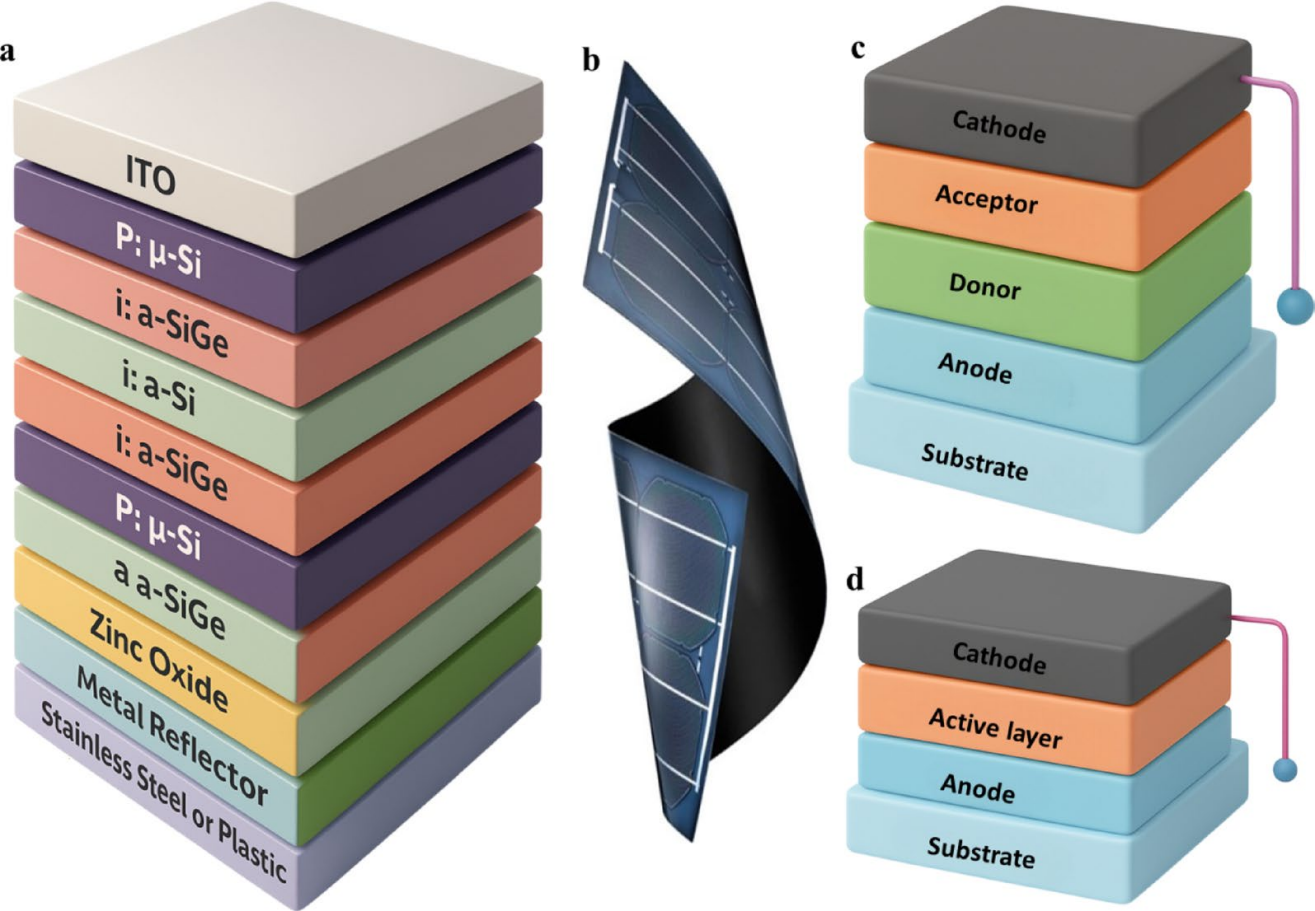
**a** Flexible silicon solar cell with triple-junction architecture utilizing µ-Si. **b** Bendable silicon heterojunction solar cell design. **c** Planar heterojunction and **d** bulk heterojunction donor-acceptor configurations, both widely applied in organic solar cells

Hydrogenated amorphous silicon (a-Si: H) is widely utilized in FPSCs due to its distinguished efficiency. The a-Si layer can be deposited using chemical vapor deposition (CVD) or plasma-enhanced chemical vapor deposition (PECVD) techniques. Despite exhibiting localized band states and elevated Urbach energies, a-Si: H can experience reduced device performance compared to crystalline silicon solar cells [113]. Incorporating hydrogen into the lattice helps mitigate deep trap states caused by dangling bonds. FPSCs with a p-i-n configuration on the a-Si: H platform has achieved power conversion efficiencies exceeding 10%. Multijunction devices employing SiGe alloys in an a-Si/a-SiGe/a-SiGe architecture have demonstrated enhanced stability and high-power conversion efficiencies [114]. This particular type of flexible Si heterojunction cell is illustrated in Fig. 18a and b.

### Copper indium gallium Selenide

Copper Indium Gallium Selenide (CIGS) materials are preferred for flexible photovoltaic applications due to their superior film quality and lower defect density compared to a-Si: H. By tuning the gallium content, CIGS can attain a bandgap exceeding 1.50 eV, typically ranging between 1.25 and 1.45 eV. Vacuum co-deposition is the most efficient method for producing high-performance FPSCs using CIGS, enabling precise control over chemical composition and thickness to engineer a graded bandgap structure. Sputtering deposition and selenization-based techniques are effective in fabricating efficient flexible CIGS solar cells. The introduction of alkali metals such as potassium for defect passivation has demonstrated promising outcomes, with potassium contributing to a record efficiency of 22.6% [109]. The active layer of FPSCs features various donor-acceptor interface structures illustrated in Fig. 18c and d. including bilayer planar heterojunction cells and bulk heterojunction cells. Bilayer cells comprise two layers of organic materials sandwiched between metallic conductors, whereas bulk heterojunction cells possess an interpenetrating network of donor and acceptor materials to maximize the interface area and facilitate efficient photocarrier generation. The phase-segregated morphology in bulk heterojunction cells aligns with the exciton diffusion length, enabling efficient exciton transport to the donor-acceptor interface [115]. 

### Semiconductors of organic materials

To enhance the efficiency of flexible organic solar cells (FOSC) on flexible substrates, optimizing the interface buffer layer and the morphological structure of the photoactive layer is paramount. The photoactive layer comprises a p-type organic semiconductor donor and an n-type organic semiconductor acceptor, and precise tuning of these components significantly boosts photovoltaic performance. The process of converting photons into electrons and holes involves four fundamental steps: photon absorption, exciton generation, exciton migration, and charge separation at the interface. Organic photovoltaic (OPV) devices are engineered with multiple layers, including a hole-transporting layer, a photoactive layer, an electron-transporting layer, and a metal cathode [110]. Traditional OPV devices employing low work function metals such as aluminum (Al) are prone to oxidation and moisture-induced degradation. Inverted OPV devices, featuring a bottom transparent electrode as the cathode and a high work function metal as the anode, provide enhanced stability in ambient conditions. Metal oxides with low work functions (e.g., ZnO or TiO_2_) are commonly utilized as the electron transport layer ETL, while metal oxides with high work functions (e.g., MoO_3_, WO_3_, or NiO_x_) function as HTL. The inverted architecture collects charges in the opposite direction to the conventional design, and the incorporation of high work function metals such as Au or Ag for the top anode ensures improved stability. Furthermore, the use of metal oxide layers facilitates compatibility with all-solution R2R processing techniques [116]. 

### Light absorption

**Fig. 19:**
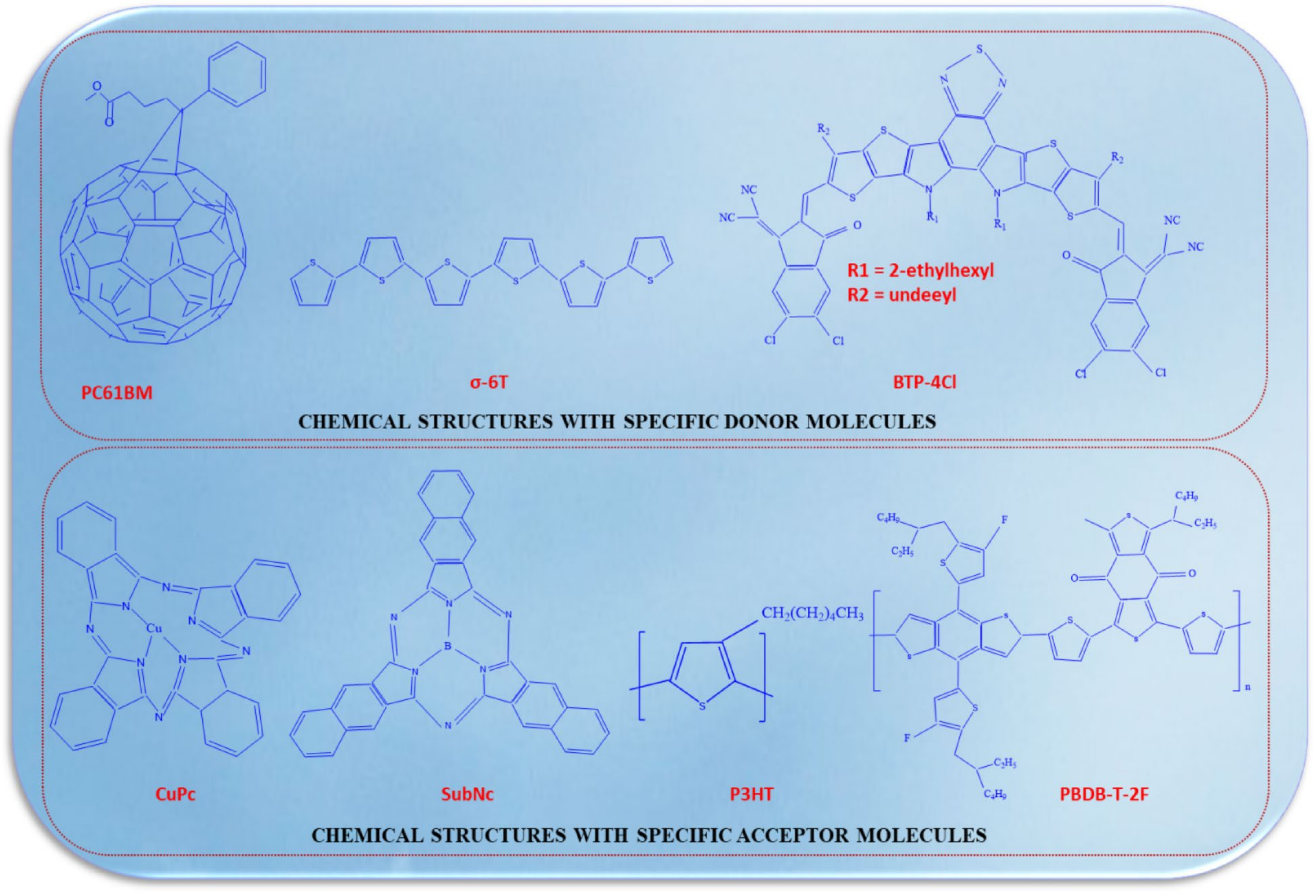
Chemical structures with specific donor and acceptor molecules

Scientists have been exploring strategies to enhance light capture in OPV systems. Poly(3-hexylthiophene-2,5-diyl) (P3HT), historically employed for light absorption, exhibits limitations due to its wide bandgap. Researchers have concentrated on developing materials with narrower bandgaps, such as fluorene copolymers, to improve PCE exhibited in Fig. 19. Narrow bandgap materials with a bandgap below 2 eV are essential for efficient photon harvesting, especially for longer-wavelength light [117]. Incorporating conjugated dyes, such as 9,10-diphenylanthracene (DPA), into the absorbers can augment light absorption and boost short-circuit current, fill factor, and PCE. Device architectural engineering, including the integration of ultrathin tin-phthalocyanine (SnPc) layers, has also demonstrated prominent potential in enhancing absorption efficiency, particularly in the near-infrared region. These innovations represent a significant milestone in the advancement of organic photovoltaics [118]. 

### Excitons diffusion

Excitons in polymeric materials typically diffuse approximately 10 nm, enabling a greater number of photoexcitons to reach the interface for free charge carrier generation. However, the active layer thickness in practical systems often exceeds 10 nm to ensure sufficient light absorption, which can result in reduced power conversion efficiencies. Rand et al. introduced sensitized phosphorescence to prolong exciton diffusion length by converting singlet excitons into triplets [112]. By incorporating platinum octa-ethyl porphyrin into poly(phenylene vinylene) at 5%, the exciton diffusion length was significantly extended, resulting in a 40% enhancement in power output of OPV devices. Researchers are focusing on minimizing donor-acceptor distances and employing multilayer architectures to optimize exciton diffusion and maximize internal efficiencies in solar cells [119]. 

### Polymer based flexible organic photovoltaics

The active layers in FPSCs typically comprise p-type organic semiconductor donors and n-type organic semiconductor acceptors exhibited in Fig. 19. These materials, including small molecules, conjugated polymers, fullerene derivatives, and non-fullerene compounds, are meticulously selected to align with the solar spectrum for efficient photon capture. The energy level difference between the donor’s HOMO and the acceptor’s LUMO significantly dictates the Voc in organic solar cells [120]. An optimal morphology, encompassing bandgap, energy levels, charge separation, and carrier mobility, is paramount for high-performance OPVs. Over time, a diverse array of donor and acceptor structures has been developed and synthesized for application in FPSCs [69]. 

## Fabrication of thin films on flexible substrates

Flexible substrates are becoming more popular by virtue of their advantages in thin-film fabrication that involve low cost, light weight, mechanical strength, and thermal stability. The substrates are well matched for uses in a broad range that includes photovoltaics, sensors, optical circuits, microelectronics, and aerospace systems. The ability of scientists to deposit cadmium sulfide (CdS) thin films on flexible substrates, including cloth, plastic foils, and even cloth-covered threads, by chemical bath deposition to form nanocrystalline structures of significant crystallinity has been demonstrated [121]. Radio frequency magnetron sputtering has been successfully used to deposit CdS onto flexible polymer substrates; it offers high deposition rates, high material purity, and material uniformity. CdTe films on polyimide substrates have shown superior performance and therefore are viable for use in space technology. Methods of vacuum thermal evaporation have also enabled CdS and ZnS films to be deposited on flexible substrates, where CdS is found to possess a greater glass transition temperature. Of specific concern are ZnS films deposited on flexible substrates, which have a narrower bandgap than films deposited on rigid analogues, upholding the promise of flexible materials for expediting solar power and optoelectronic devices [122]. 

### Flexible emerging solar cells base on plastic substrate

Flexible polymers substrates, which have become an area of great interest in photovoltaics, provide an exciting platform for maximal solar energy harvesting. They are applicable to many material systems, are consistent with R2R fabrication processes, and can be utilized on curved or irregular surfaces making them an effective delivery method for solar cells. Flexible polymer substrates also have other benefits such as resistance to mechanical stress, low weight, and ease of handling and transport [123]. While some progress is made with silicon based flexible photovoltaics and other thin-film technologies, these alternatives are prone to being limited by high production cost and low flexibility with low bending tolerance. New solar technologies such as dye-sensitized and FPSCs are set apart by their strong color tunability, ultra-thin thickness, and production processes requiring temperatures below 150 °C. These low-temperature processing conditions improve the compatibility of various polymer substrates with next-generation PV devices. This section addresses the benefits and drawbacks of these next-generation solar cells and provides a comprehensive review of the literature on FPSCs based on polymer substrates [124]. 

### Flexible dye synthesis solar cell

**Fig. 20:**
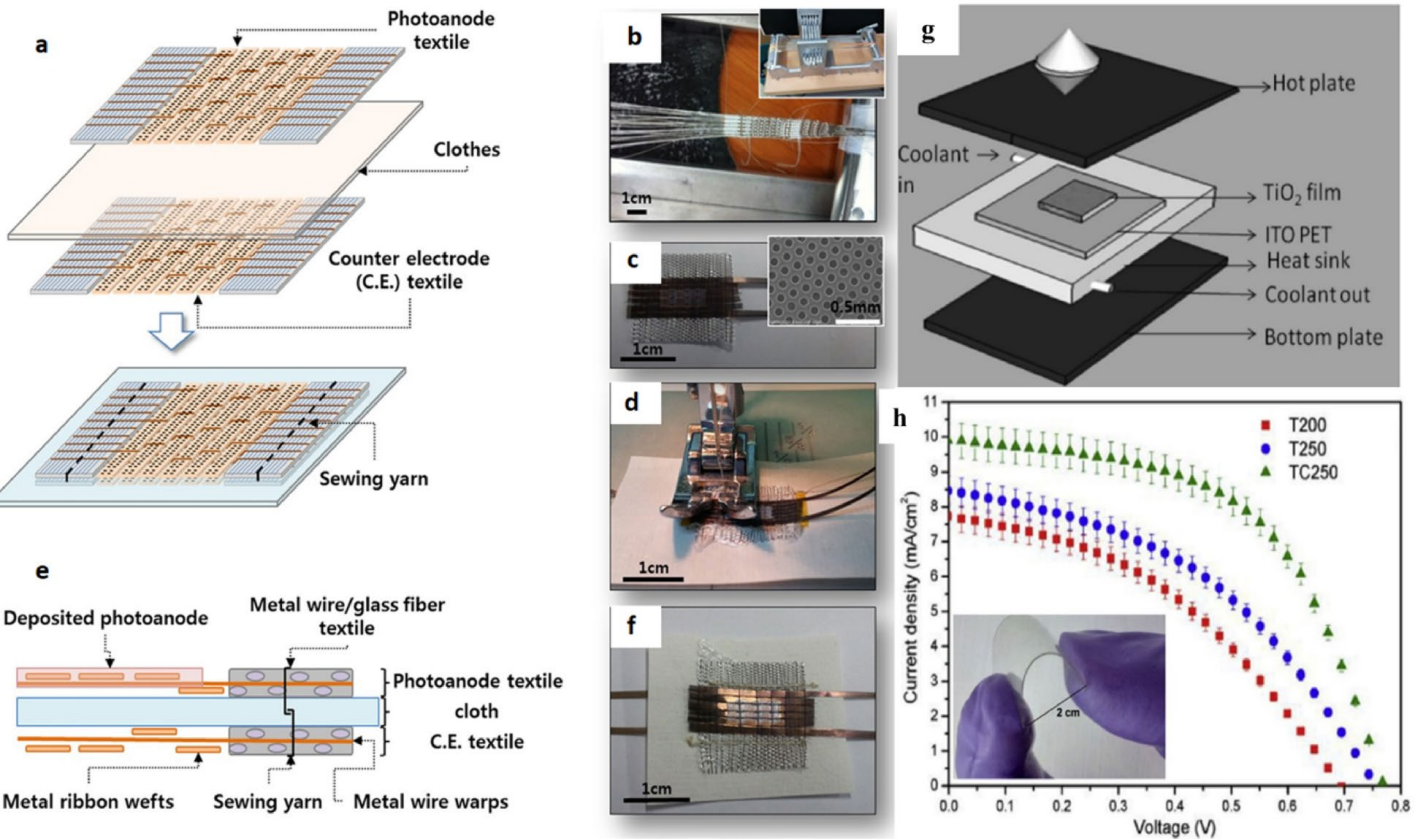
**a** The production of textile DSSCs involves stitching electrodes onto fabric or paper. **b** Electrodes are woven into textiles using a loom. **c** Electrodes are crafted from stainless-steel ribbons featuring periodic perforations. **d** Hanji paper is used as a substrate for textile DSSCs. **e** A textile-based DSSC created through sewing electrodes. **f** A core-integrated textile DSSC with electrodes sewn onto Hanji material. Reproduced with permission from reference [127].Copyright 2014 Nature Portfolio. **g** Deposition of TiO_2_ onto ITO PET polymer substrates occurs at elevated temperatures. **h** Photovoltaic current-voltage responses of flexible DSSCs using TiO_2_ and composite materials are shown for various temperature conditions. Reproduced with permission from reference [13]. Copyright 2020 John Wiley & Sons

Researchers have been striving to develop efficient and cost-effective solar systems to address global energy challenges. The concept of the dye sensitized solar cell, launched by O’Regan and Gratzel in 1991, introduced a thin-film approach to solar energy conversion, using a photo-electrochemical process to turn visible light into usable electricity. Substantial research has been conducted on flexible DSSCs employing polymer substrates, as illustrated in Table 3. DSSCs are environmentally benign, facile to manufacture, and suitable for both indoor and outdoor applications. While they surpass thin-film solar cells in performance, they remain less efficient than crystalline solar cells [125]. Conductive substrates, typically composed of plastics or polymers such as ITO-PET or ITO/PEN as exhibited in Fig. 20a–f are commonly employed to fabricate flexible DSSCs, facilitating portability and customization. Electrophoretic/electrochemical deposition (EPD) is a key component of chemical fabrication methods. Among the standard manufacturing approaches are doctor blading, screen-printing, laser pulse deposition, electrospray application, and electrophoretic techniques. Figure 20g and h offers a preliminary depiction of the cost-efficient, versatile, and straight forward deposition methods. Sintering approaches such as laser based and heat sink supported high temperature processes are compatible with plastic substrates and do not result in damage [126]. 

**Table 3:** Extensive research on flexible DSSCs utilizing polymer substrates

Photoanode materials	Conductive substrate	Dye; electrolyte	Counter electrode material	Highlights	Efficiency (%)	Refs
TiO_2_	ITO-PET	N_3_ dye	CuInS_2_/PEDOT: PSS/ITO-PET	–	3.99	[128]
TiO_2_	ITO-PET	N_3_ dye	PEDOT: PSS/ITO-PET	–	1.56	[110]
TiO_2_	ITO-PET	N_3_ dye	CuInS_2_/PEDOT: PSS/cellulose paper	–	1.06	[129]
Bilayer TiO_2_	ITO-PET	N_719_ dye/Scattering layer; liquid	Pt	–	6.33	[130]
TiO_2_-P_25_	ITO-PET	N_719_; liquid	Pt/ITO-PET	By employing UV-O_3_ treatment, the presence of organic contamination on the surface of TiO_2_ was effectively reduced.	2.53	[131]
NiONP/Cdot	ITO-PET	N_719_ dye; electrolyte	Pt-ITO/PET	The substrate used in this process was a biodegradable cellulose nanofiber (CNF) sheet that underwent TEMPO oxidation.	4.43	[132]
NiONP/Cdot	ITO/ODA-TOCNF		PPy/TOCNF		1.45	
NiONP/Cdot	NiO NW/TOCNF		Pt-TOCN		1.30	
ZnONP/Cdot	ZnO NW/Cdot/TOCNF		Pt-PPy/TOCNF		1.34	
TiO_2_-P_25_	ITO-PEI	N_3_; gel	Pt/ITO-PEI	The surface of the PEI (flexible substrate) was treated using plasma without chemical sintering.	2.25	[131]
TiO_2_	Ti foil	N_719_; gel	PEDOT: PSS/ITO-PET	Spin coating	1.7	[127]
				Screen printing	1.33	
TiO_2_	ITO-PET	N_719_ dye	Pt/ITO-PET	–	4.11	[120]
TiO_2_-P_25_	ITO-PEI	N_3_; iodide gel	Pt/ITO-PEI	–	2.8	[125]
TiO_2_	FTO-Glass	N_3_-N_719_; liquid	Nanodiamonds/Zn/ITO-PET	–	6.23	[126]
TiO_2_-P_25_	FTO-PET	N_719_-BVImI; liquid	MoS_2_/FTO-PET	–	4.84	[13]
TiO_2_-TG LSL	ITO-PEN	N_719_; liquid	Pt/ITO-PEN	–	5.18	[122]
TiO_2_	ITO-PET	N_3_; phthaloyl chitosan gel	CoS_2_/Ag-PET	–	7.09	[123]
			Pt/ITO-PET	–	7.04	
TiO_2_	ITO-PET	N_719_ dye	C/Graphite/C-black/graphene/ITO-PET	–	4.32	[124]
TiO_2_ nanotube arrays	Cu-Ti foil	N_719_; electrolyte	CuS/PET	CuS film act as conducting and catalysts for flexible DSSCs	4.52	[133]
			Pt/PET		3.24	
TiO_2_	Ag-Kapton	N_719_; liquid (Iodide)	Pt/FTO glass	The Kapton base devices have high temperature and chemical resistance, uniformity, and printed layer quality.	7.03	[134]
TiO_2_	PET/PA/PEDOT: PSS	N_719_; quasi-solid electrolyte (PAN)	RGO/PEDOT: PSS-PET	Excellent flexibility	2.59	[135]

### Flexible solar cell

**Fig. 21:**
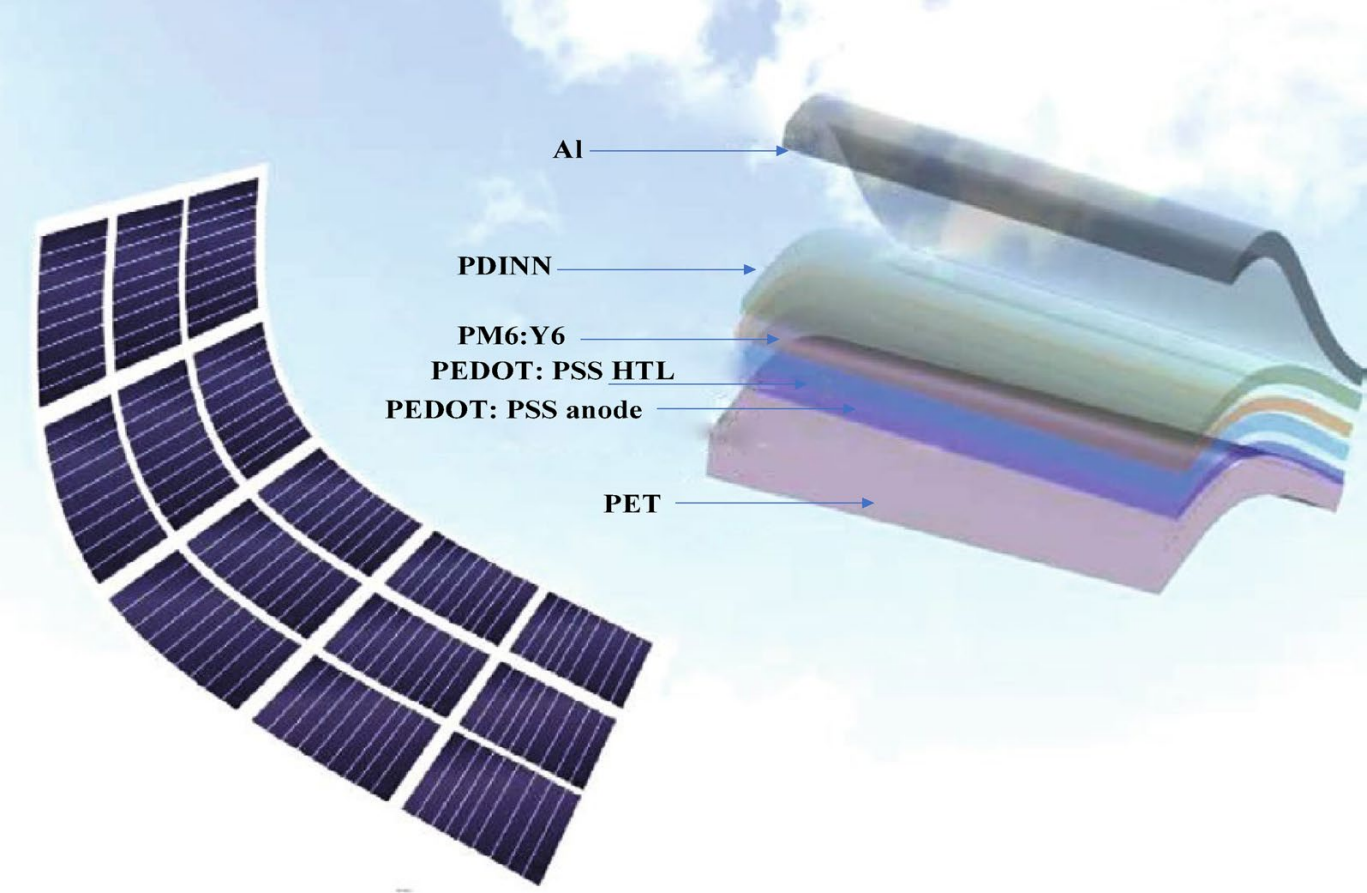
Polymer supported flexible perovskite photovoltaic devices. Reproduced with permission from ref [138]. Copyright 2021 Springer

FPSCs have emerged as extremely promising solar photovoltaic devices with record-breaking efficiency and cost-effective scalability for mass production in large areas. The highly abundant precursor material availability makes FPSCs hugely valuable for mass production compared to other thin-film solar technologies. The cells impart outstanding advantages such as superior mechanical flexibility, ultra-lightweight design, and visibility through partial transparency, making them strong contenders for solar solutions in the future. The addition of perovskite solid-state absorber layers substantially increased solar cell efficiencies and rendered FPSCs a high-tech evolution of solid-state DSSCs [136]. Typically, FPSCs formed on polymer substrates provide an ideal platform for the fabrication of flexible electronics, as demonstrated in Fig. 21. due to their solid-state nature and compatibility with low-temperature processing. FPSCs are made up of a multilayer sandwich structure where the perovskite absorber is sandwiched between electrodes and ultrathin charge transport layers. The perovskite material has a similar crystalline framework to that of calcium titanate, which renders it rich in optical properties and unparalleled electrical conductivity. HTL and ETL, with varying electron affinities and ionization energies, contribute towards charge recombination minimization by selective blocking of holes or electrons. Some common materials used in these layers are spiro-OMeTAD, P3HT, ZnO, and TiO_2_ [137]. Solution deposition techniques such as spin coating and inkjet printing enable the deposition of FPSCs on flexible polymeric substrates, making them the ideal candidate for wearable and portable applications. Recent research includes the engineering of FPSCs being flexible through the utilization of newly synthesized polymers, as listed in Table 4. The growth of polymer FPSCs is still a vigorously researched area due to their acceptable mechanical flexibility, low weight, and ease of production onto varied surfaces [138]. 

**Table 4:** Flexible perovskite solar cells are based on recently developed polymers

Substrate	PCE (%)	Active area (cm^2^)	Device architecture	Perovskite composition	Deposition technique	Operational stability (under MPP/heating/cyclic stress)	Refs
PEN	17.31	0.10	ITO/PEDOT: PSS: Zn(NTf_2_)_2_/MAPbI_3_:PVA/PCBM/Ag	MAPbI_3_:PVA	Spin coating	85% Retention after 500 h under MPP at RT	[139]
PET	18.66	0.09	ITO/PTAA/2D BOC Spacer(FPEAI)/CsFAPbBr_3_/C_60_/BCP/Ag	CsFAPbBr_3_	Spin coating	Maintained 90% PCE after 200 bending cycles (*r* = 5 mm)	[140]
PET	14.66	0.08	ITO/SnO_x_/PNPs:@NiO_x_@PAM/Spiro-OMeTAD/Au	FAPbI_3_	Solution processed	80% Retention after 400 h at 85 °C	[13]
PI	19.20	0.16	GR/PEDOT: PSS/MAPbI_3_/FAPbI_3_/CSC/BCP/PI/GR	MAPbI_3-_FAPbI_3_	Hot-casting	Stable after 1000 bending cycles (*r* = 5 mm)	[141]
PET	25.00	0.09	MB-NO_x_/C_60_/ALD-SnO_2_/Au/PEDOT: PSS/NBG perovskite/C_60_/BCP/Cu	NBG triple-cation	Sequential vacuum + spin coating	Retained 95% after 1000 h under MPP	[33]
PET	11.96	0.10	PTAA/BAFACS/PCBM/BCP/Ag	(BA, FA, CS)PbI_3_	Spin coating	75% After 200 h at RT	[142]
PET	12.43	0.10	PTAA/FAFACS/PCBM/BCP/Ag	(FA, FA, CS)PbI_3_	Spin coating	80% Retained after 250 bending cycles	[143]
PET	14.57	0.12	IZO/PTAA/BAFACS/PCBM/BCP/Ag	(BA, FA, CS)PbI_3_	Spin coating	85% Retention after 500 h MPP tracking	[144]
PET	11.40	0.09	SnO_2_/perovskite/Spiro-OMeTAD/Ag	MAPbI_3_	Spin coating	80% After 100 bending cycles	[145]
PET	9.96	0.08	ZnO/SnO_2_/perovskite/Spiro-OMeTAD/Au	MAPbI_3_	Low-temp spin coating	70% After 100 h at 85 °C	[146]
PET	9.49	0.10	PH_1000_/A_l103_/MAPbI_3_/PCBM/Ag	MAPbI_3_	Spin coating	Retained 88% after 300 h heating	[147]
PET	11.20	0.09	ITO/MAPbI_3_/PCBM/Ag	MAPbI_3_	One-step spin	75% After 100 h under MPP	[148]
PEN	19.90	0.16	bil-SnO_2_/mp-TiO_2_/perovskite/Spiro-OMeTAD/Ag	MAPbI_3_	Spin coating	92% Retention after 800 h MPP	[149]
PET	14.90	0.10	SnO_2_/MAPbI_2_/Spiro-OMeTAD/Au	MAPbI_2_	Spin coating	Stable after 200 bending cycles	[150]
PET	12.00	0.09	SnO_2_/Perovskite/Spiro-OMeTAD/Au	MAPbI_3_	Spin coating	Retained 80% after 500 h heating	[136]
PET	10.26	0.08	PEDOT: GOMAPbI_2_/PC61BM/Ag	MAPbI_2_	Spin coating	75% After 150 h heating	[100]
PET	17.50	0.09	c-TiO_2_/mp-TiO_2_/FAPbI_2_/Spiro/Ag	FAPbI_2_	Solution process	Stable after 300 bending cycles	[151]
PET	13.33	0.08	nano-TiO_2_/MAPbI_2_/Spiro/Au	MAPbI_2_	Spin coating	80% After 100 h heating	[152]
PET	12.07	0.09	PTAA/PVK/C_60_/SnO_x_/ITO/Ag/ITO	FAPbI_3_	Sequential spin	85% After 500 h at RT	[153]
PI	19.46	0.16	Cu grid graphene/PEDOT: PSS/perovskite/PC61BM/ZnO/Ag	FAPbI_3_	Spin coating	Stable after 1500 bending cycles	[154]
PET	16.08	0.09	SnO_2_/perovskite/Spiro-OMeTAD/Ag	MAPbI_3_	Spin coating	90% After 1000 h MPP	[155]
PET	15.71	0.10	IZO/PTAA/perovskite: PCBM: PMMA/Cr/Carbon	Mixed-cation	Spin coating	85% After 1000 h (RT)	[154]
PEN	15.36	0.09	ITO/Au-TiO_2_/TiO_2_/FAPbI_3_/Spiro/Ag	FAPbI_3_	Spin coating	Stable under 85 °C heating	[137]
PET	16.10	0.10	R-Ru/Lt-TiO_2_/FAMAPb(IBr)_3_/Spiro/Ag	FAMAPb(IBr) _3_	Spin coating	90% After 300 h MPP	[156]
PET	17.29	0.09	ITO/NiO_x_/perovskite: PC_61_BM/BCP/Ag	MAPbI_3_	Solution process	Retained 92% after 1000 h (RT)	[157]
PEN	17.29	0.10	PEALD TiO_x_/RIE-treated mp-TiO_x_/perovskite/Spiro/Au	MAPbI_3_	ALD + spin coating	88% After 800 h (MPP)	[158]
PET	17.90	0.10	HC.dhd-PH1000/Cu: NiO_x_/MAPbI_3_/PCBM/BCP/Cu	MAPbI_3_	Slot-die coating	92% After 1000 bending cycles	[159]
PET	20.40	0.16	modified SnO_2_/perovskite/Spiro-OMeTAD/Ag	Triple-cation	Blade coating	90% After 1000 h MPP	[160]
PET	17.00	0.09	TiOx/PCBM/Gual-treated perovskite/Spiro/MeOTAD/Au	FAMAPbI_3_	Vacuum + spin	87% After 500 bending cycles	[161]
PET	14.98	0.08	C_60_/TiOx/FAMAPb(IBr)_3_/Spiro/Au	FAMAPb(IBr)_3_	Spin coating	85% After 500 h MPP	[162]
PET	18.53	0.10	SnO_x_/KCl/MAPbI_3_/Spiro-OMeTAD/Ag	MAPbI_3_	Spin coating	88% After 1000 h heating	[163]
PEN	17.10	0.10	ITO/FAMAPb(IBr_3_)/PTAA/Au	FAMAPb(IBr)_3_	Spin coating	90% After 500 h MPP	[138].
PET	17.50	0.09	SnS_2_/MAPbI_3_/Spiro/Au	MAPbI_3_	Spin coating	80% After 300 h heating	[164]
PES	14.80	0.09	ITO/SnO_2_/FAPbI_3_/Spiro/Au	FAPbI_3_	Spin coating	Stable after 200 bending cycles	[145]
PES	18.90	0.10	SnO_2_/ZnO/AZO/PES	FAPbI_3_	Sputtering + spin coating	95% After 1000 h (60 °C)	[165]
PET	15.76	0.09	ED-TiO_2_/BK-TiO_2_/MAPbI_3_/Spiro/Au	MAPbI_3_	Spin coating	90% Retention after 500 h heating	[166]
PEN	16.80	0.09	Graphene/MoOx/PEDOT: PSS/MAPbI_3_/C_60_/BCP/LiF/Al	MAPbI_3_	Sequential spin	Retained 85% after 1000 h MPP	[167]

## Flexible perovskite solar cell fabrication techniques

Figutre 22 shows various fabrication techniques for flexible solar cells, divided into large-scale and small-scale methods. Large-scale methods include blade coating, spray coating, slot die coating, inkjet printing, and R2R processing, which are critical to produce high-throughput and low-cost devices. Small-scale techniques, including spin coating and thermal evaporation, are typically for laboratory-scale fabrication and control of material deposition [168]. These methods are important to be utilized in optimizing flexible solar cell performance as well as the scalability mentioned in detailed given below.

**Fig. 22:**
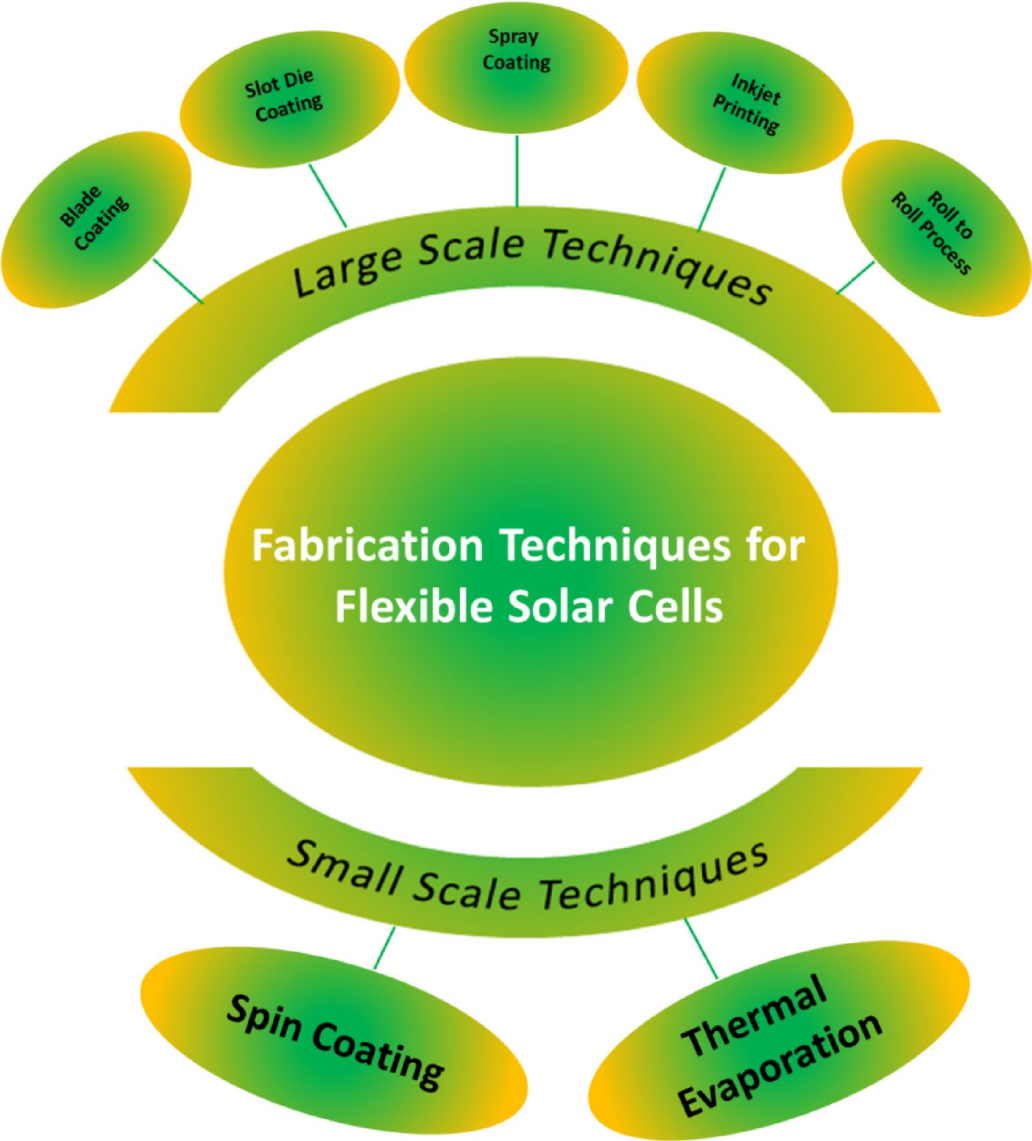
Flexible solar cell fabrication techniques

### Laboratory scale coating techniques

Lab-scale fabrication processes, as shown in the figure, include processes like spin coating and thermal evaporation. Spin coating is typically used for deposition of thin uniform films with precise thickness control on the active layers of flexible solar cells. Thermal evaporation is a vacuum process offering high-precision metal or semiconductor material deposition. These low-volume processes find the best application in research and development where high-quality films and very closely controlled deposition are required to maximize device performance before scaling up into production [32] (Table 5).

**Table 5:** Flexible solar cell fabrication techniques

Method	Application insight	Merits	Limitations	Refs
Spin-coating	Preferred in academic and lab-scale settings for thin-film deposition due to its ability to generate a uniform coating over a small area.	Economical process Simple operation and fast drying Suitable for flat substrates Wide thickness controllability	Not scalable for industrial production Inconsistent film thickness Inefficient on non-flat surfaces Material loss (~ 90%)	[167]
Thermal evaporation	Used for precise, high-purity thin-film fabrication by sublimating a single source material in vacuum conditions.	Minimal thermal stress on substrates High deposition rate High material purity Directional film growth control	Poor step coverage Hard to manage film stoichiometry Temperature control of substrate can be difficult	[169]

#### Spin coting and thermal evaporation

**Fig. 23:**
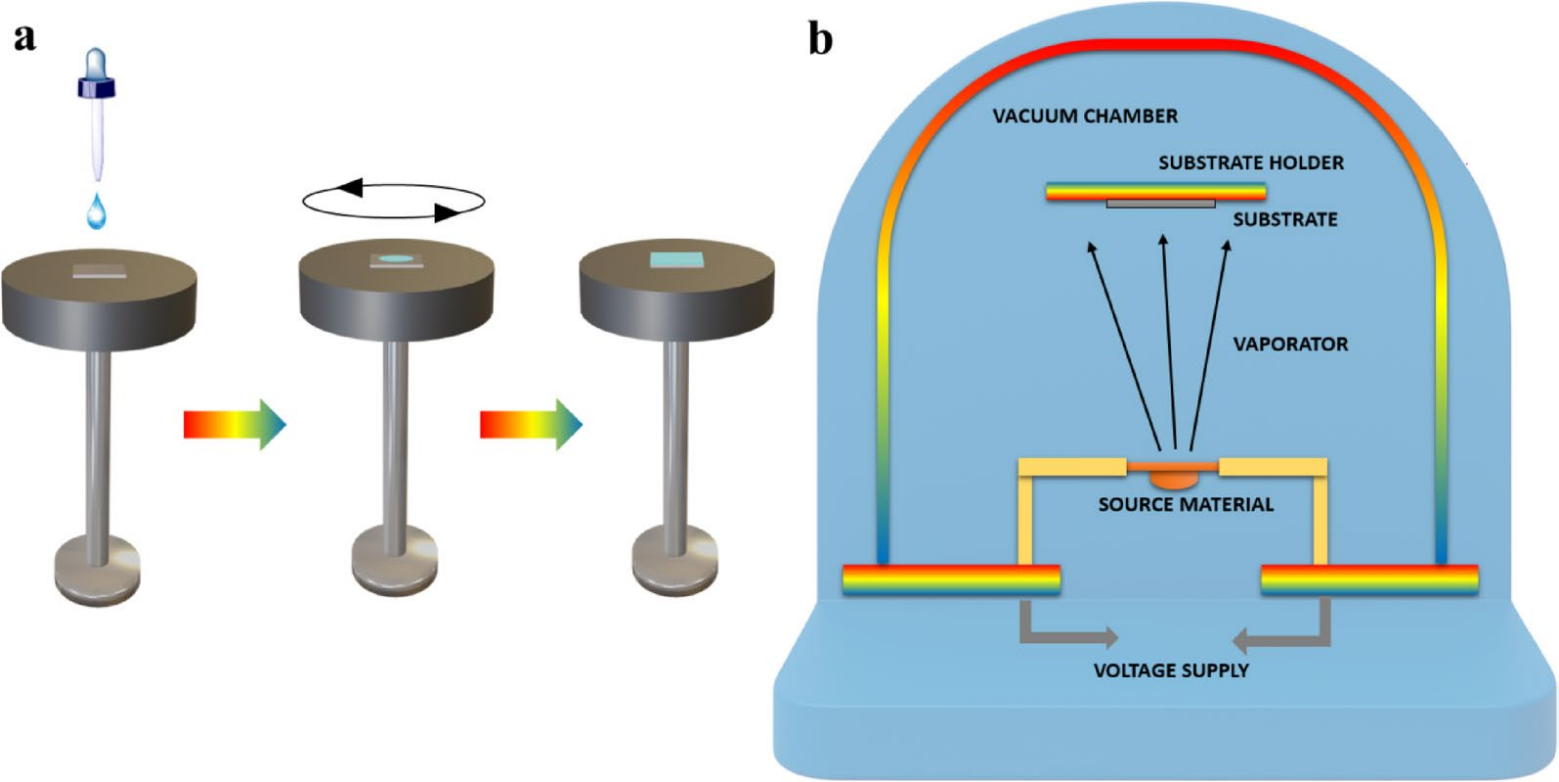
**a** Spin coating works on principle of centrifugal force. **b** Spray coating working on principle of thermal vaporization

Spin-coating is a widely adopted technique for fabricating uniform, defect-free perovskite thin films, offering precise modulation of film thickness, as illustrated in Fig. 23a. Post deposition thermal treatments are imperative to facilitate perovskite crystal formation, with specific temperature requirements dictated by the perovskite composition. Low-temperature annealing at approximately 100 °C is adequate for prevalent formulations such as Cs_0.05_(MA_0.17_FA_0.83_)_0.95_Pb(I_x_Br_1−x_)_3_. Although the spin-coating method has set benchmark efficiencies for FPSCs, scalability issues persist due to the necessity for meticulous control during the deposition process [170]. The two-step spin-coating approach has demonstrated superior film morphology and enhanced interface regulation compared to the one-step process; however, incomplete PbI_2_ conversion remains a constraint to its broad industrial adoption. Despite these drawbacks, spin-coating continues to be a critical laboratory-scale strategy for generating high-efficiency FPSCs [171]. Figure 23b illustrates that thermal evaporation (spray coating) constitutes an alternative route for producing smooth and homogeneous perovskite layers by vaporizing precursor materials in a vacuum chamber. This can be accomplished through either single-source or dual-source evaporation, each with distinct advantages and operational challenges. While dual-source evaporation enables the co-evaporation of both organic and inorganic precursors, it is currently economically unfeasible for large-scale commercialization [172]. 

### Industrial scale coating techniques

Various deposition techniques such as blade coating, slot-die coating, spray coating, inkjet printing, and R2R processes have been thoroughly examined for FSC materials, as outlined in Table 6. Collectively, these methods exhibit distinctive advantages that significantly contribute to the fabrication of high-efficiency FPSCs by enabling precise film formation, scalability, and compatibility with flexible substrates [173]. 

**Table 6:** Commercial scale coating techniques for FPSCs

Fabrication	Key points	Advantages	Disadvantages	Refs
Blade coating	This technique works effectively for creating thicker layers even though it cannot produce uniformity at the nanoscale. Its scalability, ease of use, and adaptability make it the ideal method for industrial manufacturing	Quick and effective Less waste of materials It is suitable for scalable production The fabrication area has a less significant impact on uniformity A variety of variables, including the substrate temperature, blade speed, viscosity of solutions, etc., can be tuned to create the necessary thin films	Reproducibility is generally poor Cannot create extremely thin films Process-related contamination is simple	[174]
Slot-die coating	When the manufacturing process is developed sufficiently, slot-die coating is a strong option for industrial production	A lot of uniformity Less waste of materials Fast coat application It is suitable for scalable production A wide range of thick adjustments are possible	Expensive initial setup Due to the system’s complexity, there are more sources of problems, making diagnosis challenging The equipment frequently requires a sizable supporting infrastructure Due to the complexity of the procedure, extensive training is required	[175]
Spray coating	Large-scale polymer material depositions are ideally suited to this production technique	Less waste of materials High speed of fabrication The capacity to print patterns It is suitable for scalable production There is less damage to the underlying layers	Leveling of ink on surfaces Relatively high film roughness Narrowing the processing window	[176]
Inkjet printing	This method is expected to be utilized for mass production. Inkjet printing can be used to assemble a single perovskite solar cell once carbonaceous materials have taken the place of metal electrodes	Cost-efficient Speedy coat application The substrate sustains less damage Coating substrates using pre-designed patterns Adaptable to manufacturing that is scaleable	Variable roughness The inks must be strick Regular maintenance is necessary The uniformity of films is relatively low	[177]
R2R process	Cost-effective and scalable manufacturing technology permits efficient production of solar cells on flexible substrates, although with process control and material compatibility problems	Cost-effective Provides speedier processing It makes it possible for massive production Precision, thickness, and layer uniformity Versatile with a variety of substances and materials	Variation in output quality Complexity of equipment Increased defect rates Limited material compatibility Limited thickness contro	[178]

#### Blade coating

**Fig. 24:**
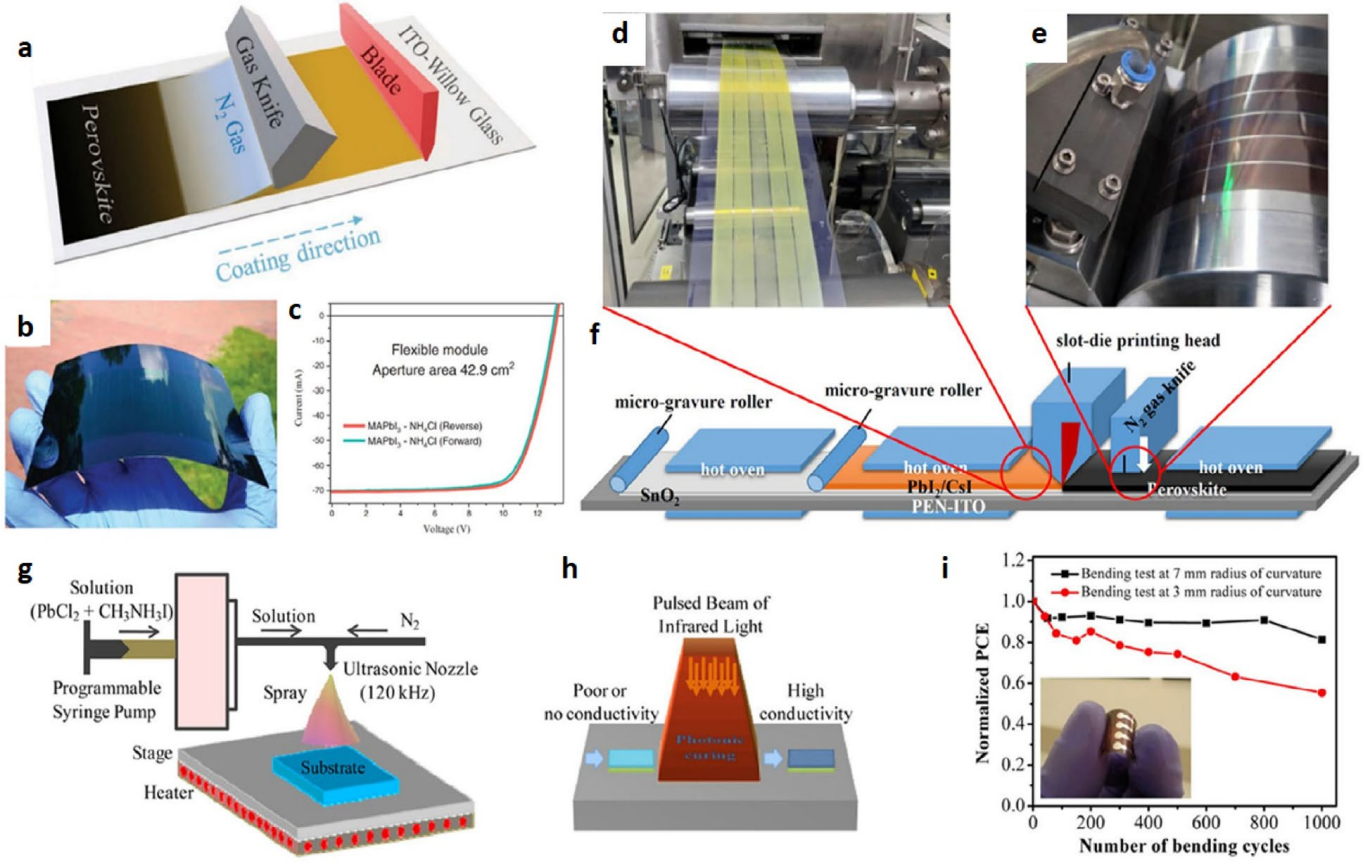
**a** Blade coating method on ITO-Willow Glass using a N_2_ gas knife. **b** A picture of an adaptable perovskite module. **c** The flexible perovskite modules J–V curve. Reproduced with permission from reference [182]. Copyright 2020 John Wiley & Sons. **d** Large-area PbI_2_/CsI films were formed using a R2R micro gravure printing technique, at thermal treatment. **e** N_2_ gas knife was employed during slot-die coating to apply triple-cation halide salts onto preformed PbI_2_/CsI layers. **f** R2R printed FPSCs with a triple-cation combination. Reproduced with permission from reference [183]. Copyright 2020 John Wiley & Sons. **g** The procedure of ultrasonic spray coating. **h** Optical curing improved the performance and dependability of the device. **i** Normalized PCE of flexible devices after bending tests performed at curvature radii of 7 mm and 3 mm. Reproduced with permission from reference [32]. Copyright 2023 John Wiley & Sons

To achieve greater precision, Dai et al. applied blade coating for FPSC module fabrication on willow-glass, as demonstrated in Fig. 24a–c. In their investigation, the integration of an N_2_ gas knife during the crystallization of wet films at ambient temperature significantly accelerated the formation of FPSC modules [179]. However, prudence is required, as the use of an air-knife may induce the formation of a capping layer, which can inadvertently result in pinhole defects within the perovskite film. Additives such as NH_4_Cl are currently under investigation to enhance film uniformity and mitigate trap-state density. Recent breakthroughs in bar-coating techniques for FPSCs have demonstrated noteworthy efficiencies reaching up to 21.76%, underscoring their viability for scalable fabrication. Furthermore, non-vacuum deposition strategies, including D-bar coating for electrode formation, have facilitated the development of FPSCs with power conversion efficiencies as high as 16.7%, rivaling those equipped with thermally evaporated gold electrodes [180]. Meniscus-assisted deposition relies on forming a concave meniscus between substrate and blade to boost the nucleation and crystallization rate of perovskite layers. Meticulous optimization of parameters such as temperature, ambient environment, and solution chemistry is critical to promote robust grain growth and achieve high-quality film formation [192]. Preheating the substrate can induce rapid supersaturation and accelerate solvent volatilization, thereby enhancing film uniformity and consistency. Meniscus-guided coating is employed in FPSC modules and has demonstrated impressive PCE, that was 15.17% over a 31.2 cm^2^ active area. Blade coating, recognized for its versatility, is compatible with a variety of substrates and supports continuous, scalable fabrication of perovskite modules. However, mitigating pinhole defects remains pivotal for further refinement and optimization of this technique [181]. 

#### Slot-die coating

The slot-die coating technique passes solution through a fine slit onto the substrate, enhancing deposition control and reducing precursor loss. The resulting film thickness is governed by several parameters, including substrate translation speed, solution concentration, and flow rate, each exhibiting inverse or direct correlations depending on process conditions [184]. Perovskite precursors readily volatilize and reach a state of supersaturation, significantly influencing film homogeneity and overall quality. Slot-die coating research has predominantly targeted rigid substrates, demonstrating success through both single-step and sequential deposition techniques on glass. Bu et al. attained remarkable PCEs of 20.42% and 19.54% by employing slot-die coating for formamidinium (FA)-based devices across varying surface dimensions [185]. Galagan et al. implemented a single-step slot-die coating process to deposit the ETL and perovskite layers, attaining a 13.5% PCE in their FPSCs. To mitigate the issue of solvent toxicity, a low-toxicity solvent blend comprising DMSO and 2-butoxyethanol was employed as an alternative to DMF. For morphological control, two-step deposition strategies were implemented in a R2R configuration under ambient conditions, as illustrated in Fig. 24d and e. In this approach, the organic salt was subsequently coated via slot-die following the PbI_2_ layer, facilitating the in-situ formation of the perovskite layer. The resulting device attained a productivity yield of 11.0%. Beyond perovskites, comprehensive investigations are still required to optimize slot-die coating parameters for the remaining functional layers [186]. 

#### Inkjet and spray printing

Printing is a technique widely employed in industry and is particularly suitable for large-scale surface coverage. Print-coating functions on a principle analogous to that of a conventional office printer. In this approach, the nozzle deposits droplets of ink directly onto the substrate. Inkjet printing achieves precise droplet sizing by varying the pressure in a microfluidic chamber, whereas spray coating uses a dedicated atomizer to produce a mist of very small droplets. Figure 24g presents a schematic representation of the printing methodologies. The droplets coalesce to form a uniform wet film during the deposition stage. Therefore, the trajectory of droplet flight must be meticulously controlled to ensure a homogeneous precursor layer. A solidified thin film is then established as the solvent gradually evaporates [187]. 

Wilk et al. developed a green-solvent-compatible inkjet printing precursor. This approach provides a scalable fabrication strategy suitable for industrial deployment using a non-toxic, environmentally benign solvent system. FPSCs printed via inkjet on PET substrates achieved a of 11.4% across a 1 cm^2^ active area. Spray printing, in contrast, is particularly compelling due to its cost-efficiency, high-throughput capability, and accelerated production rates, making it more advantageous than inkjet printing for mass manufacturing. A variety of atomization mechanisms including high-velocity gas flow, ultrasonic excitation, or electrostatic repulsion can be employed to achieve fine droplet formation in spray printing. Figure 24h and i illustrates a schematic depiction of the spray coating setup, highlighting both the optical attributes and the resulting device performance metrics in terms of PCE. Das et al. have introduced a FPSC utilizing an ultrasonic spray deposition technique, demonstrating an impressive PCE of 8.1% and exceptional mechanical resilience, retaining stability even after 1000 bending cycles. The processing window is a critical parameter for ensuring film uniformity across large-area substrates during perovskite layer deposition via spray coating. Optimization of the precursor ink with methylamine chloride led to prominent efficiencies of 18.5% in rigid devices and 16.15% in flexible counterparts [188]. 

#### Roll-to-roll process

**Fig. 25:**
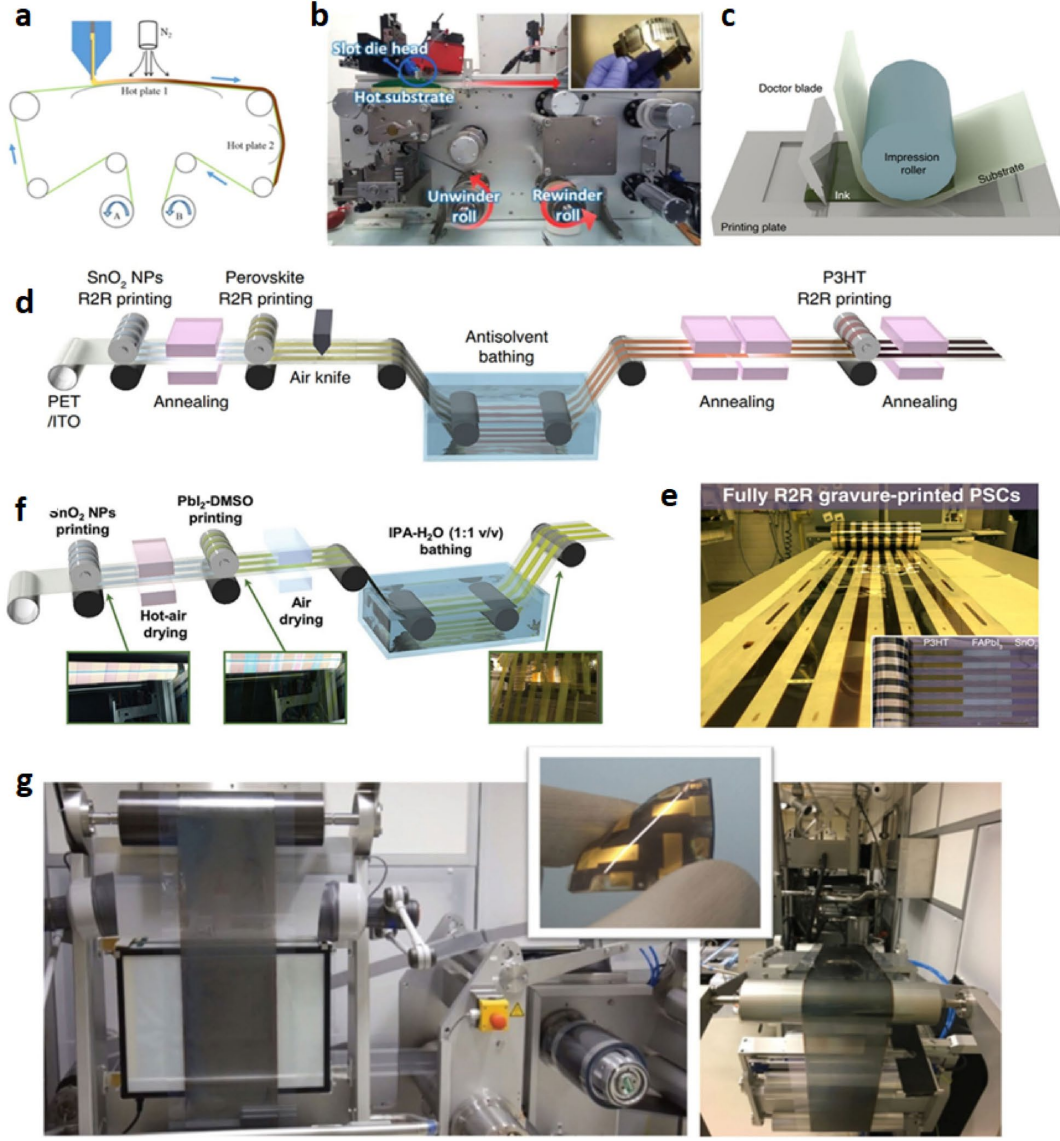
**a** Graphic depicting the sequential deposition of the perovskite layer through R2R processing. Reproduced with permission from reference [193]. Copyright 2018 Elsevier. **b** Photograph of the experimental arrangement configured for R2R fabrication. Reproduced with permission from reference [194]. Copyright 2019 John Wiley & Sons, Inc. **c** Tabletop gravure printer schematic. **d** Process flow diagram illustrating R2R production of FPSCs. **e** Photo of FPSCs after complete R2R manufacturing, with inset visuals detailing layer-by-layer separation of the device components from the roll. Reproduced with permission from reference [190]. Copyright 2020 Springer Nature. **f** Visualization of the sequential two-step R2R approach designed for fabricating FPSCs. Reproduced with permission from reference [191]. Copyright 2019 John Wiley & Sons, Inc. **g** Photographs capturing samples of fabricated flexible devices alongside the R2R coated perovskite films. Reproduced with permission from reference [192]. Copyright 2019 John Wiley & Sons

Unlike traditional techniques, R2R printing is uniquely suited for flexible substrates and has proven effective in producing a wide range of electronic devices. Figure 25a and b provides an illustration of the apparatus used in this process. By incorporating NH_4_Cl to assist in crystallization in the presence of air, an impressive PCE of 11.16% was achieved [189]. In addition to the scalable techniques mentioned earlier, another viable method for the coating process is precast gravure. When targeting a perovskite layer thickness of approximately 1 μm, the microgravure technique is preferable. Kim’s team established a R2R gravure printing process for perovskite layer deposition. In this system, an engraved cylinder delivers precursor solution to a substrate, compressed by an impression roller. Film uniformity is controlled by a blade that removes excess material. Figure 25c–e presents the resulting FPSCs fabricated entirely through this technique [190]. To support the formation of high-quality crystals in the perovskite blend (FAPbI_3_)_0.95_(MAPbBr_3_)_0.05_, a combination of tert-butyl alcohol and ethyl acetate was used as an antisolvent. FPSCs produced through this method on a pilot line attained PCE of 13.8% and displayed a well-organized surface morphology. Figure 25f depicts R2R printing, which also enabled a two-step deposition process. Micro-gravure printing was used to create the PbI_2_/CsI film on top of the SnO_2_ layer. The reaction was then finished by slot-die coating a mixed halide salt solution onto the already-prepared film [191]. 

This integrated approach enabled gravure printing to achieve a prominent PCE of 10.57%, thereby demonstrating the feasibility of combining R2R processes with rigid substrate coating techniques. There have been several integrated demonstrations in this domain to date. During R2R deposition, Kim et al. demonstrated a hot slot-die coating process enhanced by polyethyleneimine ethoxylate to improve ambient humidity tolerance in perovskite films, resulting in an 11.7% PCE. Likewise, Galagan et al. achieved 13.5% efficiency via a R2R slot-die approach, as illustrated in Fig. 25g. The refinement of large-area deposition techniques including slot-die coating, inkjet and spray printing, blade coating, and R2R methods is documented in Table 7, reflecting the field’s progress in scalable FPSC manufacturing [192]. 

**Table 7:** Large-area FPSCs using scalable deposition technologies

Structure configuration	Deposition technique	Active area (cm^2^)	Efficiency (PCE %)	Refs
PEN: ITO/Sn_2_/Zn_2_SnO_4_/FA-based Perovskite/spiro-OMeTAD/Au	Blade coating	100.00	15.50	[42]
PET: ITO/SnO_2_/Perovskite/spiro-OMeTAD/Au	Slot-die coating	16.07	15.22	[195]
PET: hc-PEDOT: PSS/oil-PEDOT: PSS/Perovskite/PCBM/BCP/Ag	Meniscus-assisted coating	22.50	16.70	[196]
PEN: hc-PEDOT: PSS/NiOx/Perovskite/PCBM/Ag	R2R printing	15.00	16.15	[197]
PET: ITO/PEDOT: EVA/Perovskite/PCBM/BCP/Ag	Meniscus-assisted coating	36.00	15.21	[198]
PEN: ITO/PEDOT: PSS/PTAA/Perovskite/PCBM/BCP/Ag	Blade coating	1.00	16.61	[199]

## Applications for flexible solar cells

Flexible solar cells quickly emerge as groundbreaking technology with a broad range of uses that transcend traditional photovoltaic systems. Due to their light, flexible, and conformable design, they can easily be integrated into various environments and devices, with ground-breaking energy solutions in a broad spectrum of industries [200]. 

### Flexible solar cells for outdoor items

**Fig. 26:**
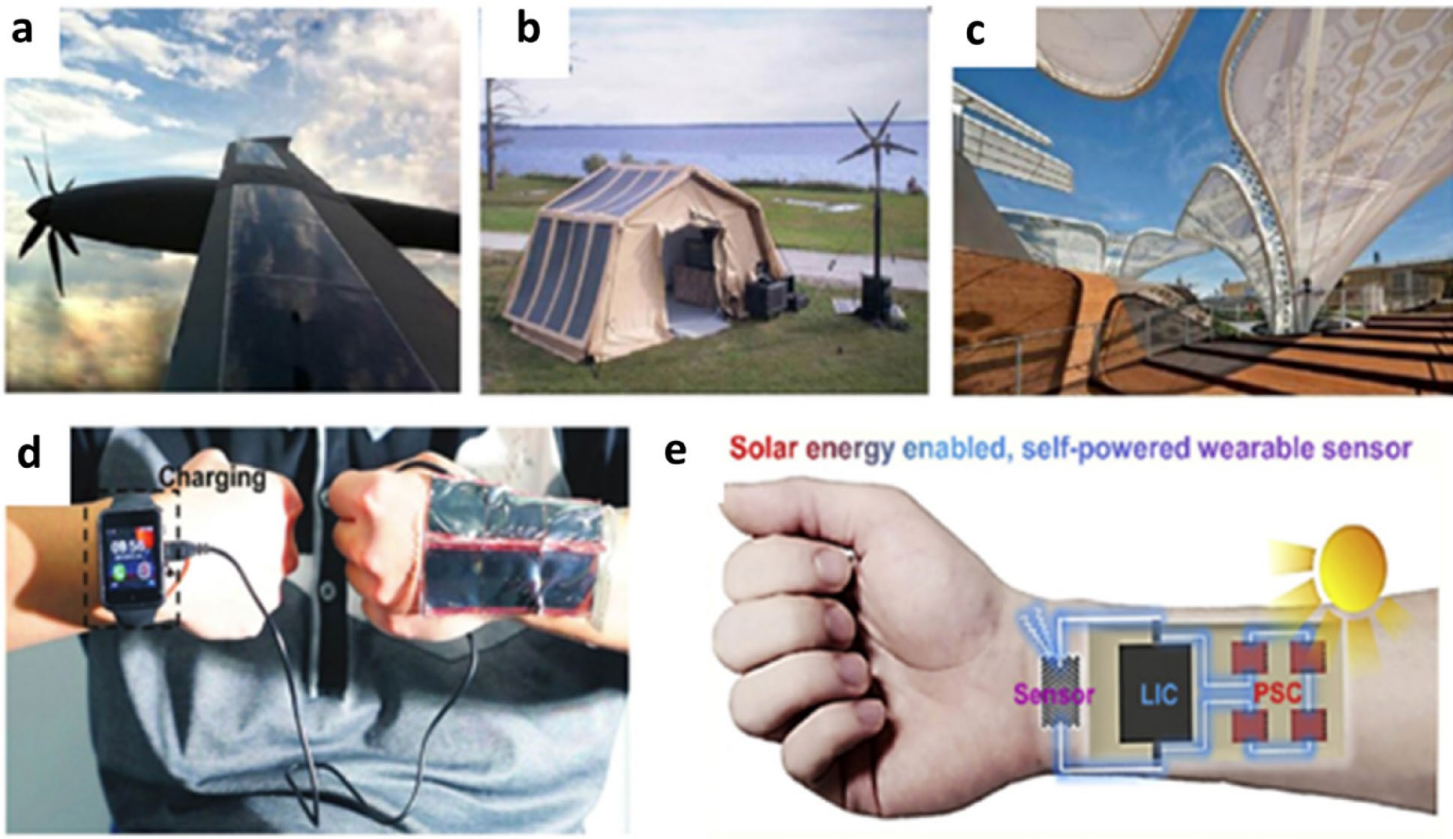
**a** Solar-powered outdoor flight, **b** solar-powered tents. Reproduced with permission from reference [201]. Copyright 2019 Elsevier. **c** smart integrated building, **d** wearable FPSCs used as a smartwatch’s power source. Reproduced with permission from reference [202]. Copyright 2019 Royal Society of Chemistry. e wearable sensor driven by solar energy. Reproduced with permission from reference [203]. Copyright 2019 Elsevier

Figure 8 illustrates solar energy-driven systems for wearables and outdoor applications where Fig. 26a solar cell in thin-film forms is used in outdoor solar-powered flights, providing a clean and renewable energy source that extends the operational time of small planes and drones and eliminates the need for traditional fuels. Figure 26b shows solar-powered tents, where flexible solar panels are incorporated to generate electricity, allowing outdoor consumers and emergency personnel to charge equipment and power machinery in off-grid areas without a power source [201]. Figure 26c displays smart integrated buildings, where thin film solar cells are embedded in building materials such as windows and roofing, supplying electricity to power essential functions such as lighting and climate control while assisting in furthering sustainability goals. Figure 26d presents wearable solar-powered devices, e.g., wristbands and smartwatches, which harvest solar energy for powering small electronics, offering an inexpensive and sustainable alternative to traditional methods of recharging [202]. Irrevocably, Fig. 26e presents solar-powered wearable sensors, used for healthcare monitoring and environmental sensing. These sensors are autonomous in nature, harnessing the solar energy they collect, reducing the need to recharge them on a periodic basis and making them more reliable in real-time, low-power usage [203]. 

### Flexible solar cells for aerospace

**Fig. 27:**
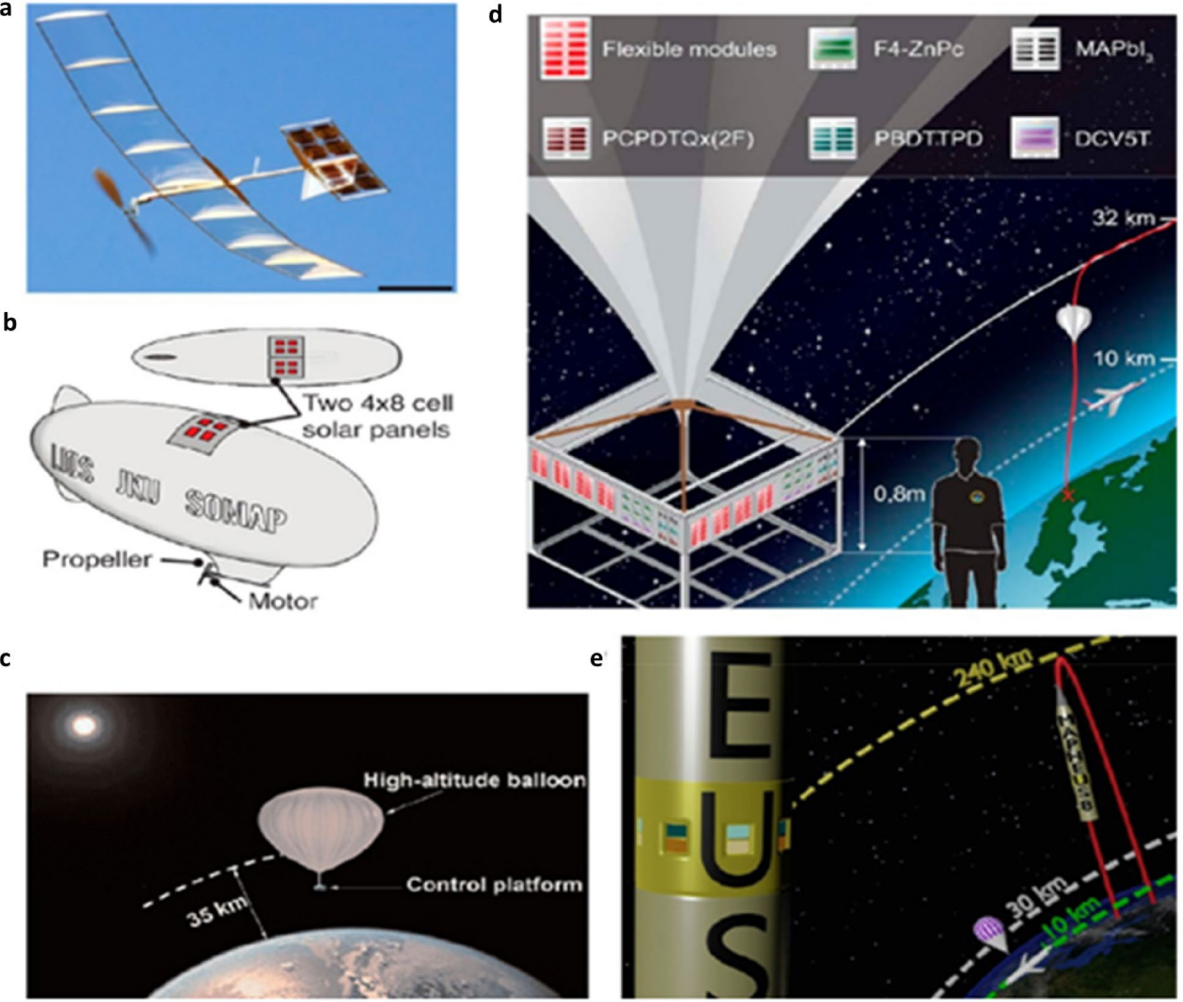
**a** Model plane, **b** model blimp. Reproduced with permission from reference [204]. Copyright 2018 Elsevier. **c** solar cells and fight altitude of OSCAR MISSION. Reproduced with permission from reference [207]. Copyright 2019 American Chemical Society. **d** high-altitude balloon, and **e** the earth, MAPHEUS-8 MISSION. Reproduced with permission from reference [14]. Copyright 2025 IOP Science

The photograph parades a clear vision of utilizing solar energy in aerospace and high-altitude systems. Figure 27a depicts a sample model aircraft with solar power integration. Solar cells have been integrated into the aircraft, which play a crucial role in providing energy for flight in these kinds of systems. The photograph shows that solar energy could be a real power source for small-scale, lightweight, solar-powered aerial systems, enhancing energy efficiency and sustainability in aerospace technology. In Fig. 27b the focus is directed towards a model blimp, another type of solar-powered airship. The blimp features two 4 × 4 cell solar panels and was powered with a propeller and motor system [204]. This configuration demonstrates how solar panels can be combined with airship technology to offer lighter and cleaner means of powering airships for surveillance, communication, or atmospheric research. Figure 27c reflects solar cell deployments in the OSCAR mission, where solar cells are integrated into flight systems of high altitude. Solar power is utilized at an altitude of approximately 35 km, where solar power plays a significant role in sustaining operation in the upper atmosphere, as conventional power generation becomes unavailable. The role of solar power under extreme environmental conditions, where it illustrates how renewable energy could be used by high-altitude platforms for their operations [205]. Figure 27d gives us the vision of flexible solar modules, like materials F4-ZnP, MAPbI_3_, and PBTPTPD, which are tailored for space missions. These solar cells operate at altitudes of 10–32 km, encompassing their adaptability in high-altitude environments. Further reflects the design and ability of these modules to power spaceborne systems or high-altitude balloons, which play a crucial role in conducting scientific experiments and energy generation in near-Earth space. Figure 27e illustrates the MAPHEUS-8 mission, where equipment onboard is solar-powered and is included with a balloon at high altitude at 35 km above Earth’s surface. It depicts the route of the balloon as it circulates around the Earth, illustrating how solar cells provide a renewable source of power for experiments conducted at such heights. The MAPHEUS-8 mission attests to the rising practice of applying solar power using space exploration, and it offers a green alternative for research missions in Earth’s stratosphere. These advancements show the potential for solar power becoming increasingly integral to energy systems of space exploration and high-altitude science [206]. 

### Flexible solar cells for less sun light environment

**Fig. 28:**
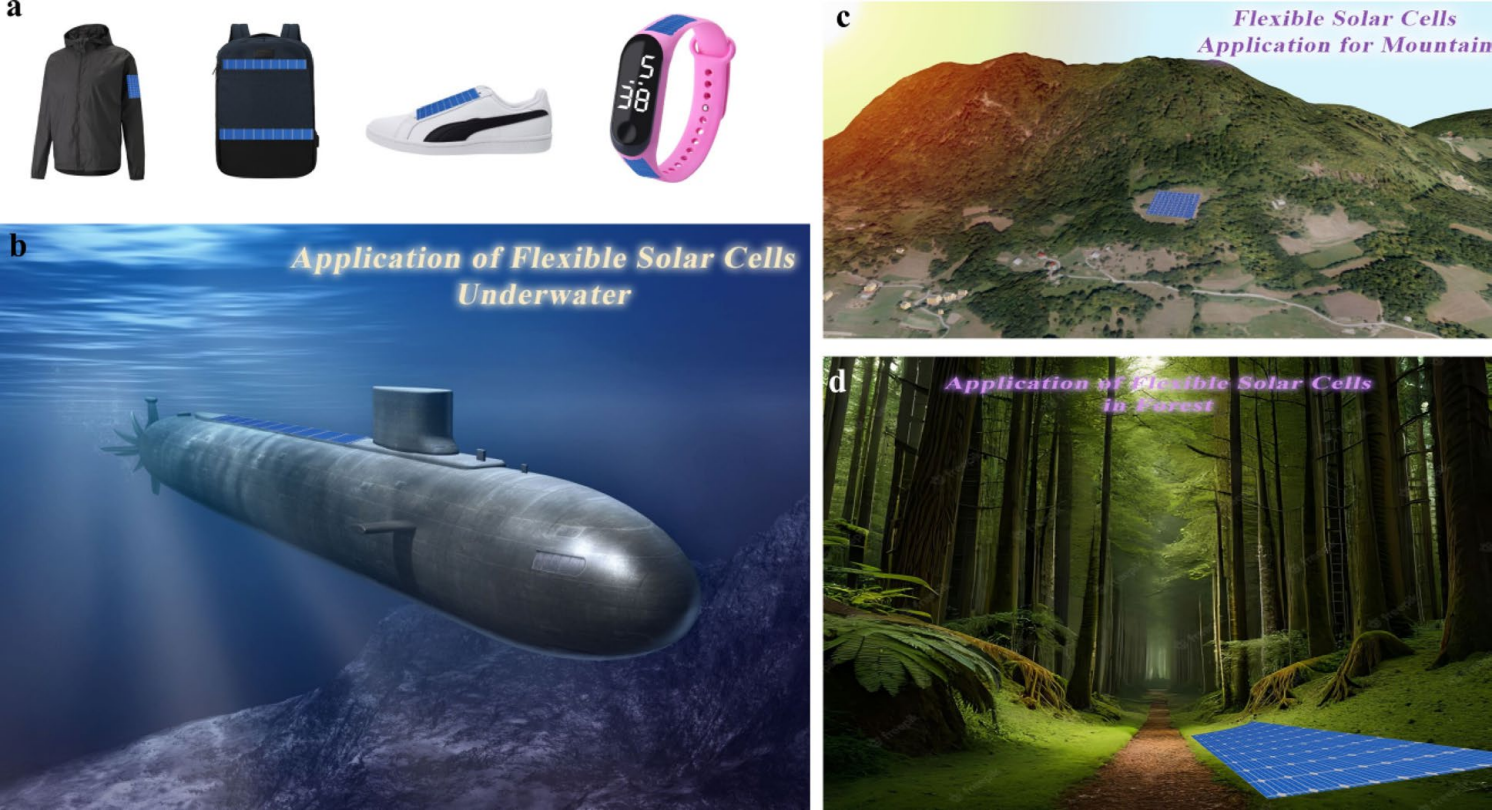
**a** Flexible solar cell technology in the wearable items. **b** Flexible solar cells appointed on Submarine. **c** Flexible solar cell application for mountain cave. **d** Flexible solar cell application for mountain cave

Flexible solar cell technology in wearable products offers groundbreaking opportunities by converting solar energy into electrical power. These cells can be effortlessly integrated into a variety of wearables such as coats, boots, glasses, and watches, thereby enhancing both functionality and sustainability in the realm of wearable technology exhibited in Fig. 28 [208].

#### Flexible solar cells for wearable staff

*Solar Power Jacket*: Wearing a jacket that can power your electronics while you are on the go, all while keeping you warm. You can now generate power during your daily activities by **e**mbedding FPSCs into the fabric of jackets. Mobile phones, tablets, and even compact devices like Bluetooth earphones can be powered by the energy harvested [209]. *Solar Power Bag*: A handbag or backpack that can charge your electronics while you’re on the move. Flexible solar-cell-equipped bags can accumulate energy throughout the day, which can later be used to recharge computers, tablets, and mobile devices [210]. *Solar Powered Hand Watch*: A common item for many people is a wearable wristwatch. By integrating FPSCs into the watch’s face or strap, they can capture light energy to keep the watch running continuously, eliminating the need for frequent battery replacements [117]. *Solar Footwear Boot*: FPSCs can be fitted into boot soles, allowing them to absorb sunlight as you walk or run. For added comfort during the colder months, built-in heating components can be powered by this energy, as can small devices like fitness trackers [211]. The incorporation of FPSCs into wearable items unlocks a world of possibilities, merging fashion with functionality. The application of these solar cells in jackets, boots, glasses, wristwatches, t-shirts, caps, pants, and bags is both vast and exciting. From generating electricity to power small devices to providing backup energy sources during outdoor adventures, we can anticipate even more innovative uses of this technology as it evolves [212]. 

#### Flexible solar cell for submarine

FPSCs offer submarines exploring the oceans depths a renewable energy source by conforming to curved surfaces, unlike rigid panels shown in Fig. 28b. This technology captures solar power even underwater, supplying electricity for onboard systems and equipment, thereby improving efficiency and sustainability in submarine operations. *Source of Clean Energy*: FPSCs use the sun’s energy to generate electricity. Submarines can become more environmentally friendly and sustainable by utilizing this renewable energy source instead of conventional fossil fuels. Submarines can operate continuously on power from FPSCs, eliminating the need to surface for refueling. By allowing submarines to remain submerged for extended periods, this enhances operational efficiency and mission performance [213]. *Reduced Maintenance Costs*: Traditional fuel-powered submarines require routine maintenance and refueling, which can be both costly and time-consuming. Submarines can minimize their dependence on fuel and maintenance demands by integrating FPSCs, leading to significant long-term cost savings. Over the lifespan of the submarine, reduced reliance on fossil fuels and decreased maintenance translate into lower operational expenses. *Opportunities for Research and Exploration*: Flexible solar-powered submarines offer new opportunities for scientific exploration and undersea expeditions. Longer research missions can be conducted uninterruptedly with extended operational capabilities, allowing researchers to dive deeper into the mysteries of the underwater world [214]. *Increased Mission Flexibility*: Submarines equipped with FPSCs offer a broader spectrum of strategic mission planning possibilities due to their reliance on solar energy. They enable more efficient allocation of energy resources, thereby extending operational durations and supporting a greater array of onboard systems. Traditionally, submarines have depended on diesel generators, which contribute to acoustic signatures and environmental pollution. By integrating FPSCs, submarines can operate with enhanced stealth and environmental sustainability, significantly reducing or even eliminating their dependence on conventional generators. The absence of mechanical noise allows for more discreet mission execution, improving the likelihood of successful target engagement [215]. FPSCs possess tremendous potential for harnessing solar energy in underwater vehicles such as submarines. To ensure their optimal performance and extended operational lifespan, it is imperative to carefully consider the influences of environmental conditions and mineral deposits. The sustained efficiency of these solar cells depends on the implementation of robust protective measures, systematic maintenance protocols, and comprehensive mineralogical analysis. We can increase the power generation capability of FPSCs and support the use of sustainable energy in submarines by taking care of these issues [216]. 

#### Flexible solar cell for mountain cave

The use of FPSCs in mountain caves presents a unique setting that introduces both challenges and opportunities. This environment highlights the advantages of deploying solar panels in mountainous terrains, as well as the potential influences of high-altitude conditions on their reliability and operational efficiency. Harnessing solar energy within mountain caverns offers numerous benefits, providing remote and ecologically sensitive locations with a sustainable and resilient power source [217]. This unit will explore the key advantages and prospective applications of solar cells in mountain caves, as illustrated in Fig. 28c. *Portable Power Sources*: Lightweight, portable energy modules can integrate FPSCs. Beyond providing a convenient and eco-friendly power source for charging various devices such as handheld electronics, cameras, or even drones utilized in aerial reconnaissance these packs remain compact and lightweight enough for researchers to effortlessly transport during their expeditions [218]. *Remote Power Generation*: Mountain caves are often situated in isolated regions, far from conventional energy infrastructures. FPSCs can provide a sustainable and reliable source of electricity to power various instruments and illumination systems within these enclosed environments. *Preservation of Natural Environment*: Conventional energy solutions like generators or disposable batteries emit toxic fumes and demand ongoing maintenance. In contrast, solar cells generate clean, renewable energy with minimal ecological footprints, thereby supporting the conservation of the pristine natural surroundings of mountain caves [219]. *Data Collection*: Solar-powered data loggers can monitor a variety of parameters such as temperature, humidity, air quality, and even seismic activity. These autonomous electronic systems can acquire and storing environmental data, yielding valuable insights into scientific research. A sustainable and environmentally responsible method of generating remote power is the deployment of solar cells within mountain caves. With robust design, strategic installation, and proactive maintenance, the resilience and optimal performance of solar cells can be maintained even under the challenging conditions typical of mountainous terrains. By harnessing the abundant energy of the sun, we can contribute to the preservation of the pristine beauty of mountain caves while delivering power in an eco-conscious and sustainable manner [220]. 

#### Flexible solar cell for dark forest

FSC technology has attracted considerable attention in recent years due to its versatility and potential applications across a range of industries. The use of FPSCs in forest environments is one of the more compelling applications. A significant portion of the Earth’s surface is covered with dense forests, which play a crucial role in maintaining ecological balance [221]. This section explores the implementation of FPSCs in forested areas and the environmental impacts of dark forests on the durability of solar cells, as illustrated in Fig. 28d. *Improved Electricity Generation Efficiency*: When compared to conventional crystalline silicon solar panels, FPSCs generate electricity more efficiently in low-light conditions. In dense forests where the canopy may partially filter sunlight, this characteristic is particularly advantageous. FPSCs are an optimal solution for harvesting solar energy in forested environments, as they can maximize energy output even in shaded areas [222]. *Mobility and Adaptability*: These solar cells are highly portable and adaptable due to their light weight and flexibility. They can be rapidly deployed in remote forested regions and offer continuous access to clean, renewable energy. Additionally, because of their versatility, they can be readily installed on a variety of surfaces, including temporary forest infrastructure such as trails and recreational zones. This enhances the accessibility of solar energy in forested environments [223]. Humidity and Moisture: Forests naturally contain higher humidity levels than open areas, which can affect the efficiency and longevity of solar cells. It is critical to select solar panels that are engineered to withstand these conditions and to implement appropriate moisture mitigation strategies to preserve the solar panels stability and resilience. *Variations in Light and Shade*: Forests can create shaded areas that significantly reduce the efficiency of solar panels. Trees and dense vegetation can restrict the amount of direct sunlight reaching the solar cells, thereby diminishing energy yield [224]. To alleviate this issue, solar cells should be strategically positioned and oriented to maximize solar exposure. Forests pose a variety of adverse environmental impacts on FPSCs. While challenges such as shading, debris accumulation, and climatic conditions can influence the stability and performance of solar cells, proactive strategies can mitigate these effects. By understanding and adapting to the forest ecosystem, we can fully harness the potential of FPSCs while coexisting harmoniously with nature [225]. 

## Summary and outlooks for future

**Fig. 29:**
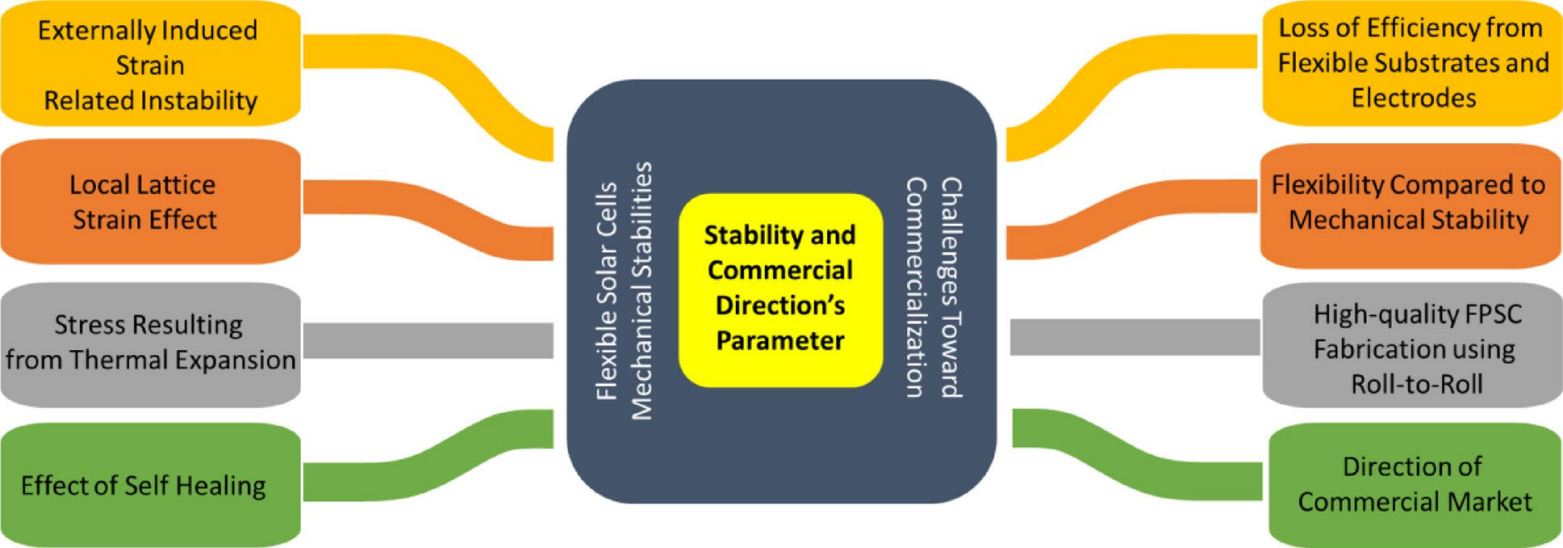
Stability and commercialization parameters of FPSCs

FPSCs possess distinctive qualities such as a high power-to-weight ratio, exceptional flexibility, and comparatively low production costs. However, their real-world deployment remains a few steps away. The stability and commercial viability of flexible PPSCs are pivotal for their widespread integration. The limitations affecting the operational durability and practical utilization of flexible PPSCs are being assessed on Fig. 29. Factors including long-term efficiency, scalability for large-scale manufacturing, economic feasibility, material compatibility with flexible substrates, and operational reliability under varied environmental conditions play a critical role in the successful commercialization of FPSCs [226]. 

### Loss of efficiency from flexible substrates and electrodes

Efficiency remains the paramount criterion for evaluating solar cell performance. Although various FPSC technologies have been introduced, flexible devices still underperform in comparison to their rigid counterparts. The primary discrepancy in device architecture and performance deficit can be attributed to the nature of flexible electrodes and substrates. ITO electrodes on PET and glass exhibit sheet resistances of approximately 60 and 10 Ωsq^− 1^, respectively. Elevated resistance levels typically lead to reductions in both Jsc and fill factors (FF), thereby diminishing overall efficiency. Enhancing the optical transparency of flexible substrates represents the second critical improvement area. For instance, a conventional FTO/glass substrate demonstrates a transmittance as high as 92%, whereas an ITO/PET substrate only achieves around 78% [227]. As a result of the insufficient generation of photo-induced electrons, the Jsc remains limited. Third, polymer substrates are generally rougher than glass, and the deposited electrodes typically exhibit lower electrical conductivity. The crux of efficient photogenerated current lies in achieving a high-quality perovskite layer. However, owing to the pronounced surface roughness, CTLs and the overlying perovskite components tend to develop suboptimal morphologies. To further enhance the performance of FPSCs, it is essential to incorporate electrodes that offer both superior optoelectronic properties and optimized surface morphology [228]. 

### Flexibility compared to mechanical stability

**Fig. 30:**
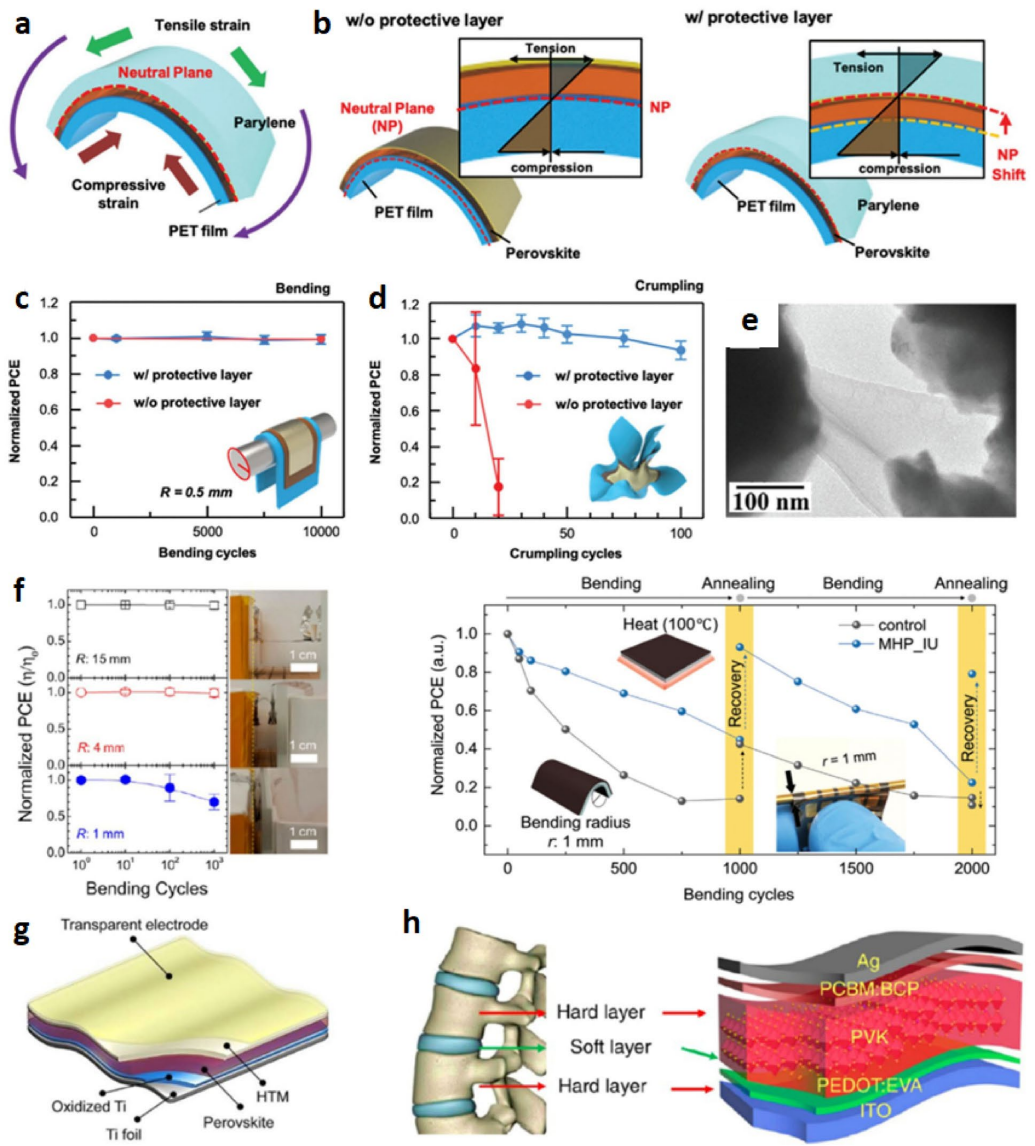
**a** Schematic visualization of the neutral mechanical plane. **b** Photographic comparison of flexible devices fabricated with and without parylene protective coatings. **c** Normalized PCEs of FPSCs as a function of bending cycles at a 0.5 mm radius (left) and crumpling cycles (right), with and without the inclusion of a parylene-based encapsulation layer. Reproduced with permission from reference [233]. Copyright 2019 The Royal Society of Chemistry. **d** TEM image showing cross-linked perovskite grain structures. **e** Evolution of PCE in FPSCs subjected to repeated bending cycles (radius = 1 mm), comparing devices with and without the incorporation of PU–PDMS-IU. Reproduced with permission from reference [234]. Copyright 2022 John Wiley & Sons Inc. **f** Assessment of the mechanical flexibility of FPSCs under bending stresses at curvature radii of 15 mm, 4 mm, and 1 mm. **g** Titanium-based FPSCs featuring a transparent top contact composed of Cu/Au layers. Reproduced with permission from reference [235]. Copyright 2018 American Chemical Society. **h** In the bionic architecture of FPSCs, PEDOT: EVA serves as HTL, functioning analogously to cartilage by dissipating mechanical stress and protecting the integrity of the perovskite layer. Reproduced with permission from reference [198]. Copyright 2020 Springer Nature

As previously mentioned, the layers are subjected to mechanical deformation when bent, as illustrated in Table 8 and Fig. 30a. Consequently, the bendability of the device is, to some extent, dictated by its bending endurance. Enhanced flexibility is always advantageous for real-world applications. However, mechanical robustness remains a critical challenge for both perovskite layers and transparent electrodes. TCOs (such as ITO and FTO) are commonly employed on polymer substrates, despite their inherent brittleness. Repeated bending can induce microfractures, ultimately leading to device degradation. Moreover, convex deformation can adversely affect the perovskite layer. According to research, the formation of cracks within the perovskite film can significantly impair both structural integrity and electrical performance [229]. Recently, innovative architectural designs aimed at accommodating mechanical strain have shown tremendous potential for practical applications, effectively mitigating the damage caused by repeated flexing. Figure 30b–g illustrates various structural configurations and experimental outcomes that support these advancements. One promising approach involves repositioning the perovskite layer from the substrate to the neutral mechanical plane, thereby enhancing mechanical durability while enabling ultra-flexibility and foldability [230]. 

Interfacial engineering using ethylene vinyl acetate (EVA) has been employed in FPSCs to enhance mechanical pliability and adhesive strength, drawing inspiration from bioinspired structural frameworks show in Fig. 30h. Through this adhesive interface, mechanical stress can be uniformly distributed and effectively dissipated [231]. As a result, the PEDOT: EVA layer significantly reduces localized strain on the perovskite layer, thereby minimizing deformation and maintaining structural integrity even after 7000 flexural cycles at a 3 mm radius. In addition, several ultra-flexible transparent electrodes have been proposed as alternatives to conventional transparent conductive oxides (TCOs) for use in FPSCs. These innovations aim to enhance both mechanical resilience and electrical performance. Table 8 highlights a selection of these cutting-edge materials, showcasing their potential to replace brittle TCOs while supporting the flexibility demands of next-generation solar devices [232]. 

**Table 8:** The bending capability of FPSCs

Structure of FPSC device	Conversion efficiency (%)	Minimum bending radius (mm)	Flexibility (Cycles)	Efficiency retention (%)	Refs
PEN: ITOHfO_x_/SnO_2_/Perovskite/Spiro-OMeTAD/Au	21.10	10.0	2000	93.2	[236]
PEN: ITO/NiO_x_/Perovskite/Spiro-OMeTAD/Au	18.50	7.0	1200	80.0	[237]
PEN: ITO/NiO_x_/Perovskite/PCBM/BCP/Bi_2_Te_3_	18.16	4.0	1000	95.0	[238]
PET: Au/mesh/PEDOT: PSS/Perovskite/C_60_/BCP/Cu/Parylene	13.60	2.0	10,000	100	[233]
PET: Ag mesh/ZnO/Perovskite/Spiro-OMeTAD/MoO_3_/Ag	16.47	3.0	1500	90.0	[239]
AgNWs/ZnO/TiO_2_/Perovskite/Spiro-OMeTAD/MoO_3_/Ag	17.17	4.0	2000	77.0	[240]
PET: Graphene-GQDs/Perovskite/PCBM/Al	15.00	4.0	3000	70.0	[241]
PET: PEDOT: PSS/Perovskite/C_60_/BCP/Cu/Parylene	17.03	2.0	5000	92.0	[242]
PET: ITO/FI-SnO_2_/Perovskite/Spiro-OMeTAD/Au	21.00	5.0	20,000	80.0	[236]
PET: ITO/PEDOT: EVA/Perovskite/PCBM/BCP/Ag	19.87	5.0	7000	95.0	[243]
PET: ITO/SnO_2_/Perovskite/Spiro-OMeTAD/Au/PDMS/SU-8	20.04	5.0	300	86.0	[244]
PET: ITO/PTAA/Perovskite/C_60_/BCP/Cu	21.76	2.5	25,000	90.0	[245]
PEN: ITO/PTAA/Perovskite/C_60_/BCP/Cu	18.74	2.5	1000	95.0	[246]
PEN: ITO/PTAA/Perovskite/C_60_/BCP/Cu	18.74	4.0	5000	70.0	[247]
PEN: ITO/PTAA/PFN-Br/Perovskite/C_60_/BCP/Cu	20.00	2.5	10,000	73.0	[248]
Willow glass: ITO/PTAA/Perovskite/C_60_/BCP/Cu	19.72	3.4	10,000	95.8	[249]
Cellulose paper: TiO_2_/Ultrathin Ag/TiO_2_/CPTA/Perovskite/Spiro-OMeTAD/Au	13.19	1.0	1000	95.8	[250]
NOA_63_/PEDOT: PSS/Perovskite/PCBM/Ga-In Eutectic Alloy	10.83	1.0	1000	90.0	[251]
Mica: ITO/PEDOT: PSS/Perovskite/PCBM/BCP/Ag	18.00	5.0	5000	92.0	[252]
PDMS: Ti/TiO_2_/Perovskite/PTAA/Graphene	15.00	12.0	1000	83.0	[253]
PDMS: TFSA-Doped Graphene/PEDOT: PSS/Perovskite/PCBM/Al	18.30	12.0	5000	85.0	[254]
PI: SWNT/MoOx/PTAA/Perovskite/C_60_/BCP/Cu	15.20	0.5	10,000	100	[255]
PEN: Graphene/MoO_x_/Perovskite/C_60_/BCP/LiF/Al	16.80	2.0	5000	85.0	[256]

### High-quality FPSCs fabrication using R2R technique

As mentioned earlier, small-area FPSCs have reached power conversion efficiencies as high as 25.09%. Nonetheless, scaling up to larger areas continues to present a significant challenge due to persistent issues in controlling pinholes and maintaining uniform film quality. The maximum PCE only reached 17.55% for a large-area FPSC with a 31.20 cm^2^ effective area. Furthermore, small-area device manufacturing methods are inherently incompatible with scaled fabrication particularly in addressing the demand for commercially scalable, cost-efficient, and high-velocity continuous processing capabilities [257]. The advantages offered by R2R processing make it one of the most attractive and feasible methods for producing flexible devices on a large scale. First of all, solution-based methods such as the use of organic PVs are more advantageous for commercial manufacturing than vapor deposition, as they are significantly more cost-effective and less intricate in terms of fabrication equipment requirements [258]. R2R platform is also fully compatible with standard printing approaches such as inkjet, spray, and blade coating that have demonstrated reliability in fabricating rigid FPSCs. With deposition protocols already well-documented, achieving desirable film characteristics generally involves fine-tuning rather than reinventing the entire deposition strategy. In contrast to earlier coating systems, continuous processing requires the precise calibration of the moving speed of flexible substrates [259]. The performance of photovoltaic devices is strongly influenced by the quality of each layer, which in turn relies on accurately controlling perovskite film formation during high-speed processing. Although perovskite films have been successfully prepared through one- or two-step processes, the resulting efficiencies remain subpar. To enhance film uniformity, further investigation is essential in key areas such as humidity resilience, full-process R2R fabrication, ambient eco-compatible processing, additive engineering, solvent engineering, and additive manufacturing. To improve the effectiveness of FPSCs produced via R2R manufacturing, it is necessary to examine parameters that are intricately linked to film crystallization kinetics [260]. 

### Environmental stability and encapsulation

Environmental stability remains the primary challenge in the commercialization of FPSCs. Exposing devices to environmental factors such as water, oxygen, and ultraviolet light promotes perovskite layer degradation and organic CTL degradation, both of which are highly stress sensitive. Degradation finally results in diminished performance and even device failure. Effective encapsulation is one of the key techniques employed to negate this degradation since it serves as a barrier that prevents moisture and oxygen penetration into the device. The use of flexible polymer substrates in FPSCs, however, presents a problem since the materials employed are far more permeable to both moisture and oxygen compared to the traditional glass substrates [261, 262]. This requires the creation of advanced encapsulation schemes for ensuring the long-term stability of such devices. Recent advances in encapsulation have been directed to the use of dense inorganic films, i.e., SiO_2_, to reduce water vapor transmission rates to as low as 8.63 × 10^−3^ g m^−2^ day^−1^ under very severe conditions of 37.8 °C and 100% relative humidity. While this has resulted in enhanced device lifetime, there still remains penetration by moisture and oxygen through sealing layers and electrical connections. In addition, the other parameters such as the composition of perovskite layer, crystallinity, homogeneity of the film, and quality of CTL and electrode deposition must also be optimized with utmost care to overcome the cause of degradation in FPSCs [263, 264]. 

**Fig. 31:**
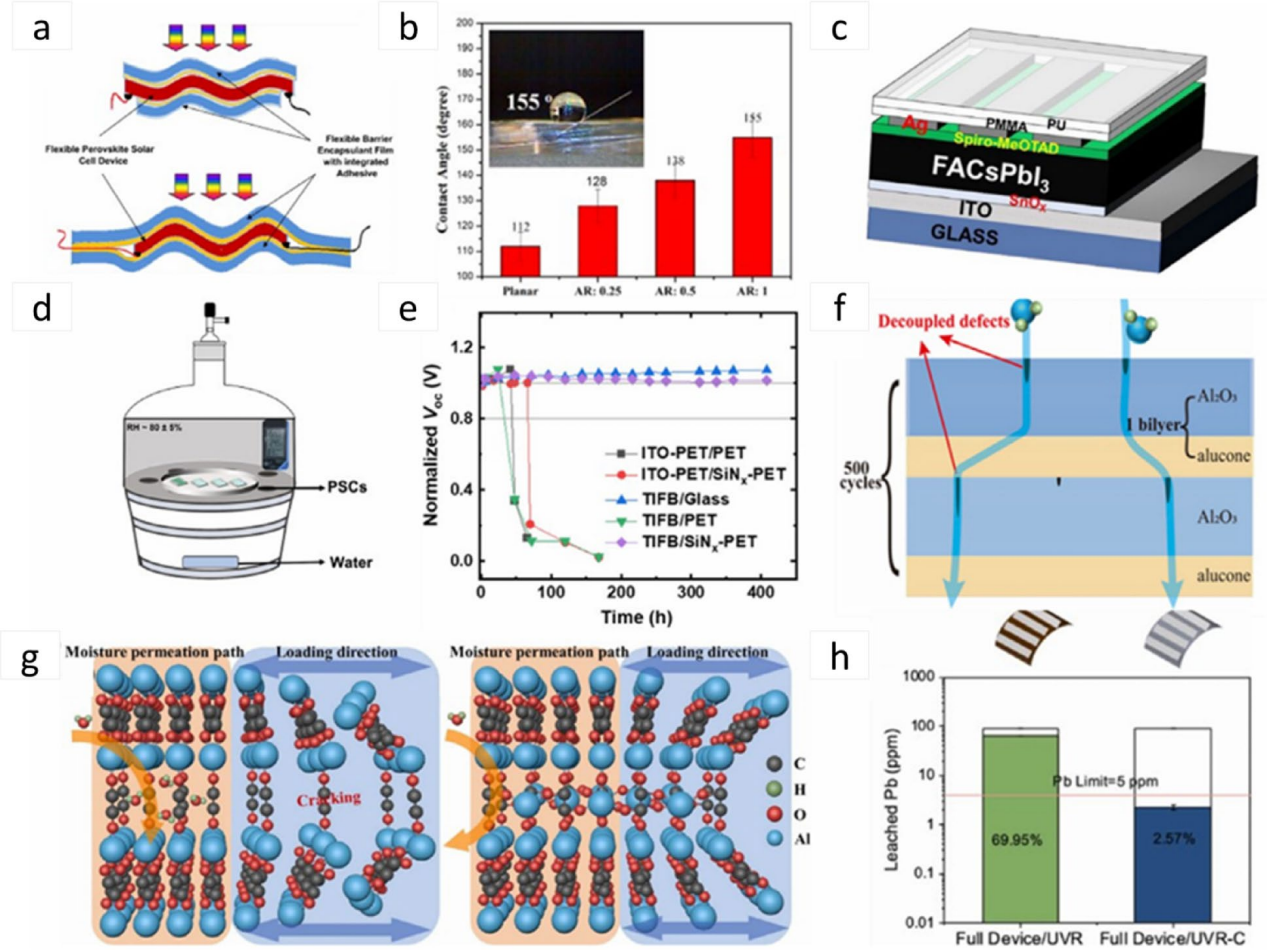
**a** Schematic diagram of the encapsulation structure with view barrier and 3 M adhesive. Reproduced with permission from ref [265]. Copyright 2015, Elsevier. **b** Static contact angle measurement on PDMS layer with varying aspect ratios, with the inset showing a water droplet on the nanocone PDMS surface. Reproduced with permission from ref [266]. Copyright 2015, American Chemical Society. **c** Diagram illustrating the complete device structure. **d** Schematic diagram of storing the device in a humidity-controlled chamber. Reproduced with permission from ref [267]. Copyright 2024, The Royal Society of Chemistry. **e** Device stability testing after exposure to extreme conditions (60 °C, 90% relative humidity) for over 400 h. Reproduced with permission from ref [268]. Copyright 2024, Wiley-VCH GmbH. **f** Schematic illustrating the defects passivation and water permeation suppression in the double-layer encapsulation structure. Reproduced with permission from ref [269]. Copyright 2020, Elsevier. **g** Mechanism illustrates the flexibility and barrier efficacy of the PEMLD-AlCO hybrid structure. Reproduced with permission from ref [269]. Copyright 2023, Elsevier. **h** Comparative investigation of lead leaching in devices with two distinct encapsulation layers. Reproduced with permission from ref [270]. Copyright 2022, Elsevier

Scientists have focused on more flexible and durable encapsulation methods to reverse the limitations of traditional rigid encapsulation materials. For example, Weerasinghe et al. employed view barrier as a plastic barrier material, in conjunction with 3 M adhesive, to significantly enhance the moisture resistance and ruggedness of FPSCs (Fig. 31a) [265]. Similarly, Tavakoli et al. employed a nanocone-arrayed PDMS layer, which improved light transmission and enhanced device efficiency up to 13.14% by applying the hydrophobicity of PDMS to enhance moisture resistance (Fig. 31b) [266]. Other materials, such as PMMA and hydrophobic polyurethane (PU), were also tested, with devices retaining 92% of initial efficiency after 1500 h at 80 ± 5% relative humidity (Fig. 31c, d) [267]. While as effective as adhesive-based encapsulation methods, it does complicate device flexibility and production. To overcome these hurdles, researchers have turned to more sophisticated techniques, such as depositing a SiNx barrier layer on top of the ITO layer through chemical vapor deposition (CVD). This encapsulation method with built-in transparent electrode ensures flexibility and stability, maintaining 90.90% of the original efficiency of the device after 400 h at 60 °C and 90% relative humidity (Fig. 31e) [268]. Sophisticated deposition techniques like CVD and atomic layer deposition (ALD) have been capable of producing uniform, dense, and pinhole-free films that can further enhance the protective ability of the encapsulation. Wang et al., for instance, used plasma-enhanced ALD at 50 °C to deposit a high-waterproof encapsulation film and reduced the water vapor transmission rate to 1.3 × 10^−5^ g m^−2^ day^−1^ (Fig. 31f) [269]. 

Other advancements include the use of plasma-enhanced molecular layer deposition (PEMLD) to create organic-inorganic hybrid films with outstanding barriers and flexibility characteristics. The films achieved a water vapor transmission rate of 1.44 × 10^−5^ g m^−2^ day^−1^ and maintained 95% of their barrier function even after experiencing 10,000 bending cycles at a radius of 3 mm (Fig. 31g) [270]. Despite all these developments, long-term bending and stretching-induced structural damage remains an issue as it can lead to leakage of lead, a life-threatening issue that retards commercialization of FPSCs. To reverse the effect, high-acid cation exchange resin encapsulants were made. The encapsulants effectively confine over 90% of Pb^2+^ ions under severe weather conditions, preventing leakage of lead and enhancing the stability of the device during operation (Fig. 31h). Although much progress has been made toward the development of encapsulation technologies for FPSCs, more research and development must be undertaken to make these devices as durable and stable as they need to be for large-scale commercialization.

### Direction of commercial market

**Fig. 32:**
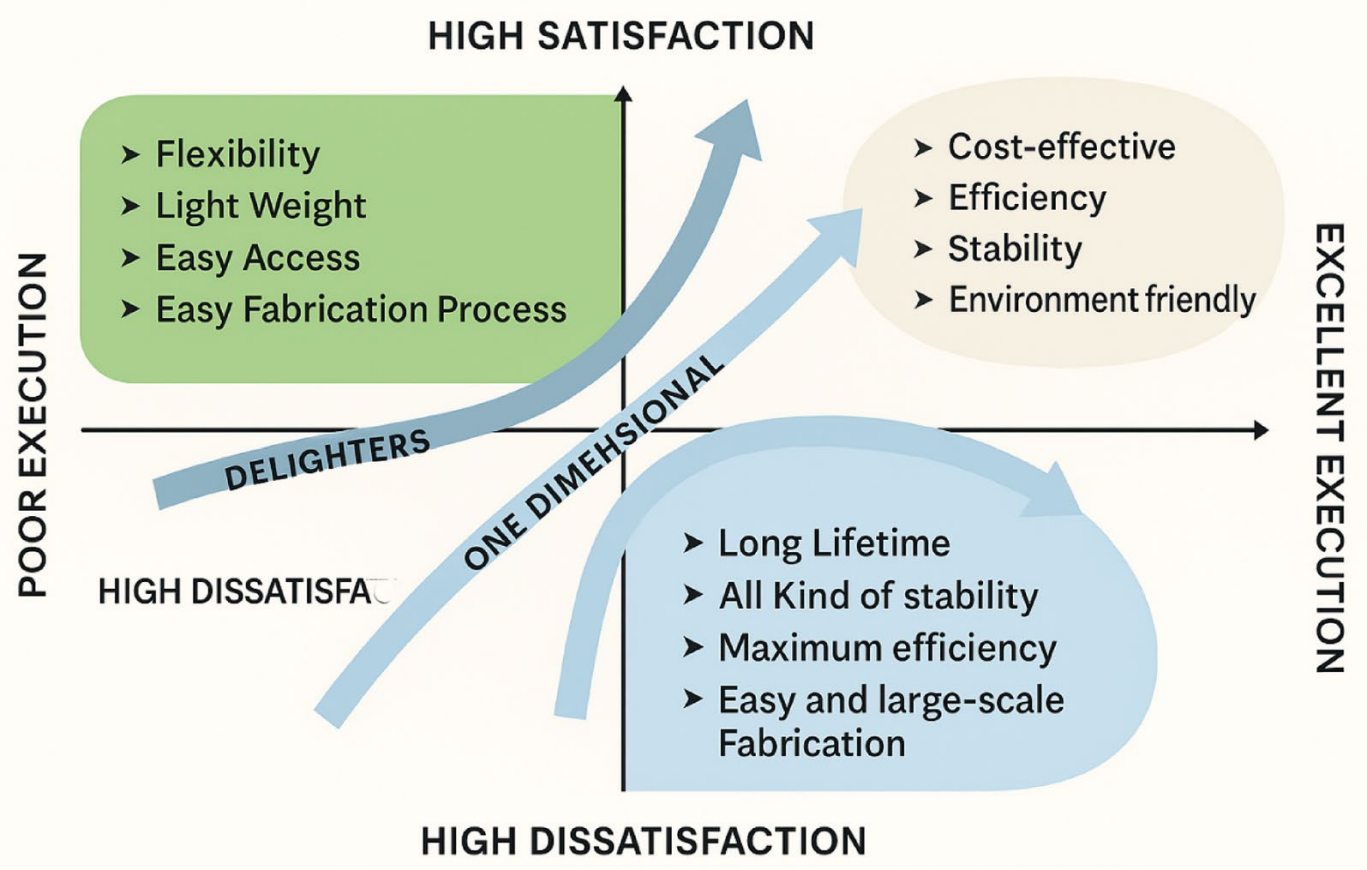
Successful FPSCs commercialization

FPSCs, the most promising contender among the next-generation PV technologies, have progressed rapidly over the past decade. The primary advantages of FPSCs lie in their cost-effectiveness and exceptional efficiency, which make them particularly appealing for large-scale commercialization, as illustrated in Fig. 32. The projected material cost of a perovskite module is approximately 20 USD/m^2^, with a total production cost around 40 USD/m^2^ less than half the cost of conventional silicon solar cells. While flexible devices incur a slightly higher expenditure (33.53–62.29 USD/m^2^) compared to their rigid counterparts, they offer a significantly broader scope of applications due to their mechanical adaptability [271]. 

In the realm of advanced photovoltaic systems, FPSCs have seen swift progress and are regarded as one of the most potential-rich options developed in recent years. Crucial features such as operational durability, efficiency, ease of production, and affordability significantly contribute to the commercial viability of FPSCs. However, varying experimental conditions such as humidity, temperature, and encapsulation techniques used by different researchers make stability results inconsistent and difficult to compare objectively. In contrast, PCE is a well-defined parameter that can be evaluated according to established standards, unlike stability-related aspects such as device longevity and degradation kinetics. It is therefore essential to standardize the testing protocols for PSC stability evaluation, particularly focusing on mechanical durability, thermal resilience, device hysteresis, and exposure-related stability under light, moisture, and oxygen tailored to each fabrication methodology [272]. The overhead diagram serve to visually articulate, how various PSC characteristics should be prioritized for successful commercialization. In practical terms, the overall cost including materials, labor, overhead, and capital investment ultimately defines the market viability of FPSCs. Efficiency and stability losses during scale-up from small cells to larger modules present a major hurdle in bringing FPSCs to market. Continued efforts in device architecture optimization and materials innovation remain promising avenues for advancing FPSCs in the emerging PV market landscape [273]. 

### Mechanical strength

Transparent conductive oxide (TCO) and metal electrodes, which are currently utilized in FPSCs, exhibit limited mechanical durability. However, promising developments have emerged in addressing this challenge by exploring TCO-free electrode alternatives. Graphene, carbon nanotubes, WO_3_–Ag–SnO_2_ composites, and metallic meshes of Cu, Ni, or Ag nanowires are leading contenders. Compared to TCO, these materials offer enhanced mechanical resilience. It is important to note, however, that FPSCs employing TCO-free architecture may initially exhibit reduced PCE. Thus, to ensure manufacturing feasibility, it remains essential to further optimize the PCE of TCO-free FPSCs [274]. Graphene and its derivatives have been identified as promising substitutes for conventional metal electrodes (Al, Cu, Ag, Au, etc.) owing to their advantageous work functions (5.1 eV), excellent mechanical integrity, and robust environmental stability. However, graphene and its derivatives do not yet match the electrical conductivity of traditional metals. To evaluate the viability of FPSCs without transparent conductive oxides or hole transport materials, an in-depth analysis of recombination processes at the graphene perovskite interface is decisive. Moreover, carbon-based freestanding FPSCs are anticipated to emerge due to the exceptional mechanical endurance of carbon materials [275]. 

### Environmental consistency

Achieving long-lasting stability in FPSCs largely depends on effective macro and micro encapsulation techniques. Future development of macro-encapsulation will emphasize the design of highly transparent, ultra-low water vapor transmission rate films, which will be employed to hermetically seal FPSCs using materials such as polyisobutylene, epoxy, and organic-inorganic hybrid composites. To enable cost-effective and low-temperature encapsulation, novel techniques such as spin-coating must be further innovated and optimized. Conversely, microencapsulation is anticipated to immobilize low-dimensional perovskites, PbSO_4_, Pb(PO_4_)_2_, and other densely packed, chemically stable perovskite crystals. Even with advanced encapsulation strategies, ion diffusion continues to pose a serious concern in FPSCs. For instance, the perovskite absorber layer is subject to degradation by diffusing metals particularly silver as Ag ions migrate into the perovskite structure, forming AgI [276]. In this context, carbonaceous materials can contribute significantly to reinforcing the intrinsic durability of electronic components. Compared to traditional electrodes, nanocarbon-based materials may exhibit superior optical transparency, electrical conductivity, and structural compactness. Conspicuously, solution-processable double-walled carbon nanotubes have recently been investigated as potential replacements for conventional ITO layers. According to recent studies, carbon-based materials hold great promise for realizing next-generation, high-efficiency FPSCs. Therefore, we posit that in the coming years, the synergy between perovskite and carbonaceous materials will become a central focus in photovoltaic research [277]. 

### Cost of production

The overall cost of production, particularly material expenditure, must be rigorously assessed and prioritized in future commercialization strategies, alongside advancements in PCE and FPSC stability. The strategic direction includes finding low-cost alternatives to Spiro-OMeTAD, PTAA, and gold in n-i-p FPSCs. In contrast, p-i-n devices continue to depend on methanofullerene compounds like PCBM, C60, and other fullerene-based materials [278]. However, the innovation of alternative ETMs to substitute these materials remains a pressing research frontier. Given their abundance, intrinsic environmental resilience, and mechanical robustness, nanocarbon materials emerge as highly promising candidates for engineering low-cost, durable, and efficient FPSCs. By minimizing the complexity of the fabrication process, the development of ETM- and/or HTM-free architectures could lead to further cost reductions. In addition, carbonaceous materials enable the fabrication of bifacially illuminated FPSCs, which can be integrated seamlessly into architectural elements such as windows, rooftops, and facades thereby expanding the effective area for power generation and enhancing solar energy utilization, ultimately balancing production costs [279]. 

### Fabrication on large scale

As device dimensions scale up, series resistance inherently increases, leading to a decline in PCE. This efficiency drop is primarily attributed to the proliferation of pinholes and morphological defects in the perovskite layers. As the perovskite layer scales up, the chance of defects increases, and the film quality, specifically the compactness and uniformity of layers like the ETM, HTM, and absorber, often decline across larger areas. This is particularly true for solution-processed fabrication techniques. Consequently, the PCE loss during scaling may not follow predictable trends [280]. To mitigate efficiency deterioration, novel and compatible fabrication methodologies must be explored. It is important to highlight that the size of today’s large-area FPSCs remains considerably smaller than that of silicon and CdTe modules. Therefore, a standardized definition of large area for FPSC manufacturing is required. Lessons learned from the scalable production of other photovoltaic platforms such as silicon, organic, and CdTe solar cells should inform this process. To retain high PCEs while scaling, the implementation of serially interconnected FPSC modules may offer a practical solution for delivering consistent and elevated power outputs [281]. 

### Toxicity

Many high-performance FPSCs currently available on the market unfortunately incorporate lead-based compositions, which are well known for their environmental toxicity. Beyond efforts in Pb recovery and recycling, intensified research is essential for the development of lead-free alternatives. Presently, Sn-, Sb-, and Bi-based FPSCs are under investigation; however, these variants are hindered by either limited stability or suboptimal efficiency. Semitransparent FPSCs are worth developing in addition to the significant difficulties and perspectives. Such properties might expand the applications of FPSCs, such as building-integrated photovoltaics [282]. As a compact and stable energy source, FPSCs are increasingly compatible with cutting-edge flexible electronics like e-skins. Integrating them with wearable displays, sensing modules, and power units opens pathways for real-time health tracking and smart medical diagnostics. In addition, the rapid advancement of the Internet of Things is opening new opportunities, where indoor low-light energy harvesting using FPSCs is expected to become a prominent research direction. We think that the FPSC will demonstrate flexibility in the upcoming PV market given the expanding and optimistic state of perovskite photovoltaics research today [283]. 

## Performance evaluation and development trends

**Fig. 33:**
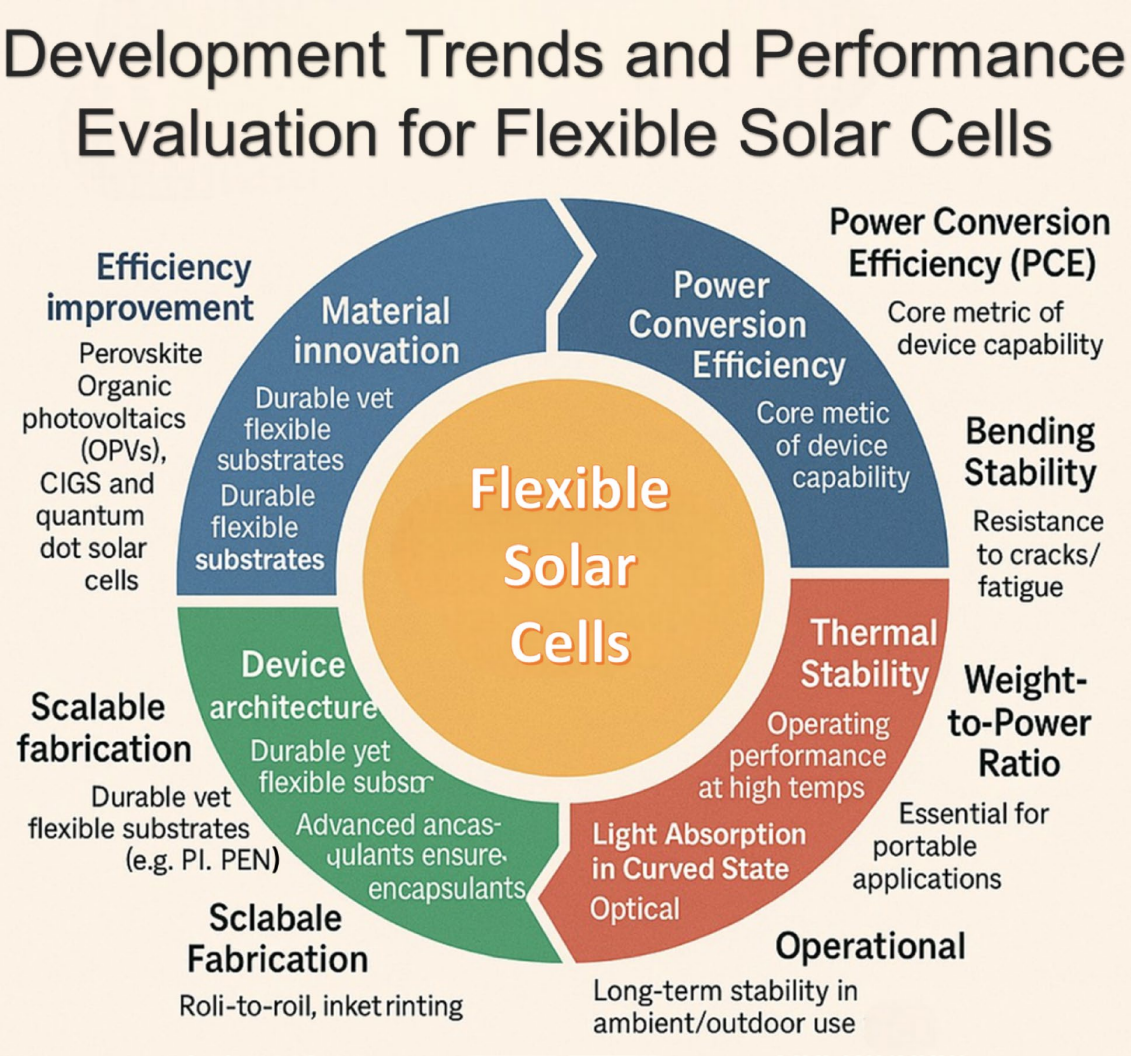
Development trends and performance evaluation of flexible solar cells

FPSCs represent a development in next-generation photovoltaic devices to harvest energy from curved, wearable, and portable surfaces. Figure 33 presents key development trends and performance metrics for FPSCs. It highlights core aspects such as PCE, thermal and bending stability, and weight-to-power ratio. Material innovation, including durable and flexible substrates, plays a vital role in improving efficiency. Scalable fabrication methods like R2R printing support large-scale production [284]. Overall, design and architecture focus on enhancing device durability, optical performance, and operational stability in real-world conditions. Lightweight and flexible, they stand out as potential candidates in making electronics sustainable and charging the gadgets off-grid. Table 9 provides an overview of essential performance indicators for FPSCs such as power conversion efficiency, flexibility under mechanical stress, power-to-weight ratio, and heat resistance, enabling a comprehensive evaluation of their practical viability [285]. 

**Table 9:** Performance evaluation metrics of flexible solar cells

Metric	Definition	Benchmark values (2024)	Refs
Power conversion efficiency	% of sunlight converted to electricity	>25% (PSC), ~ 18% (CIGS), ~ 13% (OPV)	[286]
Bending radius	Minimum radius at which device retains function	< 5 mm	[287]
Flex cycles (durability)	Number of bending cycles before 10% drop in efficiency	>5000 cycles	[288]
Weight-to-power ratio	Watts per gram	1–10 W/g (depending on material)	[289]
Thermal stability	Performance at elevated temperature	Up to 85–100 °C	[290]
Operational lifetime	Device stability under real-world conditions	>5 years (encapsulated)	[291]
Light absorption (bent state)	Retained optical efficiency under curved state	>90% of flat-state absorption	[292]
Transparency (for BIPV)	Light transmission in semi-transparent devices	10–50% visible light	[293]

### Material improvements in solar systems

The dawn of flexible solar has brought with it sustained rapid technological steady coaxed by the material side, which should be highly efficient and mechanically adaptable. FPSCs serve highest in the candidate list, Power Conversion Efficiencies exceeding 25% are achieved while rendering great flexibility and resisting bending stresses well. Low-temperature-solution processing renders it compatible with R2R type manufacturing processes [294]. Furthermore, Organic Photovoltaics present additional benefits like eco-friendliness, light-weighting, and printing onto flexible plastic substrates, perfectly suited for semi-transparent and wearable applications. Meanwhile, CIGS and quantum dot solar cells come with long-term stability in excellent performance for huge area installation and flexing. It maintains high absorption efficiencies over very wide spectral ranges with solid real-world performance; thus, these materials have been established as a backbone in realizing next-generation flexible solar devices [295]. 

### Electrode and interface engineering

Transparent and flexible conductors are generally considered silver nanowires, graphene, and carbon nanotubes to form low-resistance/high-transparency front contacts for efficient light harvesting, while the same must remain mechanically flexible. Interface passivation, on the other hand, and buffer-layer optimization are needed to limit the recombination of charge carriers at such important junctions. In fact, these strategies really stabilize an interface and improve PCE as well, which in turn advances flexible photovoltaic technologies [296]. 

### Substrate and encapsulation development

Materials such as polyimide, polyethylene naphthalate, and ultrathin glass are often preferred for their intrinsic mechanical sturdiness and high thermal stability in flexible applications. In order not to degrade devices due to environmental factors, atomic layer deposition or multilayer barrier approaches are applied as encapsulation methods. To ensure long-lasting operational stability and performance, advanced encapsulation methods are employed that lower water vapor transmission to less than 10^− 6^ g/m^2^/day [297]. 

### Scalable manufacturing

The production of FPSCs at scale becomes economical through R2R processing and slot-die coating and inkjet printing methods which provide continuous and high-throughput manufacturing. These methods excel at manufacturing large-area devices since they optimize material utilization while decreasing waste generation. A central question is what approaches can utilize low temperature processing the goal being to preserve the structural and therefore functional integrity of polymer substrates. By operating below the thermal deformation limits of flexible polymers, we are promoting lightweight, flexible devices while progressing from laboratory- to equipment-scale manufacturing [298]. 

### Application diversification

The speed of flexible solar cell technologies into various applications is rapidly accelerating across many disciplines. In wearables and e-textiles, lightweight and conformable power sources present opportunities. For example, there is a long-established relationship between aerospace and drone technology that relies on weight and flexibility when harvesting energy in a moving dynamic context. Furthermore, IoT systems can utilize its small form-factor for self-powering sensors and devices to extend autonomy and additional functionality. Building-integrated photovoltaics represents a new growth area where these new flexible modules can be embedded into building surfaces in a way that complement modern design patterns, while adding energy generation [299–301]. 

### Future perspectives

**Fig. 34:**
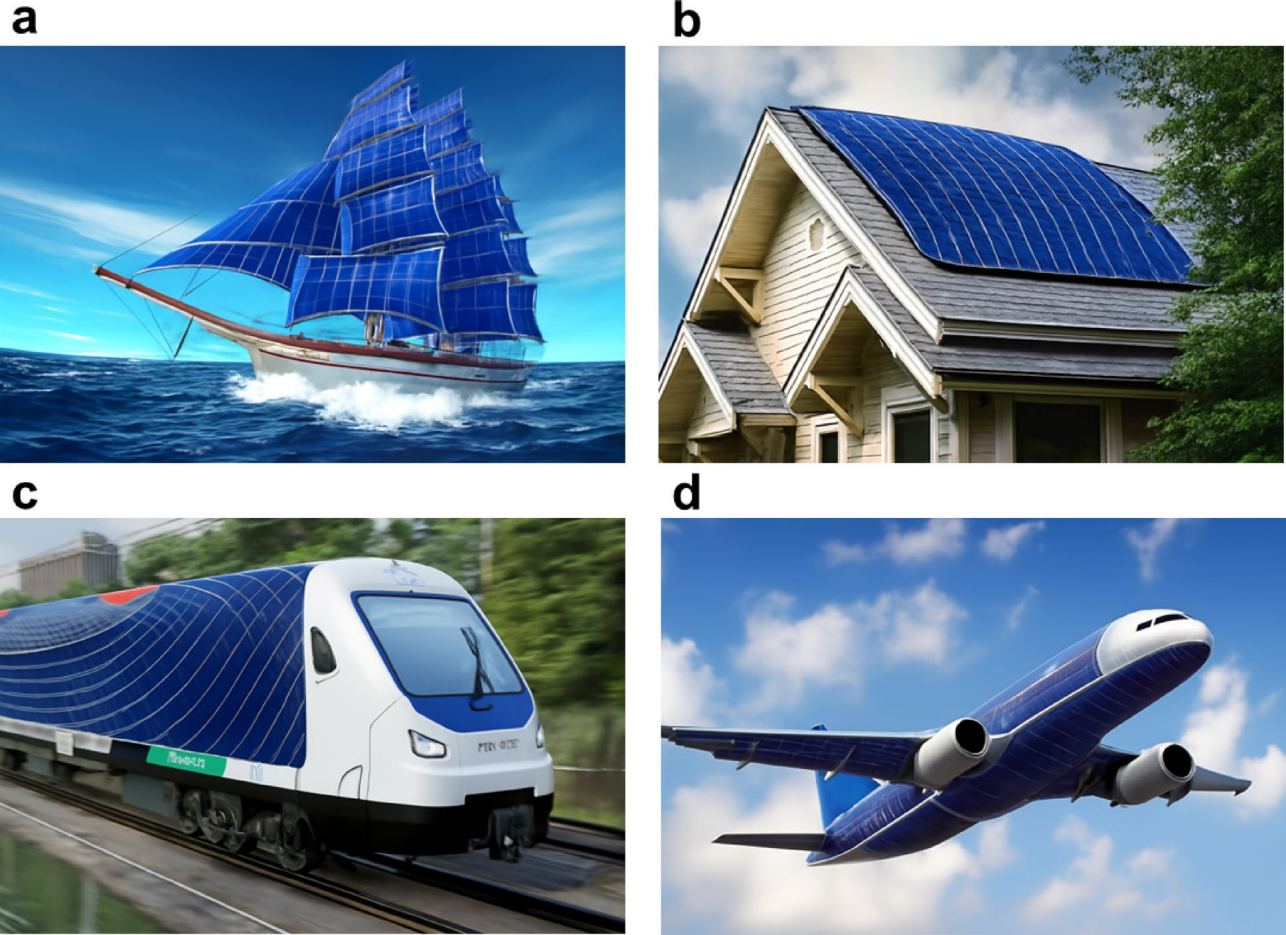
The usages of flexible solar cells in **a** Sail, **b** House, **c** Train, **d** Airplane

The future of thin-film solar cells is extremely promising with the recent advances in perovskite solar technology. Japan has led the advancement of flexible large-area perovskite solar cells, which now have applications across a broad spectrum of industries from residential rooftops to transportation trains, airplanes and maritime travel sailboats show in Fig. 34. The uses of flexible solar cells are vast. For example, the integration of such solar cells on boat sails can provide a source of clean energy for marine transport, while the installation of flexible solar cells on residential buildings can reduce household dependency on grid energy significantly. Similarly, solar aircraft and trains can reduce carbon emissions and fuel consumption by a significant amount, making transportation cleaner.

These cells are more efficient than ever before, and the perovskite materials have been up to 30% efficient significantly higher than the traditional 20% efficiency of silicon-based cells. With such high efficiency as well as material flexibility, there are new possible opportunities for incorporating solar energy into many different platforms.

China already has cost-effective flexible solar cells, so these are optimal for massive applications. But the perovskite solar cells China is making available now have stability problems, which will limit their long-term performance and sustainability as well. Japan’s perovskite-based flexible solar cells are slightly more expensive, but these are renowned for the quality and life, and regardless of any weather situation, they are better.

Japan and China are currently the only two in the global market for flexible perovskite solar cells. The two countries have varying competencies Japan on producing high-end, long-duration products, and China on prices. In the future, with the development of technology, we will see more countries join the game in the market to spur innovation and reduce costs. Flexible perovskite solar cells in the future will revolutionize the renewable energy sector with innovative, high-efficiency solutions for widespread applications. This innovation promises to help industries as varied as transportation to domestic energy use and further, ensuring a world where solar energy becomes a part of everyday life.

## Data Availability

Not applicable.
